# Inflatable porous organic crystals

**DOI:** 10.1038/s41563-025-02393-6

**Published:** 2025-11-03

**Authors:** Alexios I. Vicatos, Leigh Loots, Gundo Mathada, Joanna Drwęska, Agnieszka M. Janiak, Leonard J. Barbour

**Affiliations:** 1 Department of Chemistry and Polymer Science, University of Stellenbosch, Stellenbosch, South Africa; 2 Faculty of Chemistry, Adam Mickiewicz University, Poznań, Poland; 3 Department of Chemistry, School of Natural Sciences, University of Lincoln, Lincoln, UK

**Keywords:** Crystal engineering, Organic molecules in materials science, Actuators

## Abstract

The relationship between changes in the macroscopic dimensions of a solid and its environmental conditions such as temperature or pressure can be rationalized at the molecular level. The controllable conversion of such external stimuli to mechanical energy can be exploited to construct mechanical or electromechanical devices, which are sometimes required to operate in extreme environments. Here we describe predominantly unidirectional expansion and contraction of an acicular porous molecular crystal owing to gas uptake or release. Using complementary in situ structural and photomicrographic techniques, we have obtained molecular-level insights that correlate macroscopic linear expansion of the crystal by up to 10% with the application of gas-specific pressure. We also demonstrate that the expansion of the needle axis with pressure can be modelled using the well-known Langmuir–Freundlich equation, thereby providing a convenient means of relating pressure and guest-induced linear expansion within a bounded continuum.

## Abstract (web summary)

Reversible and unidirectional expansion of an acicular porous molecular crystal is observed with gas uptake. Using in situ structural and photomicrographic techniques, a molecular-level insight is obtained that correlates macroscopic linear expansion of the crystal to the application of gas-specific pressure.

## Main

Many structurally flexible porous materials swell reversibly in response to the uptake and release of small volatile compounds in their surroundings. For example, carpenters must anticipate and compensate for cross-grain expansion and contraction of wooden boards with fluctuations in relative humidity^1^. When immersed in liquid water, clays expand anisotropically^2^ whereas hydrogels swell isotropically^3^. Coal beds also swell isotropically under high pressures of methane (CH_4_) or carbon dioxide (CO_2_) gas^4^. Indeed, the influence of external stimuli on strain in one or more of the three principal directions has been studied extensively for a wide variety of responsive materials. Although crystals are generally considered to be brittle chemical cemeteries^5^ in which the components are frozen into static three-dimensional arrays, recent research efforts have revealed that some are capable of a surprising level of dynamic behaviour^6^. Exerting control over dynamic strain with a view to develop new potential applications of crystals as mechanical, electrical or optical components has been termed crystal adaptronics^7^. This relatively young area of research is primed for the discovery of material properties, accompanied by the development of innovative approaches to quantification and molecular-level elucidation of the underlying dynamic mechanisms.

In recent years, much attention has been focused on the structure–property relationships of porous materials, particularly metal–organic frameworks^8^ and covalent organic frameworks^9^. However, the lesser-known porous molecular crystals have yielded several surprising findings; some examples of these from our own work, and that are relevant to the current report, include transient porosity (that is, the transport of guest molecules within a flexible host framework devoid of permanent channels^10^) and low-temperature uptake and release of water vapour^11^. The porosity of crystalline molecular materials may result from inefficient packing (extrinsic voids that form between host molecules) or molecular design (intrinsic voids in host molecular cavities)^12^.

**Fig. 1:**
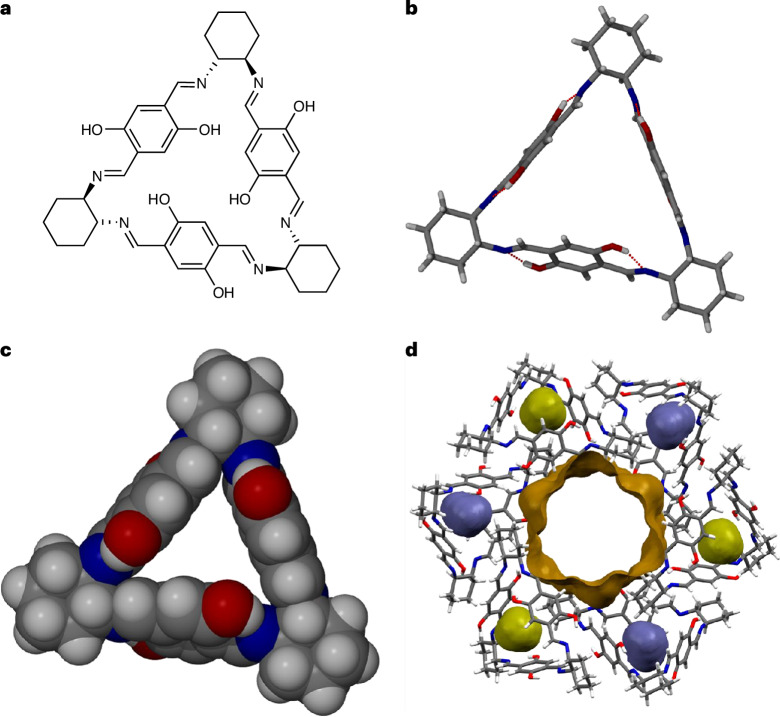
Various representations of trianglimine **T1**. **a**, Schematic of **T1**. **b**, Capped-stick model showing intramolecular hydrogen bonds (dotted red lines), which rigidify the open conformation of the molecule. **c**, Space-filling model showing the molecular cleft of **T1**. **d**, Trigonal arrangement of six molecules of **T1** to form a 1-nm-wide 1D extrinsic channel (brown surface) propagating along [001], and six hybrid intrinsic–extrinsic 0D voids; the blue and green surfaces represent the two crystallographically unique void spaces at the centroids of the **T1** molecules. The models shown in **b**–**d** are based on the structure of anhydrous crystals of **T1**, determined at 298 K. Atom colours: white, hydrogen; grey, carbon; blue, nitrogen; red, oxygen. All void spaces were mapped using a probe with a radius of 1.5 Å.

Our research on the host–guest chemistry of molecular crystals has primarily centred on cyclic hosts, such as metallocycles and organic macrocycles, which are designed to contain clefts or cavities that can form intrinsic or hybrid intrinsic–extrinsic zero-dimensional (0D) lattice voids. In this context, we recently reported a detailed in situ structure–property study of a trianglimine macrocycle **T1** (ref. ^11^; Fig. 1), which crystallizes to form both one-dimensional (1D) channels and seemingly inaccessible 0D voids. The 0D voids are initially too small to accommodate gas molecules such as CO_2_ if the crystal structure remains rigid. However, by using a combination of in situ techniques, we demonstrate that CO_2_ gas molecules can be forced into the 0D voids under pressure. This process is facilitated by structural flexibility, which allows the voids to swell individually in response to increasing gas pressure without damaging the overall integrity of the crystal. The swelling mechanism causes one of the crystallographic axes to undergo pressure-dependent expansion, which also manifests macroscopically, thereby providing a new type of stimulus for crystal adaptronics.

## Crystal packing and guest-accessible space

The enantiopure chiral host **T1** (Fig. 1a–c) crystallizes to form acicular crystals with space group *R*3; the asymmetric unit comprises two host molecules, each situated on a general position. Application of three-fold rotational symmetry yields a hexameric ring (Fig. 1d) in which the molecules associate via relatively weak dispersion interactions. The rings stack according to unit-cell translational symmetry to form conceptually infinite 1-nm-wide channels that propagate along [001], which is also the needle axis of the crystal. In addition to the extrinsic space represented by open channels, a map of the probe-accessible space (Supplementary Text 3) reveals the presence of two crystallographically distinct 0D cavities in the structure, each of which is centred on the aperture of a trianglimine host molecule. At relative humidity levels above 55%, the crystals of **T1** readily and reversibly absorb water; the water molecules aggregate in the open channels, which contain hydrophilic binding sites, but do not access the 0D voids.

Since the open-channel crystal form of **T1** (**T1**_**0**_) is stable in the absence of water molecules, we investigated its ability to also absorb CO_2_ gas at various pressures. We first evaluated the potential guest-accessible space in **T1**_**0**_ in silico using structural data (Cambridge Structural Database REFCODE BIJLIT01) obtained during our previous work^11^. The two crystallographically unique 0D voids are formed in a similar fashion, and therefore, it is only necessary to describe one such assembly (Supplementary Text 4). The trianglimine molecule is relatively rigid; strong intramolecular hydrogen bonds within the bridging arms orient the hydroquinone rings approximately perpendicular to the mean plane of the molecule, and there is limited capacity for the rotation of linkers to consume the cavity. The molecular aperture is capped at each end by a cyclohexane moiety of a neighbouring host molecule, one oriented edge on and the other almost coplanar with the trianglimine. This arrangement results in the formation of 0D hybrid intrinsic–extrinsic voids that are close to but isolated from the open channels and, thus, seemingly impervious to guest intrusion. Moreover, under ambient conditions, the probe-accessible volumes of the two crystallographically distinct voids are 37 Å^3^ and 45 Å^3^, as calculated using MSRoll^13,14^. According to the 50% rule of thumb^15^, each guest molecule in an inclusion compound nominally requires space equal to at least twice its van der Waals volume. Although originally formulated for solvates, we believe that this rule can also be applied to estimate the upper limit of guest occupancy for the inclusion of a gas in a porous crystalline material^16^. On the basis of this postulate, neither of the discrete voids in **T1**_**0**_ is large enough to accommodate a molecule of CO_2_, for which we estimate a van der Waals volume of 33.3 Å^3^ (Supplementary Text 5). However, using the program Mercury^17^ and using a probe radius of 1.5 Å, we determined the total probe-accessible volume per extrinsic channel per unit cell of **T1**_**0**_ to be 1,032 Å^3^. This is sufficient space to accommodate a maximum uptake of approximately 2.6 molecules of CO_2_ per **T1** molecule^15^; if we assume that the crystal structure is rigid, this value represents the expected experimental limit of CO_2_ uptake by the material.

## Gas sorption analysis

**Fig. 2:**
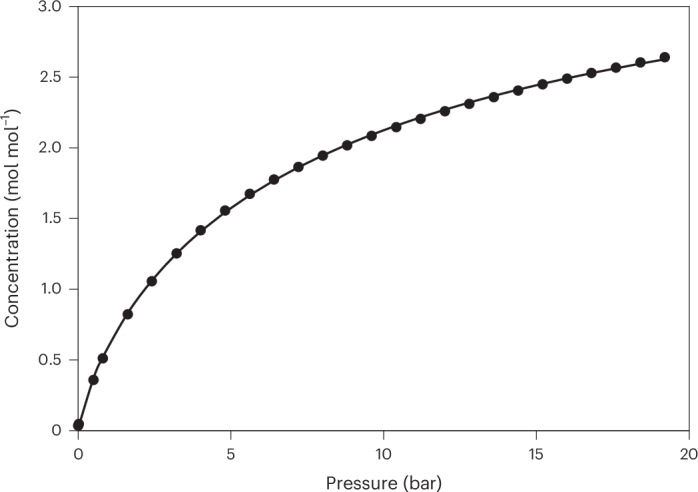
Sorption isotherm for the uptake and release of CO_2_ gas by **T1** crystals at 20 °C. Experimental absolute adsorption (filled circles) data for CO_2_ in the pressure range of 0–20 bar. The solid line represents the corresponding optimized Langmuir–Freundlich model^21^ for adsorption. Gas uptake is shown in moles of gas per mole of **T1** to rationalize the data in terms of guest–host stoichiometries. Source data

A sorption/desorption isotherm was recorded for the inclusion of CO_2_ by **T1** crystals at 20 °C over the pressure range of 0–20 bar (Fig. 2 and Supplementary Text 6). The isotherm follows a type I trajectory^18^ with negligible hysteresis, and the absence of inflections in the data implies that the uptake of CO_2_ does not require a breathing or gate-opening structural transformation^19,20^. At almost 20 bar (the upper pressure limit of the instrument), gas loading reached 2.64 molecules of CO_2_ per host molecule. However, from the shape of the isotherm, it is apparent that CO_2_ uptake does not plateau at 20 bar, and modelling the data using the Langmuir–Freundlich equation^21^ predicts a maximum guest–host molar ratio of *n*_max_ = 3.98 at infinite pressure (Supplementary Table 1). Since the CO_2_ capacity of the material exceeds our prediction based on structural data, and the isotherms do not suggest an abrupt change in the crystal structure of the host, we undertook a series of in situ variable-pressure crystallographic studies with a view to locate the preferred binding sites of the gaseous guest and to elucidate the reason for the seemingly incongruous CO_2_ sorption data.

## In situ crystallography

On the basis of the shape of the sorption isotherm, in situ variable-pressure single-crystal X-ray diffraction data were recorded for crystals of **T1** in the pressure range of 0–32 bar of CO_2_ (experimental details are provided in Supplementary Text 7.1 and selected data are summarized in Table 1 and Supplementary Table 3). Typical for in situ crystallographic characterization of porous crystals under gas pressure, it was not possible to reliably model the sorbed gas molecules. However, we were able to extract important and detailed structural information regarding the pressure-dependent response of the host structure to gas loading. At 32 bar, the unit-cell volume of **T1**_**C32**_ was approximately 5.3% larger than that determined under vacuum (Supplementary Fig. 8a). Although published in situ structural data are still relatively rare, the slight unit-cell expansion of a porous crystalline material due to guest inclusion is intuitive^22–25^, and has been observed previously (Supplementary Text 8). Relative to the structure under vacuum, the crystallographic *a* axis initially expands slightly (peaking at 2 bar) and then contracts by approximately 1.5% over the range of 2–32 bar (Supplementary Fig. 8b). Concomitantly, the crystallographic *c* axis expands dramatically (Table 1 and Supplementary Table 3), stretching by 8.6% between 0 and 32 bar (Supplementary Fig. 8c and Supplementary Text 7.3).

**Table 1:** Salient parameters obtained from the in situ crystallographic analysis of **T1** crystals under CO_2_ gas pressure at 300 K

Experimental conditions		Unit-cell parameters			Channels	Cavities^b^	
*P* (bar)	Structure code^a^	*a* (Å)	*c* (Å)	*V* (Å^3^)	*V*_ch_ (Å^3^)	*V*_1_ (Å^3^)	*V*_2_ (Å^3^)
0	**T1**_**0**_	50.581	9.656	21,394	996	38	48
1	**T1**_**C01**_	50.777	9.681	21,616	990	43	64
2	**T1**_**C02**_	50.829	9.754	21,823	996	52	74
4	**T1**_**C04**_	50.762	9.804	21,879	999	55	77
8	**T1**_**C08**_	50.462	10.064	22,195	1,051	69	86
16	**T1**_**C16**_	50.044	10.299	22,337	1,118	83	81
32	**T1**_**C32**_	49.810	10.488	22,535	1,192	85	83

^a^The subscript in each structure code (generalized as **T1**_**G***x*_) indicates both gas and pressure, where **G** (guest) is **C** for CO_2_ and *x* is the pressure in bars.

^b^*V*_1_ and *V*_2_ refer to the volumes of the two crystallographically unique cavities.

Note: data were first recorded for the crystal subjected to vacuum (**T1**_**0**_), after which the pressure was increased before each subsequent analysis. Supplementary Text 7 provides a complete description of the experimental procedures.

It is important to note that a crystal structure determination only yields a time-averaged structural model for the irradiated part of the sample under a well-defined set of experimental conditions. Thus, the model might not accurately represent a precise molecular arrangement at a specific location within the crystal at any moment. Below, we first discuss the overall changes in structural features of the time-averaged models derived from our variable-pressure single-crystal X-ray diffraction data (Supplementary Text 9), after which we propose a molecular-level mechanism to account for structural adaptation by **T1** crystals to gas inclusion.

**Fig. 3:**
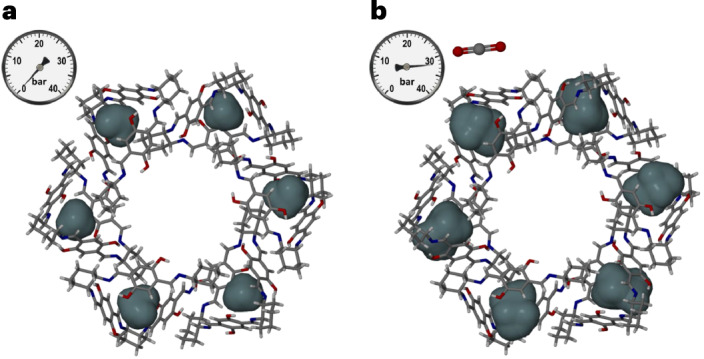
Adaptation of 0D voids of **T1** crystals to CO_2_ inclusion. **a**,**b**, Perspective views along [001] of **T1**_**0**_ (**a**) and **T1**_**C32**_ (**b**). **T1** molecules are shown in capped-stick representation and probe-accessible (*r*_probe_ = 1.5 Å) cavities as blue surfaces.

Surprisingly, over the pressure range of 0–32 bar, the 1D channels of **T1**_**C***x*_ expand in volume by 19.7% (Supplementary Table 3 and Supplementary Fig. 8d) despite the slight contraction of *a* and expansion of *c* by 8.6%. Probe-accessible maps (Supplementary Video 1) show that both crystallographically distinct 0D voids grow substantially and in unison with increasing pressure, reaching average volumes of 84 Å^3^ in **T1**_**C32**_ (Fig. 3). In sharp contrast to the structure of **T1**_**0**_, this provides sufficient space to accommodate one CO_2_ molecule per cavity, adding additional CO_2_ uptake capacity of one molecule per **T1** molecule. Indeed, electron counts based on a difference electron density map (Supplementary Text 10) strongly suggest that each of the 0D voids is almost fully occupied by a molecule of CO_2_ at 32 bar. Owing to the conformational rigidity of the trianglimine molecule, a substantial expansion of a 0D void in its crystal structure can only occur if the capping cyclohexane groups of neighbouring **T1** molecules are drawn or pushed away. This can be achieved by both tilting and shifting the host molecules along [001], thereby shortening the *a* and *b* axes and elongating the *c* axis (Supplementary Video 6). Thus, our proposed mechanism for the insertion of a CO_2_ molecule into a 0D void is as follows. The gas molecule enters the channel but cannot bind strongly to the channel walls. However, coordinated thermal motion of the **T1** molecules creates a short-lived pore that both enlarges a void and connects it to the channel. Under favourable geometric and dynamic conditions, the CO_2_ molecule can exploit this fleeting metastable state to enter the void (analogous to ingestion by phagocytosis), thereby locking the local arrangement of **T1** molecules to accommodate guest binding. The energetic penalty for moving the host molecules apart is offset by the omnidirectional dispersion interactions between the guest and host molecules that define the void. The resulting increased periodicity along [001] with increased gas loading creates convex indentations in the channel walls, thereby also increasing the volume of the channel as a direct result of accommodating guest molecules in the 0D voids (Supplementary Video 4). Indeed, under 32 bar of CO_2_ pressure, the channels expand to enclose a volume of 1,192 Å^3^ per unit cell; according to the 50% rule, this expansion is sufficient to increase the capacity of each channel to three molecules of CO_2_ per **T1** molecule. This channel capacity, in combination with the inclusion of up to one CO_2_ molecule per 0D void, is consistent with the maximum total uptake capacity of four CO_2_ molecules per **T1** molecule, as predicted by applying the Langmuir–Freundlich model to the experimental sorption data.

**Fig. 4:**
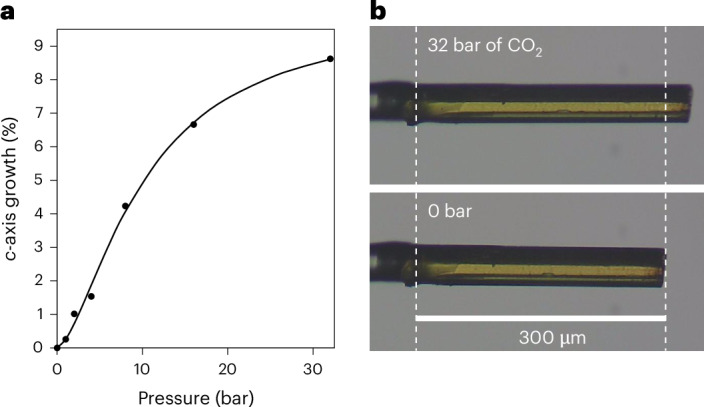
Elongation of a crystal along [001] in response to CO_2_ gas loading. **a**, Plot of percentage elongation of the crystallographic *c* axis of **T1** crystals with increasing CO_2_ pressure. Percentage elongation is calculated relative to the length of *c* at 0 bar. The filled circles represent the experimental data and the optimized Langmuir–Freundlich fit is shown as a solid line. **b**, In situ photomicrographs of a crystal of **T1** in its forms **T1**_**0**_ (bottom) and **T1**_**C32**_ (top). The dashed vertical line on the left indicates where the crystal is attached to a glass fibre and the dashed vertical line on the right facilitates observation, relative to **T1**_**0**_, of gas-induced elongation along [001].

Although the variable-pressure crystallographic models show an incremental expansion of 0D voids, it is important to note that a specific void can assume only one of two possible states, that is, it can be either occupied or unoccupied at any moment. At 0 bar, all the voids are unoccupied, and at the maximum loading, all of them are presumably occupied, that is, these two extreme situations are nominally represented by our structural models for **T1**_**0**_ and **T1**_**C32**_, which also provide the lower and upper limits of the length of the *c* axis. At equilibrium, we can consider any one of the intermediate states **T1**_**C***x*_ to be a solid solution comprising a dynamic but randomly distributed ensemble of **T1** monomers and **T1**‧CO_2_ heterodimers, where the **T1**:**T1**‧CO_2_ ratio is dependent on the external CO_2_ pressure *x*. At the unit-cell level, the periodicity of the *c* axis is governed by the spacing along [001] of two successive supramolecular assemblies, each consisting of a ring of 6 **T1** molecules that form a part of a column that encloses a 1D channel. For zero and full loading, both local and global *c*-axis lengths are governed by the periodicities of two (**T1**)_6_ or (**T1**‧CO_2_)_6_ rings, respectively. However, at any equilibrium loading, the ensemble of 12 molecules considered can instantaneously assume any of the stoichiometries (**T1**)_*n*_:(**T1**‧CO_2_)_12–*n*_ (*n* = 0 to 12). Each ensemble has a local periodicity dictated by *n*, as well as by the states of surrounding ensembles, and each unit cell is furthermore traversed by three columns of host molecules. The crystal structures of **T1**_**C***x*_ represent the time-averaged permutations of the various configurations described above, and the global length of the *c* axis, thus, approximates a pressure-dependent continuum between the two extremes measured for **T1**_**0**_ and **T1**_**C32**_. Owing to the link between pressure and guest–host stoichiometry, there also exists a link between the measured length of the *c* axis and the overall guest occupancy, which probably accounts for the Langmuirian trend in the pressure-induced elongation of *c* (Fig. 4a and Supplementary Text 11). Indeed, the elongation of *c* with pressure for the structures **T1**_**C***x*_ in the range *x* = 0–32 bar was modelled using the more versatile Langmuir–Freundlich equation, which predicts the maximum extension at full loading of approximately 10%, relative to the guest-free form. We do not ascribe any significance to the Langmuir–Freundlich parameters beyond that they provide purely empirical relationships between the linear extension of *c* and CO_2_ pressure.

## Photomicroscopy

**Fig. 5:**
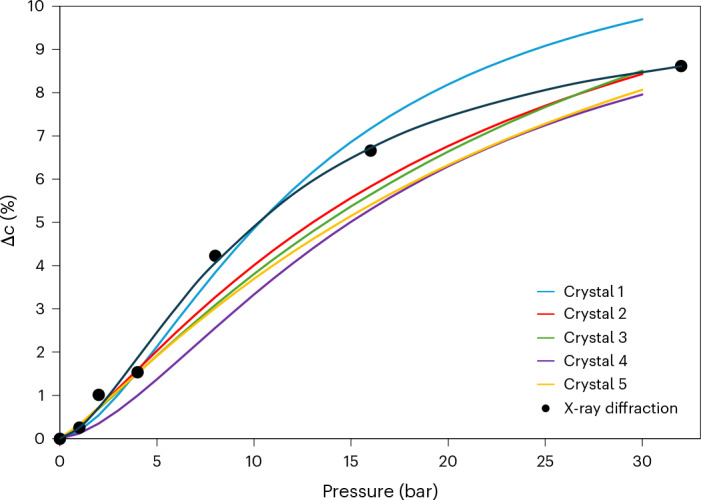
Elongation of T1 along [001] with increasing pressure. Langmuir–Freundlich models of the percentage elongation of the needle axes of five different crystals of **T1** in the range of 0–30 bar as measured from the in situ photomicrographs. The black circles represent the relative elongation of the crystallographic *c* axis of the sixth crystal in the range of 0–32 bar, with the corresponding Langmuir–Freundlich model shown as a solid black line. Source data

It is reasonable to assume that a pronounced change in one or more of the unit-cell dimensions of a crystal might be observable at the micro- or macroscopic level^26–29^. To this end, we constructed an apparatus consisting of a microscope-mounted pressure cell attached to an electronic pressure valve (Supplementary Text 12). Bespoke software was used to automatically record the time-lapse photomicrographs and controlling the pressure within the cell according to a predefined pressure–time ramp rate. A series of micrographs was recorded for several crystals of **T1** exposed to CO_2_ in the pressure range 0 → 32 → 0 bar and at a rate of 0.2 bar min^−1^ (Supplementary Videos 8–12). Indeed, the micrograph recorded at 32 bar of CO_2_ shows elongation of the needle [001] axis of the crystal consistent with stretching of the crystallographic *c* axis for **T1**_**C32**_ (Fig. 4b). Moreover, the measurement of the lengths of several crystals at various CO_2_ pressures confirmed our hypothesis that the same Langmuir–Freundlich trend in gas-induced elongation experienced by the crystallographic *c* axis can be observed at the macroscopic scale (Fig. 5). Indeed, the photomicrographs show that the needle axis expands and contracts with increasing and decreasing pressure, respectively, with little hysteresis displayed over a relatively wide pressure range (Supplementary Figs. 17, 18, 20 and 21), and that the response of a crystal is repeatable over multiple pressure cycles (Supplementary Figs. 19 and 22) with its single-crystal integrity maintained. We also established that the crystals become friable at approximately 45 bar of CO_2_ pressure (Supplementary Fig. 23 and Supplementary Video 11), indicating that the maintenance of crystal singularity is subject to lattice distortion limits.

A limited set of experiments, similar to those described above for CO_2_, was carried out with CH_4_ gas to investigate the effect of a different guest on **T1** crystals (Supplementary Text 14). Elongation of the crystallographic *c* axis in the pressure range of 0–60 bar of CH_4_ was only approximately half that observed for the range of 0–32 bar of CO_2_, despite indications (cavity volumes and electron counts) that the 0D pockets are fully occupied by CH_4_ at 60 bar. This result can be rationalized based on the smaller molecular volume and approximately spherical shape of CH_4_ compared with CO_2_.

## Discussion

Our in situ crystallographic studies indicate that the gas molecules become lodged within the 0D cavities of the host molecules despite the absence of permanent pathways to these sites. Indeed, such transient porosity was first recognized more than two decades ago for the single-crystal-to-single-crystal inclusion of vinyl bromide into an apohost form of *p*-*tert*-butylcalix[4]arene^30^ and, subsequently, reaffirmed for the inclusion of CO_2_ and other gases into the same host system^16^. Although the overall packing arrangement of **T1** does not change during gas loading (that is, no phase transition occurs), the host molecules undergo subtle adjustment of their positions and orientations to accommodate the guests within their 0D pockets, accompanied by a concomitant swelling of the 1D channels. These molecular rearrangements cause a Langmuirian elongation of the crystallographic *c* axis, which manifests as comparable changes in the macroscopic dimensions of the crystal, as confirmed by in situ photomicroscopy (Supplementary Videos 8–12). Thus, the expansion of the channel volume is a direct result of the accommodation of gas molecules in the adjacent 0D voids of **T1**_**G***x*_ rather than the gas loading of the channel. These results are a proof-of-concept demonstration of control over the dimensions of a crystalline material using gas pressure as a stimulus, although in an extreme environment. Similar control has been reported extensively for other extreme environments, involving concepts such as thermal expansion and hydrostatic compressibility (for which the coefficients are given as *α* and *β*, respectively). Indeed, it is possible to borrow further from these fields by quantifying expansion along *c* using the equation:

$${\delta }_{{\rm{c}}}=\frac{1}{L}{\left(\frac{\partial L}{\partial P}\right)}_{{\rm{T}}},$$

where we define *δ*_c_ as the sorption-induced ‘linear swelling coefficient’, *L* is the length of *c* at 0 bar and ${\left(\frac{\partial L}{\partial P}\right)}_{{\rm{T}}}$ represents the pressure-dependent elongation of *c* at a constant temperature; the linear swelling coefficient *δ*_c_ = 2.69 × 10^−8^ Pa^−1^ (that is, ~27 GPa^−1^) for **T1**_**C32**_. We have also demonstrated that our data for swelling along *c* (both crystallographically and macroscopically) can be modelled using the Langmuir–Freundlich equation, thereby providing an empirical relationship between gas pressure and length of the crystallographic *c* axis. We have demonstrated that a porous material with flexible pockets that are initially too small to accommodate gas molecules can inflate substantially as gas molecules accumulate in these spaces under the influence of increasing pressure. These results add an additional stimulus to the growing arsenal of stimuli that can be used to control the dimensions of crystalline materials, and may have implications for diverse applications that include sensors and actuators, as well as those requiring optical components with tunable refractive indices.

## Methods

### Materials

All commercially available reagents were obtained from commercial suppliers and used in reactions without further purification, unless otherwise specified. The ^1^H and ^13^C nuclear magnetic resonance (NMR) spectra were recorded on a Bruker 300-MHz or Bruker 400-MHz spectrometer at the ambient temperature. The NMR spectra are reported in parts per million (ppm) downfield of the tetramethylsilane signal and were measured relative to the residual signals for CDCl_3_ (7.27 and 77.0 ppm, respectively, for ^1^H and ^13^C NMR). The ^13^C NMR spectra were obtained with ^1^H decoupling. Mass spectra were recorded on an AB Sciex TripleTOF 5600+ system. Melting points were measured using open glass capillaries in a Büchi Melting Point B-545 apparatus. The infrared spectra were measured using a Thermo Scientific Nicolet iS50 FTIR spectrometer.

### Single-crystal X-ray diffraction

Data were recorded using a Bruker D8 Venture equipped with a PHOTON II CPAD detector and an Oxford Cryosystems Cryostream 800Plus cryostat. Mo Kα X-rays (*λ* = 0.71073 Å) were generated using a multilayer Incoatec microfocus (IµS) source. A crystal-to-detector distance of 37 mm was used for all experiments. Data reduction was carried out using the Bruker SAINT^31^ software; absorption and other corrections were made using SADABS^32^ as implemented in the Bruker APEX 3 software package. Crystal structures were solved either using SHELXD^33^ or SHELXT^34^ via the X-Seed^35,36^ graphical user interface. Non-hydrogen atoms of the host were refined anisotropically using SHELXL^37^ using full-matrix least squares minimization. Host hydrogen atomic positions were calculated using riding models. The absolute structure of the investigated crystals was assumed from the known absolute configuration of (*R*,*R*)-1,2-diaminecyclohexane, which was used as a starting material in the syntheses.

### Variable-pressure in situ X-ray crystallography

Variable-pressure single-crystal X-ray diffraction experiments were carried out using an environmental gas cell developed in-house. Intensity data were recorded for samples exposed to CO_2_ in the pressure range of 0–32 bar. Since it is not possible to control the temperature of the entire gas cell using a conventional cryostat, the temperature of each data collection was taken to be the temperature of the diffractometer cabinet, which ranges between 26 and 27 °C.

In a typical experiment, a suitable crystal was attached to the end of a thin glass fibre by means of epoxy. The fibre was then inserted into a 0.3-mm Lindemann glass capillary, which was epoxy-sealed to a modified stainless steel barb fitting, which, in turn, was attached to a bespoke miniature valve. A high-pressure manifold equipped with calibrated test gauges was used to pressurize the gas cell before each diffraction experiment; in each case, the sample was allowed to equilibrate overnight at the desired pressure, with the temperature maintained at 27 °C. After equilibration, the gas-cell assembly was attached to a modified goniometer head, which was mounted onto the goniometer of the diffractometer.

### Crystallographic software

Probe-accessible extrinsic channel and intrinsic cavity volumes were calculated using MSRoll^38^ using a probe radius of 1.5 Å, and visualized using Mercury^39^. The Cambridge Structural Database^40^ (v. 5.46; database: November 2024) was accessed using Conquest^41^.

### Gas sorption analysis

Gravimetric sorption isotherms were recorded for an ~25-mg sample of **T1** crystals by means of an Intelligent Gravimetric Analyser (IGA-002) supplied by Hiden Isochema^42–46^. The instrument facilitates the precise measurement of mass change and the control of pressure and temperature. The pressure is monitored using a pressure transducer within a range of 0–20 bar and buoyancy effects are corrected by the control software. During each experiment, the temperature was maintained at 20 ± 0.05 °C using a Grant refrigerated recirculating bath. Data collection was controlled by real-time processing computer software that continually predicts the equilibrium pressure using least squares regression to extrapolate a value for the asymptote. A linear driving force relaxation model was used, with each point recorded once a 99% fit to the model was achieved. CO_2_ (99.995%) gas cylinders were purchased from Afrox. The sample was initially evacuated in situ for 2 h to ensure that it was fully activated.

### Variable-pressure in situ photomicroscopy

In situ microscopy experiments were carried out to record macroscopically visible changes in the dimensions of single crystals exposed to gas pressure at variable-pressure ramp rates. The apparatus required for these experiments (Supplementary Fig. 10) was developed in-house and consisted of a gas supply connected to a software-controlled electronic gas valve, which was, in turn, connected to a stainless steel pressure chamber equipped with 6-mm-thick quartz windows. The pressure chamber is attached to the sample stage of a microscope fitted with a USB camera. Experiments were controlled and monitored using bespoke software (Pressure_Valve, developed by L.J.B.). For the current study, photomicrographs were recorded at 30-s intervals and exposing crystals to gas at a ramp rate of 0.2 bar min^−1^. In a typical experiment, the pressure was increased from 0 bar to a selected maximum pressure, followed by a decrease in pressure back to 0 bar.

## Online content

Any methods, additional references, Nature Portfolio reporting summaries, source data, extended data, supplementary information, acknowledgements, peer review information; details of author contributions and competing interests; and statements of data and code availability are available at 10.1038/s41563-025-02393-6.

## Supplementary information

Supplementary Information Supplementary Texts 1–14, Figs. 1–27, Tables 1–8, captions for Supplementary Videos 1–13 and references.

Supplementary Video 1 Projections of an entire unit cell of the structure series **T1**_**C*x***_ along [001]. Probe-accessible space is shown as yellow surfaces (probe radius 1.5 Å, grid spacing 0.2 Å). The sequence shows enlargement of the 0D voids of the time-averaged crystal structures with increasing CO_2_ pressure. Images were created using Mercury.

Supplementary Video 2 Alternating spacefilling diagrams of **T1**_**0**_ and **T1**_**C32**_ projected along [001]. Six molecules of **T1** are shown, forming a ring that stacks along [001] to create 1 nm wide 1D channels. The video is best played with the repeat setting turned on and contrasts the orientations of the molecules at the two pressure extremes for which structural data are available. Images were created using X-Seed and POV-Ray.

Supplementary Video 3 Spacefilling diagrams of **T1**_**C*x***_ projected along [001]. Six molecules of **T1** are shown, forming a ring that stacks along [001] to create 1 nm wide 1D channels. The video shows the progression of the orientations of the molecules as the gas pressure is increased from 0 to 32 bar. Images were created using X-Seed and POV-Ray.

Supplementary Video 4 Projections of six successive unit cells of the structure series **T1**_**C*x***_ along [010]. Probe-accessible space is shown as yellow surfaces (probe radius 1.5 Å, grid spacing 0.2 Å). The sequence shows enlargement of the 1D channels in the time-averaged crystal structures as the **T1** molecules move apart to create space for the gas molecules within the 0D voids (voids omitted for clarity). Images were created using Mercury.

Supplementary Video 5 Alternating capped-stick diagrams showing the formation of two symmetry-independent 0D voids in **T1**_**0**_ and **T1**_**C32**_. Each void primarily consists of the intrinsic cavity of a molecule of **T1** and is capped by cyclohexane moieties of neighbouring host molecules. Crystallographically independent molecules are coloured blue and green. Probe-accessible space (blue surfaces) was calculated using MSRoll *via* the X-Seed interface (probe radius 1.5 Å). The alternating images show overall enlargement of the two distinct cavities between 0 and 32 bar of CO_2_ pressure. Images were created using X-Seed and the video is best played with the repeat setting turned on.

Supplementary Video 6 Packing of a simplified model (see Supplementary Fig. 9) of **T1** in the structures **T1**_**C*x***_, viewed perpendicular to [001]. The triangles represent hexameric assemblies of **T1** molecules and five successive assemblies are shown stacked along [001]. Red and grey triangles distinguish between crystallographically-independent molecules. The sequence shows how the molecules tilt slightly relative to [001] with increasing pressure, thus causing elongation of the periodicity along the crystallographic *c* axis. Images were created using X-Seed.

Supplementary Video 7 Video sequence showing structural adjustment of **T1**_**C*x***_ to increasing CO_2_ pressure (left) and corresponding photomicrographs (right) of the crystal at the same pressures.

Supplementary Video 8 Time-lapse sequence showing Crystal 1 responding to variable CO_2_ gas pressure. The video shows one cycle of ramping the pressure at a rate of 0.2 bar min^−1^ from 0 to 32 bar, and then back to 0 bar. The dashed vertical line on the left indicates where the crystal is attached to a glass fibre and the dashed vertical line on the right facilitates observation, relative to **T1**_**0**_, of gas-induced elongation along [001].

Supplementary Video 9 Time-lapse sequence showing Crystal 2 responding to variable CO_2_ gas pressure. The video shows one cycle of ramping the pressure at a rate of 0.2 bar min^−1^ from 0 to 30 bar, and then back to 0 bar.

Supplementary Video 10 Time-lapse sequence showing Crystal 3 responding to variable CO_2_ gas pressure. The video shows three cycles of ramping the pressure at a rate of 0.2 bar min^−1^ from 0 to 30 bar, and then back to 0 bar.

Supplementary Video 11 Time-lapse sequence showing Crystal 4 responding to variable CO_2_ gas pressure. The video shows one cycle of ramping the pressure at a rate of 0.2 bar min^−1^ from 0 to 50 bar, and then back to 0 bar.

Supplementary Video 12 Time-lapse sequence showing Crystal 5 responding to variable CO_2_ gas pressure. The video shows one cycle of ramping the pressure at a rate of 0.2 bar min^−1^ from 0 to 40 bar, and then back to 0 bar.

Supplementary Video 13 Time-lapse sequence showing Crystal 6 responding to variable CH_4_ gas pressure. The video shows one cycle of ramping the pressure at a rate of 0.2 bar min^−1^ from 0 to 40 bar, and then back to 0 bar.

Supplementary Data 1 (CCDC 2404629) Crystal structure of **T1** at 0 bar.

Supplementary Data 2 (CCDC 2404633) Crystal structure of **T1** at 1 bar of CO_2_.

Supplementary Data 3 (CCDC 2404628) Crystal structure of **T1** at 2 bar of CO_2_.

Supplementary Data 4 (CCDC 2404632) Crystal structure of **T1** at 4 bar of CO_2_.

Supplementary Data 5 (CCDC 2404630) Crystal structure of **T1** at 8 bar of CO_2_.

Supplementary Data 6 (CCDC 2404631) Crystal structure of **T1** at 16 bar of CO_2_.

Supplementary Data 7 (CCDC 2404634) Crystal structure of **T1** at 32 bar of CO_2_.

Supplementary Data 8 (CCDC 2404635) Crystal structure of **T1** at 0 bar.

Supplementary Data 9 (CCDC 2404640) Crystal structure of **T1** at 10 bar of CH_4_.

Supplementary Data 10 (CCDC 2404636) Crystal structure of **T1** at 20 bar of CH_4_.

Supplementary Data 11 (CCDC 2404638) Crystal structure of **T1** at 30 bar of CH_4_.

Supplementary Data 12 (CCDC 2404641) Crystal structure of **T1** at 40 bar of CH_4_.

Supplementary Data 13 (CCDC 2404639) Crystal structure of **T1** at 50 bar of CH_4_.

Supplementary Data 14 (CCDC 2404637) Crystal structure of **T1** at 60 bar of CH_4_.

## Source data

Source Data Fig. 2 CO_2_ and (CH_4_) gas sorption data and Langmuir–Freundlich fit.

Source Data Fig. 5 Langmuir–Freundlich fit data for percentage extension of the crystallographic *c* axis for multiple crystals.

## Data Availability

The main data supporting the findings of this study are available within the article and its Supplementary Information. Crystallographic data are tabulated in Supplementary Information and archived at the Cambridge Crystallographic Data Centre (CCDC) under reference numbers CCDC 2404628 to 2404641. Source data are provided with this paper.
