# Supplementary material for: Inflatable porous organic crystals
Source: Nat Mater. 2025 Nov 3;25(3):481–6. doi: 10.1038/s41563-025-02393-6 (PMC12960203; doi:10.1038/s41563-025-02393-6)
Supplement: Supplementary file 1 — Supplementary Texts 1–14, Figs. 1–27, Tables 1–8, captions for Supplementary Videos 1–13 and references. [file 41563_2025_2393_MOESM1_ESM.pdf]

nature materials

Supplementary information https://doi.org/10.1038/s41563-025-02393-6

Inflatable porous organic crystals

In the format provided by the authors and unedited

Contents

Supplementary Text 2
Supplementary Text 1. Synthetic Procedures 2
Supplementary Text 2. Crystallization 3
Supplementary Text 3. *In silico* mapping of probe-accessible space 3
Supplementary Text 3.1. Mercury 3
Supplementary Text 3.2. MSRoll 3
Supplementary Text 4. Formation of 0D voids in T1 crystals 5
Supplementary Text 5. Van der Waals radius of CO_2 6
Supplementary Text 6. Gas Sorption Analysis 7
Supplementary Text 6.1. Experimental 7
Supplementary Text 6.2. Data fitting 8
Supplementary Text 7. Variable-pressure *in situ* X-ray crystallography 10
Supplementary Text 7.1. Data collection, structure solution, and refinement 10
Supplementary Text 7.2. Pressure-induced deformation 13
Supplementary Text 7.3. Strain tensor calculation 13
Supplementary Text 8. Literature indications of crystal inflation 15
Supplementary Text 9. Visualizing molecular-level structural changes with gas pressure 33
Supplementary Text 10. Comment on electron counts based on difference electron density maps 33
Supplementary Text 11. Fitting the pressure-dependent change in *c* using sorption models 34
Supplementary Text 12. Variable-pressure *in situ* photomicroscopy 37
Supplementary Text 12.1. Sample chamber 38
Supplementary Text 12.2. Software control 39
Supplementary Text 12.3. Design considerations 40
Supplementary Text 12.4. Experimental procedure 41
Supplementary Text 12.5. Sources of commercially available components 42
Supplementary Text 13. Expansion and contraction of crystals due to changes in gas pressure 42
Supplementary Text 13.1. Crystal 1 43
Supplementary Text 13.2. Crystal 2 44
Supplementary Text 13.3. Crystal 3 46
Supplementary Text 13.4. Crystal 4 47
Supplementary Text 13.5. Crystal 5 48
Supplementary Text 13.6. Comparison between different crystals 49
Supplementary Text 14. Methane 50
Supplementary Text 14.1. Gas sorption analysis 50
Supplementary Text 14.2. Van der Waals radius of CH_4. 51
Supplementary Text 14.3. Variable-pressure *in situ* X-ray crystallography 52
Supplementary Video Captions 56
References 58

Supplementary Text

Supplementary Text 1. Synthetic Procedures

All reagents and solvents, except for 2,5-dihydroxyterephthalaldehyde, were purchased from commercial sources and used as received without further purification, unless otherwise indicated. Trianglimine **T1** was synthesized as outlined in Supplementary Scheme 1. All spectral data are consistent with literature values[47–49].

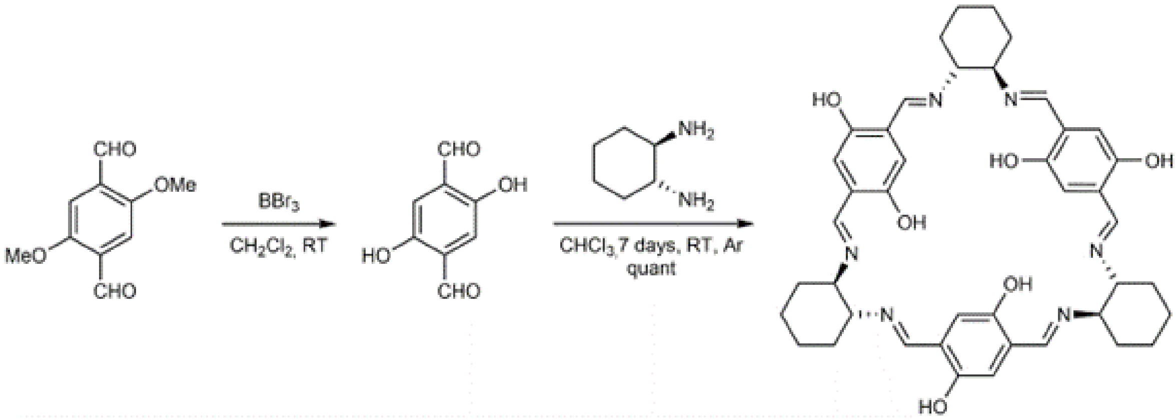

Supplementary Scheme 1. Synthesis of 13,27,42,44,45,47-hexahydroxy-3,10,17,24,31,38-hexaazaheptacyclo[38.2.2.212,15.226,29.04,9.018,23.032,37]octatetraconta-1(42),2,10,12,14,16,24,26,28,30,38,40,43, 45,47-pentadecaene (**T1**).

2,5-Dihydroxyterephthalaldehyde was obtained from 2,5-dimethoxyterephthalaldehyde according to a previously published procedure[47].

m.p. 169 – 169.5 °C;
^{1}H NMR (300 MHz, $CDCl_3$): δ = 10.23 (s, 2H), 9.96 (s, 2H), 7.24 (s, 2H);
^{13}C NMR (75.5 MHz, $CDCl_3$): δ = 196.42, 153.26, 125.19, 121.61;
IR (ATR): ṽ = 3487, 3264, 3053, 2890, 1663, 1475, 1459, 1277, 1122, 888, 832, 792, 665, 507 cm^{-1}.

T1 was obtained by a slight modification of a previously published procedure[48]. A solution of *trans*-(1*R*,2*R*)-diaminocyclohexane (56 mg, 0.5 mmol), 2,5-dihydroxyterephthalaldehyde (81.5 mg, 0.5 mmol) and $CHCl_3$ (25 mL) was stirred under an argon atmosphere at room temperature for 7 days. The mixture was then evaporated to dryness providing **T1** in quantitative yield. The product crystallized from a solution of ethanol as a yellow-orange solid.

m.p. decomposed above 300 °C;
^{1}H NMR (400 MHz, $CDCl_3$): *δ*=1.45-1.86 (m, 4H), 3.30 (m, 1H), 6.68 (s, 1H), 8.16 (s, 1H), 12.25 (s, 1H);
^{13}C NMR (400 MHz, $CDCl_3$): *δ*= 24.17, 32.95, 73.82, 118.37, 121.00, 152.49, 163.92;
MS (HR ESI-TOF^{+}): m/z found 733.3709 $[M+H]^{+}$, calcd $C_{42}H_{49}N_6O_6$ 733.3714;
$[\alpha]^{20}_{D}$ -407.1 (c = 1, $CHCl_3$);
IR (ATR): $\tilde{\nu}$ = 810.81, 854.50, 1040.78, 1097.74, 1157.78, 1215.59, 1309.96, 1362.40, 1448.25, 1510.23, 1622.36, 2653.40, 2700.34, 2858.07, 2928.19 cm^{-1}.

Supplementary Text 2. Crystallization

T1 (*ca*. 15 mg) was dissolved in a 1:1 mixture of absolute ethanol (1 mL) and dichloromethane (1 mL). The solution thus obtained was slowly evaporated at room temperature and relative humidity not exceeding 40%. **T1** initially formed a gel, which later liquified and deposited trigonal prismatic crystals (Supplementary Scheme 2).

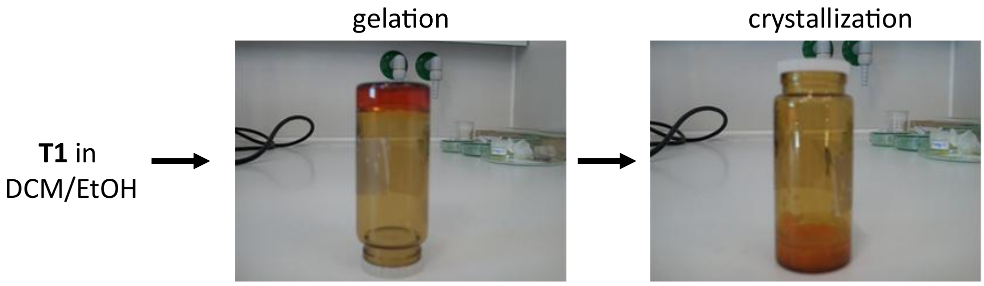

Supplementary Scheme 2. Crystallization of **T1**.

Supplementary Text 3. *In silico* mapping of probe-accessible space

Preliminary *in situ* evaluations of probe-accessible space were undertaken using crystallographic data previously published by our group[50] (CSD REFCODE = BIJLIT01, T = 298 K, P = 1 atm).

Supplementary Text 3.1. Mercury

The program Mercury[51] was used to calculate and visualize the contact surface as defined by a spherical probe of radius 1.5 Å and employing a grid spacing of 0.2 Å. Mercury reports the total probe-accessible volume per unit cell but does not enumerate contributions from different regions of non-contiguous space. Thus, the calculated probe-accessible volume of 3,756 Å^3 for the structure BIJLIT01 (Supplementary Fig. 1a) includes the volumes of three channels per unit cell, as well as those for 18 0D voids. After blocking the voids by virtually inserting lone carbon atoms at the **T1** centroids, the total probe-accessible volume per unit cell of 3,095 Å^3 (Supplementary Fig. 1b) represents three channels per unit cell, and hence 1,032.7 Å^3 per channel per unit cell (i.e. 1,032 Å^3 of channel volume per every 6 molecules of **T1**). The difference (3,756 Å^3 – 3,095 Å^3 = 661 Å^3) represents the total volume of the 18 blocked voids per unit cell, implying that the average void volume is 37 Å^3. However, it should be noted that Mercury uses a grid search algorithm that slightly underestimates void volumes. For smaller grids, the calculated free volumes become more accurate, but the calculations take much longer to perform. For example, using a grid spacing of 0.1 Å, the calculated volumes are 3,803 and 3,122 Å^3 for the blocked and unblocked structures, respectively, yielding average cavity volumes of 38 Å^3.

Supplementary Text 3.2. MSRoll

The program MSRoll[52] was used *via* the X-Seed[53,54] interface to calculate the volumes of the discrete cavities (Supplementary Fig. 2). Instead of using a grid search algorithm MSRoll computes a piecewise

quartic molecular surface and yields volumes that Mercury would compute using an infinitesimally small grid spacing and infinite time. Although not suitable for calculating the volumes of channels (i.e., the probe would "drop out" of the structure), MSRoll enumerates and accurately determines the volumes of discrete spaces.

Since they provide different types of information regarding probe-accessible space, Mercury and MSRoll can be used as complementary tools. It should be noted that neither of the programs considers the influence of thermal motion or structural disorder on calculations of free volumes, and results should always be treated with caution.

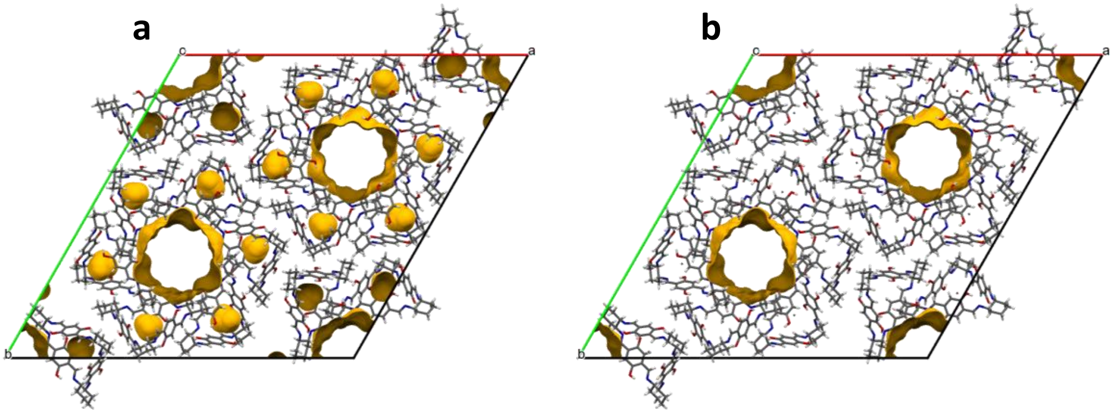

Supplementary Fig. 1. Mercury contact surface maps (probe radius 1.5 Å, grid spacing 0.2 Å) of (a) BIJLIT01 and (b) BIJLIT01 with the 0D cavities "blocked". In both cases the projections are along [001].

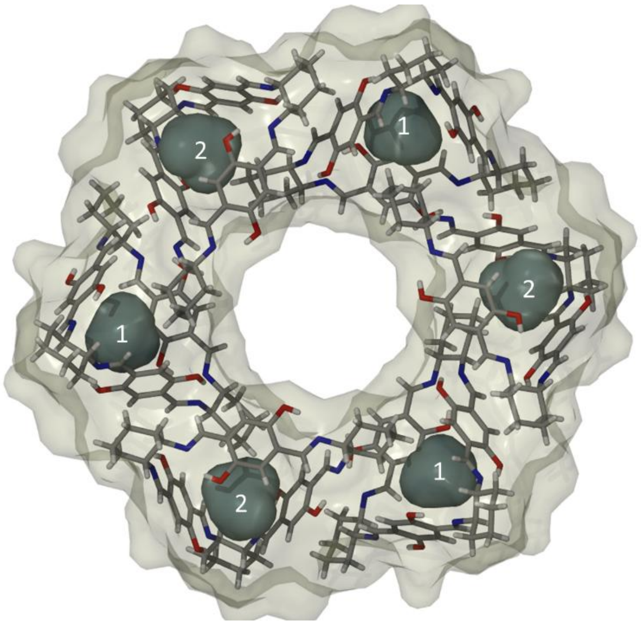

Supplementary Fig. 2. Perspective view along [001] of BIJLIT01 with MSRoll contact surfaces (probe radius 1.5 Å) shown. A circular arrangement of two crystallographically unique cavities 1 and 2 is shown in blue, with volumes of 37 and 45 $Å^3$, respectively.

Supplementary Text 4. Formation of 0D voids in T1 crystals

The previously published[50] crystal structure (CSD REFCODE BIJLIT01) shows that the asymmetric unit comprises two crystallographically unique molecules of **T1** situated close to a threefold rotation axis. Application of threefold rotational symmetry yields a hexameric ring (Supplementary Fig. 3a), which encircles a 1D channel propagating along [001]. With reference to Supplementary Fig. 3, alternating adjacent molecules, shown in blue and green, comprising the hexameric ring are crystallographically unique. The discussion that follows concerns the molecules shown with thick bonds. The 0D cavity associated with the aperture of the blue trianglimine molecule is shown as a blue surface. The cavity is capped on one side by a cyclohexane ring of the molecule depicted in green. At the opposite end, the cavity is capped by the cyclohexane moiety of a **T1** molecule in an adjacent hexameric ring (obscured), and which is related to the blue molecule by translation along *c*. Supplementary Fig. 3b shows a side view of two successive hexameric rings stacked along [001]. The two bold molecules shown in blue are related by translational symmetry along *c*. One of the cyclohexane moieties of the blue molecule on the right caps the intrinsic cavity of the blue molecule on the left.

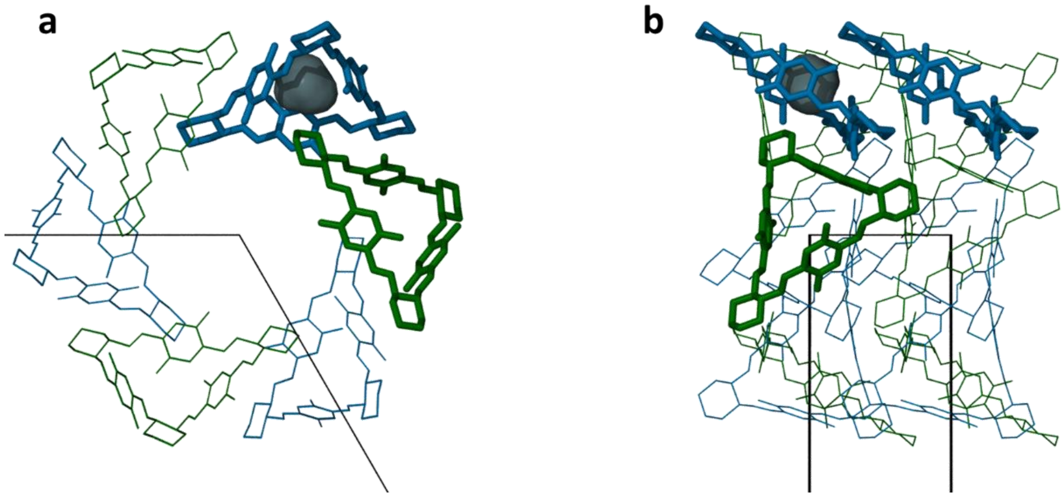

Supplementary Fig. 3. Perspective views of the crystal structure BIJLIT01, with **T1** molecules in capped-stick representation projected along (a) [001] and (b) [010]. Crystallographically independent molecules are shown in blue and green and hydrogen atoms have been omitted for clarity.

Although the two molecules of the asymmetric unit are crystallographically distinct, their structural roles in the formation of 0D voids are similar, as shown in Supplementary Fig. 4. The two unique cavities are referred to as Cavity 1 and Cavity 2 in this report.

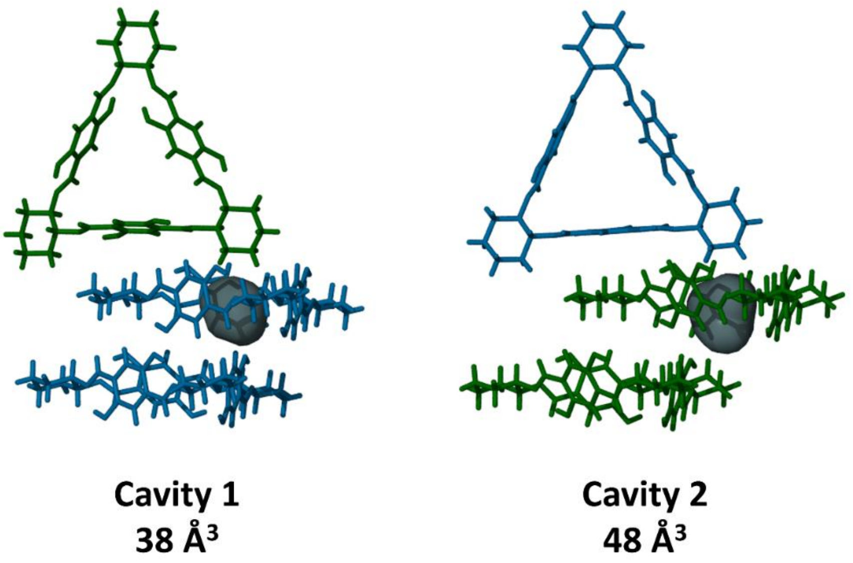

Supplementary Fig. 4. Perspective views showing the formation of two crystallographically unique hybrid intrinsic/extrinsic cavities in the crystal structure of **T1**. Trianglimine molecules are shown in capped-stick representation, and symmetry-independent molecules are colored blue and green.

Supplementary Text 5. Van der Waals radius of CO_2.

An idealized CO_2 molecule was constructed *in silico* using the bond lengths and angles shown in Supplementary Fig. 5. Van der Waals radii for the elements (C, 1.70 Å; O, 1.52 Å) were taken from Bondi[55] and bond lengths from https://cccbdb.nist.gov/exp2x.asp?casno=124389. The van der Waals volume of the molecule (i.e., the volume of the contact surface mapped by MSRoll (*65*) using a virtual probe of radius 0 Å) was determined[52].

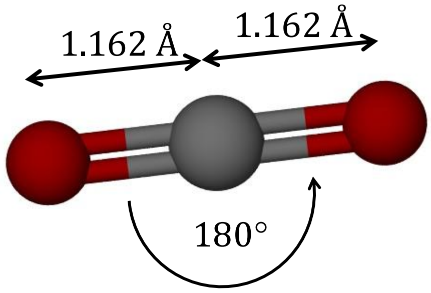

Supplementary Fig. 5. Model of CO_2 used to determine its van der Waals volume, with salient geometrical parameters shown.

Supplementary Text 6. Gas Sorption Analysis

Supplementary Text 6.1. Phase purity

To confirm phase purity, a subsample of **T1** crystals from the same batch as that used for gas sorption analysis was subjected to powder X-ray diffraction analysis. The crystals were gently milled using a mortar and pestle and distributed evenly onto a zero-background holder of a Bruker D2 PHASER diffractometer employing Ni-filtered Cu Kα radiation (λ = 1.5418 Å) operated at 30 kV and 10 mA. Intensity data were recorded at 30 °C using a Lynxeye 1D detector. The measured diffractogram was compared to that simulated from the single-crystal model with CSD REFCODE = BIJLIT01 using Mercury[51] (see Supplementary Fig. 6).

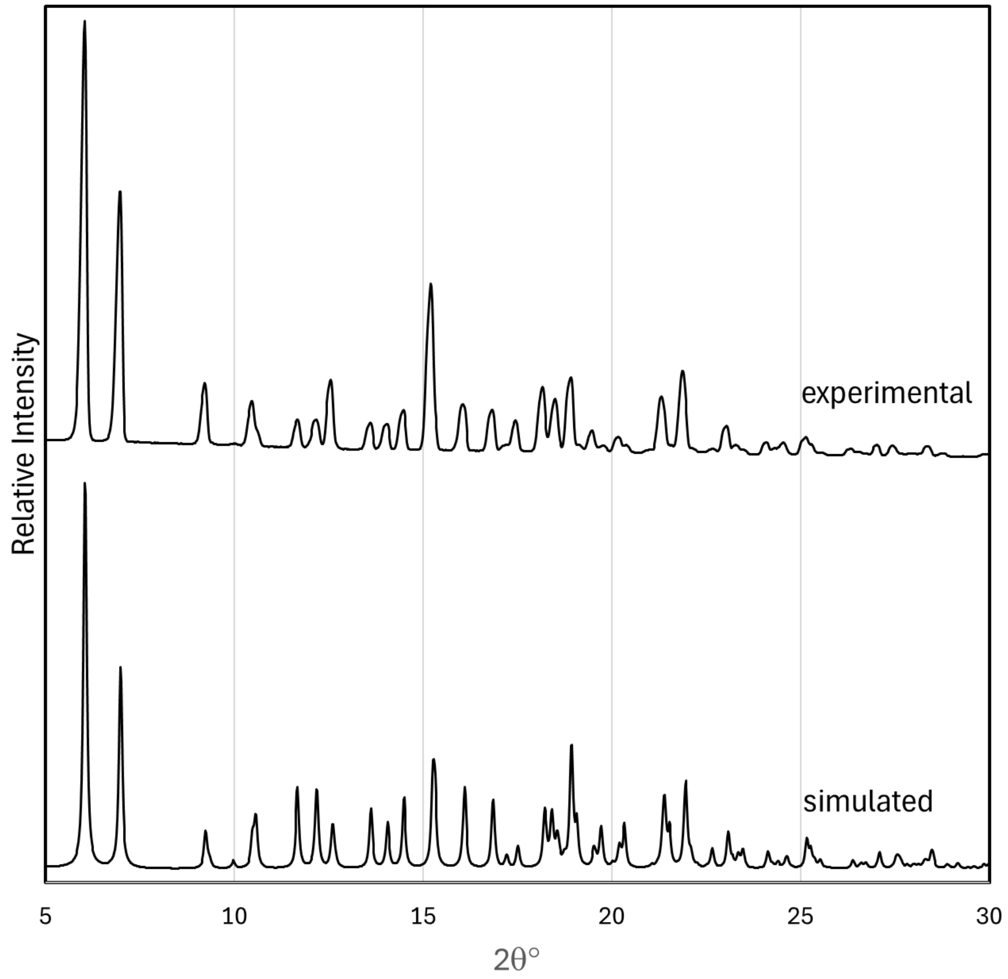

Supplementary Fig. 6. Experimental (top) and simulated (bottom) powder diffractograms of **T1**. Slight differences in intensities are attributed to preferred orientation.

Supplementary Text 6.1. Experimental

Gravimetric sorption isotherms (Supplementary Fig. 7) were recorded for a ca 25 mg sample of **T1** crystals by means of an Intelligent Gravimetric Analyser (IGA-002) supplied by Hiden Isochema, Warrington, UK[56–60]. The instrument facilitates precise measurement of mass change, and control of

pressure and temperature. The pressure is monitored using a pressure transducer with a range of 0-20 bar and buoyancy effects are corrected by the control software. During each experiment the temperature was maintained at 20 ± 0.05 °C using a Grant refrigerated recirculating bath. Data collection was controlled by real-time processing computer software that continually predicts the equilibrium pressure using least-squares regression to extrapolate a value for the asymptote. A Linear Driving Force (LDF) relaxation model was used, with each point recorded once a 99% fit to the model was achieved. Carbon dioxide (99.995%) gas cylinders were purchased from Afrox (LTD). The sample was initially evacuated *in situ* for 2 hours to ensure that it was fully activated.

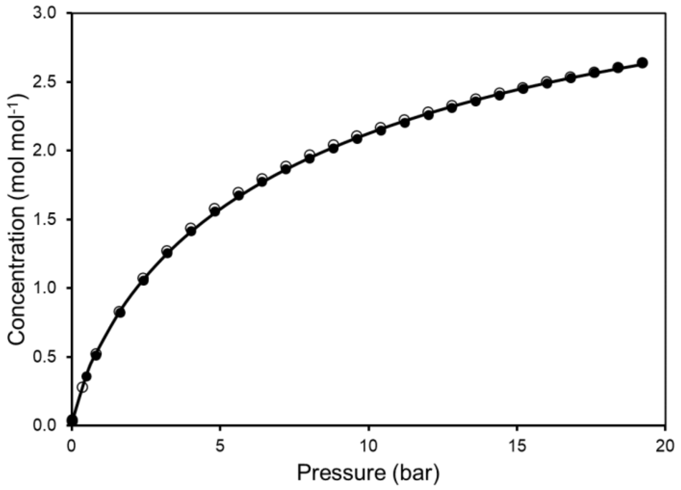

Supplementary Fig. 7. Absolute gas sorption isotherm for uptake and release of CO_2 by crystals of **T1**. Filled and open circles represent adsorption and desorption, respectively. Solid lines represent the best-fit Sips model for adsorption (see Supplementary Table 1).

Supplementary Text 6.2. Data fitting

The experimental adsorption data were modelled using the Sips[61] equation:

$$n = \frac{n_{max}(KP)^{\frac{1}{m}}}{1 + (KP)^{\frac{1}{m}}}$$

where n is the total number of moles sorbed at pressure P. The constants n_{max} (number of moles at maximum loading), K and $1/m$ were determined by implementing the Solver add-in of Microsoft Excel. The experimental and fitted data for adsorption of CO_2 by **T1** crystals are shown in Supplementary Table 1.

Supplementary Table 1. Experimental and Sips-fitted data for adsorption of CO_2 by crystals of **T1**.

P_{eq} / bar	n_{ads} / mol mol^{-1}	n_{fit} / mol mol^{-1}	R^2
0.0008	0.0361	0.0021	1.15E-03
0.0187	0.0478	0.0284	3.79E-04
0.4998	0.3594	0.3683	7.85E-05
0.8059	0.5121	0.5189	4.75E-05
1.6233	0.8238	0.8309	5.09E-05
2.4113	1.0578	1.0603	6.38E-06
3.2198	1.2548	1.2514	1.13E-05
4.0102	1.4184	1.4082	1.04E-04
4.8126	1.5578	1.5449	1.67E-04
5.6163	1.6754	1.6643	1.23E-04
6.4098	1.7770	1.7684	7.24E-05
7.2081	1.8661	1.8620	1.69E-05
8.0072	1.9459	1.9463	2.10E-07
8.8059	2.0184	2.0227	1.82E-05
9.6071	2.0860	2.0926	4.35E-05
10.4108	2.1477	2.1569	8.32E-05
11.2066	2.2057	2.2155	9.47E-05
12.0045	2.2596	2.2699	1.06E-04
12.8036	2.3115	2.3204	7.91E-05
13.6037	2.3590	2.3676	7.38E-05
14.4005	2.4056	2.4115	3.47E-05
15.2039	2.4504	2.4530	7.05E-06
16.0033	2.4909	2.4918	8.53E-07
16.8017	2.5303	2.5283	4.33E-06
17.6024	2.5684	2.5628	3.17E-05
18.4009	2.6057	2.5953	1.07E-04
19.2007	2.6415	2.6262	2.35E-04

R^2 is the square of the residual difference between n_{fit} and n_{ads}

ΣR^2	n_{max}	K	$1/m$
0.00313	3.98287	0.11807	0.80702

The adsorption data presented above extend to approximately 65% of the predicted n_{max}. While the Sips model was used to estimate the maximum adsorption capacity, the limited data range may introduce uncertainties in the extrapolated value. Specifically, the absence of data closer to the saturation region could affect the accuracy of the heterogeneity parameter (and equilibrium constant K, which are critical to determining n_{max}). We thus recommend interpreting the predicted n_{max} with caution.

Supplementary Text 7. Variable-pressure *in situ* X-ray crystallography

Supplementary Text 7.1. Data collection, structure solution, and refinement

Variable-pressure single-crystal X-ray diffraction (VP-SCD) experiments were carried out using an environmental gas cell developed in-house. Intensity data were recorded for samples exposed to CO_2 in the pressure range 0 to 32 bar. Since it is not possible to control the temperature of the entire gas cell using a conventional cryostat, the temperature of each data collection was taken to be the temperature of the diffractometer cabinet, which ranges between 26 and 27 °C.

In a typical experiment, a suitable crystal was attached to the end of a thin glass fiber by means of epoxy. The fiber was then inserted into a 0.3 mm Lindemann glass capillary, which was epoxy-sealed to a modified stainless steel barb fitting, which in turn was attached to bespoke miniature valve. A high-pressure manifold equipped with calibrated test gauges was used to pressurize the gas cell prior to each diffraction experiment; in each case the sample was allowed to equilibrate overnight at the desired pressure, with the temperature maintained at 27 °C. After equilibration, the gas cell assembly was attached to a modified goniometer head, which was mounted onto the goniometer of the diffractometer.

VP-SCD data were collected at 300 K on a Bruker D8 Venture diffractometer equipped with a PHOTON II CPAD detector. An X-ray beam of MoKα radiation (λ = 0.71073 Å) was generated by a multilayer Incoatec microfocus (IμS) source. Data were reduced using the Bruker software package SAINT [62]. Absorption and other corrections were implemented using SADABS [63] in the APEX 3 software package. Structures were solved using SHELXD (by importing the structure CSD REFCODE BIJLIT01 as a trial model), and by setting the following commands:

```
FIND 108 10
GROP 99 1.5 1.2 99
NTRY 40
PLOP 60 70 80 90 100 110 120 130 140 150
```

The structural models were completed by least-squares refinement on F^2 using SHELXL[64]. X−Seed[53,54] was used as a graphical interface for the SHELX programs, as well as for molecular graphics[65].

Selected crystallographic data for the VP-SCD structures are provided in Supplementary Table 2.

Supplementary Table 2. Crystal data and structure refinement parameters for structural analyses of **T1** crystals under vacuum and CO_2 pressure.

Identification code	$\mathbf{T1_0}$	$\mathbf{T1_{C01}}$	$\mathbf{T1_{C02}}$	$\mathbf{T1_{C04}}$
Gas pressure (bar)	0	1	2	4
Empirical formula	$C_{42}H_{48}N_6O_6$	$C_{42}H_{48}N_6O_6 \cdot 0.6(CO_2)$	$C_{42}H_{48}N_6O_6 \cdot 0.95(CO_2)$	$C_{42}H_{48}N_6O_6 \cdot 1.4(CO_2)$
Formula weight	732.86	759.27	774.67	794.48
Temperature (K)	300(2)	300(2)	300(2)	300(2)
Wavelength (Å)	0.71073	0.71073	0.71073	0.71073
Crystal system	trigonal	trigonal	trigonal	trigonal
Space group	*R*3:H	*R*3:H	*R*3:H	*R*3:H
Unit cell dimensions (Å)	$a = b = 50.581(4)$	$a = b = 50.777(3)$	$a = b = 50.829(3)$	$a = b = 50.762(3)$
	$c = 9.6557(9)$	$c = 9.6807(7)$	$c = 9.7537(8)$	$c = 9.8042(8)$
Volume ($Å^3$)	21394(4)	21616(3)	21823(3)	21879(3)
Z	18	18	18	18
Calculated density (g cm^{-3})	1.024	1.050	1.061	1.085
Absorption coefficient (mm^{-1})	0.069	0.073	0.074	0.077
F_{000}	7020	7258	7396	7574
Crystal size (mm^3)	0.194 × 0.211 × 0.320	0.194 × 0.211 × 0.320	0.194 × 0.211 × 0.320	0.194 × 0.211 × 0.320
θ range for data collection (°)	2.131 to 21.765	2.154 to 21.791	2.139 to 21.794	2.128 to 21.759
Miller index ranges	$-52 \le h \le 52, -52 \le k \le 52, -9 \le l \le 10$	$-52 \le h \le 52, -52 \le k \le 52, -9 \le l \le 10$	$-52 \le h \le 52, -52 \le k \le 52, -9 \le l \le 10$	$-52 \le h \le 52, -52 \le k \le 52, -9 \le l \le 10$
Reflections collected	61828	85932	64235	73291
Independent reflections	11196 [R_{int} = 0.1438]	11326 [R_{int} = 0.1298]	11434 [R_{int} = 0.1407]	11450 [R_{int} = 0.1316]
Completeness to θ_{max} (%)	0.997	0.996	0.995	0.998
Max. and min. transmission	0.858 and 1.000	0.866 and 1.000	0.816 and 1.000	0.879 and 1.000
Refinement method	Full-matrix least-squares on F^2	Full-matrix least-squares on F^2	Full-matrix least-squares on F^2	Full-matrix least-squares on F^2
Data / restraints / parameters	11196 / 1597 / 985	11326 / 1597 / 985	11434 / 1597 / 985	11450 / 1597 / 985
Goodness-of-fit on F^2	0.978	1.012	1.020	1.006
Final *R* indices [$I > 2\sigma(I)$]	$R1 = 0.0837$, $wR2 = 0.2001$	$R1 = 0.0899$, $wR2 = 0.2160$	$R1 = 0.1130$, $wR2 = 0.2677$	$R1 = 0.1153$, $wR2 = 0.2704$
R indices (all data)	$R1 = 0.1890$, $wR2 = 0.2727$	$R1 = 0.1770$, $wR2 = 0.2840$	$R1 = 0.2101$, $wR2 = 0.3447$	$R1 = 0.2106$, $wR2 = 0.3554$
Largest diff. peak and hole (e $Å^{-3}$)	0.319 and -0.351	0.433 and -0.411	0.752 and -0.458	0.770 and -0.455
Absolute structure parameter	0.5(10)	0.3(10)	0.3(10)	0.8(10)

Supplementary Table 2. (continued)

Identification code	**T1**$_{C08}$	**T1**$_{C16}$	**T1**$_{C32}$
Gas pressure (bar)	8	16	32
Empirical formula	$C_{42}H_{48}N_6O_6 \cdot 1.9(CO_2)$	$C_{42}H_{48}N_6O_6 \cdot 2.5(CO_2)$	$C_{42}H_{48}N_6O_6 \cdot 2.9(CO_2)$
Formula weight	816.48	842.89	860.49
Temperature (K)	300(2)	300(2)	300(2)
Wavelength (Å)	0.71073	0.71073	0.71073
Crystal system	trigonal	trigonal	trigonal
Space group	*R*3:H	*R*3:H	*R*3:H
Unit cell dimensions (Å)	*a* = *b* = 50.462(7)	*a* = *b* = 50.043(3)	*a* = *b* = 49.810(2)
	c = 10.0643(17)	*c* = 10.2991(8)	*c* = 10.4882(7)
Volume (Å^3)	22195(7)	22337(3)	22535(3)
Z	18	18	18
Calculated density (g cm^{-3})	1.100	1.128	1.141
Absorption coefficient (mm^{-1})	0.079	0.082	0.084
F_{000}	7772	8010	8168
Crystal size (mm^3)	0.194 × 0.211 × 0.320	0.194 × 0.211 × 0.320	0.194 × 0.211 × 0.320
θ range for data collection (°)	2.076 to 21.903	2.032 to 21.745	1.998 to 22.075
Miller index ranges	$-52 \le h \le 52$, $-52 \le k \le 52$, $-10 \le l \le 10$	$-52 \le h \le 52$, $-52 \le k \le 52$, $-10 \le l \le 10$	$-52 \le h \le 52$, $-51 \le k \le 52$, $-10 \le l \le 11$
Reflections collected	69536	72471	80673
Independent reflections	11702 [R_{int} = 0.1919]	11627 [R_{int} = 0.1359]	12124 [R_{int} = 0.1256]
Completeness to θ_{max} (%)	0.989	0.996	0.992
Max. and min. transmission	0.791 and 1.000	0.856 and 1.000	0.891 and 1.000
Refinement method	Full-matrix least-squares on F^2	Full-matrix least-squares on F^2	Full-matrix least-squares on F^2
Data / restraints / parameters	11702 / 1597 / 985	11627 / 1597 / 985	12124 / 1597 / 987
Goodness-of-fit on F^2	1.068	1.141	1.192
Final *R* indices [$I > 2\sigma(I)$]	*R*1 = 0.1437, *wR*2 = 0.3295	*R*1 = 0.1365, *wR*2 = 0.3249	*R*1 = 0.1370, *wR*2 = 0.3263
R indices (all data)	*R*1 = 0.2739, *wR*2 = 0.4185	*R*1 = 0.2308, *wR*2 = 0.3902	*R*1 = 0.2160, *wR*2 = 0.3854
Largest diff. peak and hole (e Å^{-3})	1.021 and -0.453	1.154 and -0.460	1.350 and -0.413
Absolute structure parameter	0.1(10)	0.1(10)	0.3(9)

Supplementary Text 7.2. Pressure-induced deformation

Salient structural parameters for **T1**$_{Cx}$ over the pressure range x = 0 to 32 bar are summarized in Supplementary Table 3 and displayed graphically in Supplementary Fig. 8.

Supplementary Table 3. Selected structural parameters for **T1**$_{Cx}$.

Pressure / bar	a / Å	c / Å	V_{cell} / Å^3	$V_{channel}$ / Å^3	%Δa	%Δc	%ΔV_{cell}	%Δ$V_{channel}$
0	50.581	9.656	21,394	996	0.00	0.00	0.00	0.00
1	50.777	9.681	21,616	990	0.39	0.26	1.04	-0.64
2	50.829	9.754	21,823	996	0.49	1.01	2.01	-0.03
4	50.762	9.804	21,879	999	0.36	1.54	2.27	0.30
8	50.462	10.064	22,195	1,051	-0.24	4.23	3.74	5.56
16	50.044	10.299	22,337	1,118	-1.06	6.66	4.41	12.22
32	49.810	10.488	22,535	1,192	-1.53	8.62	5.33	19.71

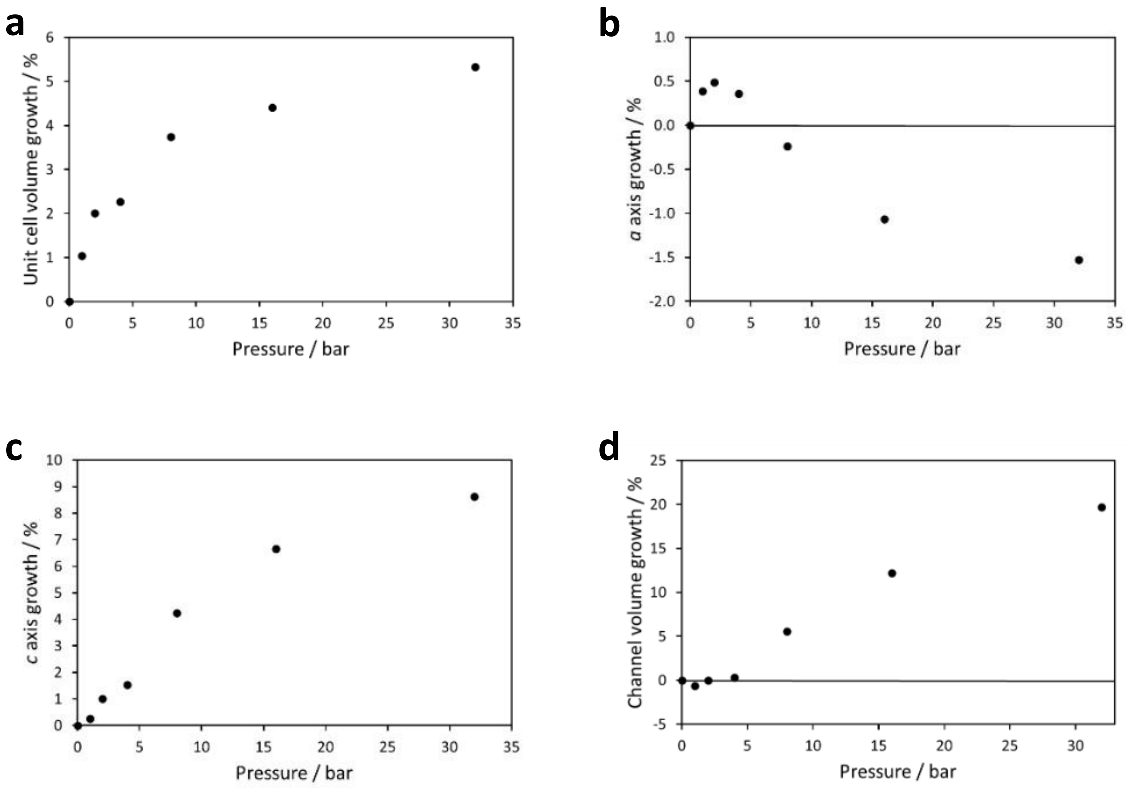

Supplementary Fig. 8. Plots of changes in selected lengths and volumes for the structures **T1**$_{Cx}$ with pressure. (a) Unit cell volume, (b) unit cell axis a, (c) unit cell axis c and (d) the volume of the channel that propagates along [001].

Supplementary Text 7.3. Strain tensor calculation

The program STRAIN (https://www.cryst.ehu.es/cryst/strain.html) was used to calculate the deformation of the unit cell of **T1**$_{C32}$ relative to that of **T1**$_0$.

Lattice parameters of cell 1 (undeformed, P = 0 bar):
a = 50.581 Å; b = 50.581 Å; c = 9.656 Å; α = 90°; β = 90°; γ = 120°

Lattice parameters of cell 2 (deformed, P = 32 bar):
a = 49.810 Å; b = 49.810 Å; c = 10.488 Å; α = 90°; β = 90°; γ = 120°

Metric tensor $\mathbf{M}_1$: $\begin{bmatrix} 2558.4 & -1279.2 & 0 \\ -1279.2 & 2558.4 & 0 \\ 0 & 0 & 93.2 \end{bmatrix}$

Metric tensor $\mathbf{M}_2$: $\begin{bmatrix} 2481.0 & -1240.5 & 0 \\ -1240.5 & 2481.0 & 0 \\ 0 & 0 & 110.0 \end{bmatrix}$

The standard root tensor **R** of metric tensor $\mathbf{M}=\mathbf{R}^T\mathbf{R}$ transforms fractional direct space coordinates **X**, into cartesian coordinates $\mathbf{X}_c$ such: $\mathbf{X}_c = \mathbf{R}\ \mathbf{X}$.

Standard root tensor $\mathbf{R}_1$: $\begin{bmatrix} 43.804 & 0 & 0 \\ -25.291 & 50.581 & 0 \\ 0 & 0 & 109.998 \end{bmatrix}$

Standard root tensor $\mathbf{R}_2$: $\begin{bmatrix} 43.137 & 0 & 0 \\ -24.905 & 49.810 & 0 \\ 0 & 0 & 10.488 \end{bmatrix}$

The linear Lagrangian strain tensor can be calculated according to the formula: $\mathbf{S} = 0.5\ (\mathbf{e} + \mathbf{e}^T)$, where, $\mathbf{e} = \mathbf{R}_2\mathbf{R}_1^{-1} - \mathbf{I}$ and, $\mathbf{R}_1$ and $\mathbf{R}_2$ are the standard root tensors of cells 1 and 2, respectively, and **I** is a 3×3 identity matrix.

Linear Lagrangian Strain Tensor (small deformation): $\begin{bmatrix} -0.015243 & 0 & 0 \\ 0 & -0.015243 & 0 \\ 0 & 0 & 0.086164 \end{bmatrix}$

Eigenvalues: –0.01524, –0.01524, 0.08616 (the third value describes elongation directly along [001])

The finite Lagrangian strain tensor can be calculated according to the formula: $\mathbf{S} = 0.5\ (\mathbf{e} + \mathbf{e}^T + \mathbf{e}^T\mathbf{e})$, where, $\mathbf{e} = \mathbf{R}_2\mathbf{R}_1^{-1} - \mathbf{I}$ and, $\mathbf{R}_1$ and $\mathbf{R}_2$ are the standard root tensors of cells 1 and 2, respectively, and **I** is a 3×3 identity matrix.

Finite Lagrangian Strain Tensor (finite deformation): $\begin{bmatrix} -0.015127 & 0 & 0 \\ 0 & -0.015127 & 0 \\ 0 & 0 & 0.089876 \end{bmatrix}$

Eigenvalues: –0.01513, –0.01513, 0.08988

The degree of lattice distortion is described here as the spontaneous strain (square root of the sum of squared eigenvalues of strain tensor) divided by 3

Degree of lattice distortion: 0.03080

Supplementary Text 8. Literature indications of crystal inflation

There are several examples in the literature of both dynamic and static inclusion of gases in crystals that result in changes in unit cell dimensions. Although it is reasonable to assume that such changes occurring at the unit cell level would also manifest macroscopically, none of the reported examples have been shown to undergo macroscopic crystal inflation or deflation due to the introduction or removal of gas. Supplementary Tables 4 and 5 list previously reported examples from an extensive but not exhaustive literature survey, showing that crystal inflation/deflation occurs at the unit cell level in solid-gas inclusion compounds involving several different classes of crystalline hosts and various gaseous guests. Dynamic inclusion (Supplementary Table 4) refers to a reversible process, whereby the gas can be introduced to and removed from a permanently or transiently porous crystalline host *a posteriori*. (i.e., after the host has been crystallized). Static inclusion (Supplementary Table 5) refers to an *a priori* process, where the host is crystallized in the presence of the gas, thereby permanently trapping the gas. Our survey demonstrates that the phenomenon of inflation/deflation is likely not only limited to **T1** crystals but may also be broadly applicable to many other host-guest systems. Although static inclusion does not demonstrate on-demand inflatability of crystals, we have included relevant examples to show that *a priori* gas inclusion is a related concept.

For each example listed in Supplementary Tables 4 and 5 a reference to the relevant article is provided, followed by the Cambridge Structural Database reference codes (CSD REFCODEs) for the associated crystal structures. These include the structure of the guest-free (activated, apohost) form of the host, as well as those of the gas-included forms. The type of host is listed, as well as the gaseous guest (— indicates no guest, or the apohost form). In some cases, guest inclusion is accompanied by a phase change (i.e., a change in the packing symmetry of the host framework), as indicated. Where possible, the gas pressure of the crystal environment during intensity data collection is provided (in some cases the gas pressures were not documented, or could not be determined accurately due to temperature changes either during gas-loading or data collection). We note that many of the apohost structures were documented as being "activated" and the articles did not specify whether the diffraction data were recorded under vacuum or other conditions (pressure indicated as "?").

In each case not involving an apohost phase change, the program STRAIN (https://www.cryst.ehu.es/cryst/strain.html) was used to calculate the deformation of the unit cell of the gas-loaded structure relative to that of the apohost form. STRAIN uses the two unit cells (before and after gas loading) to determine the Linear Lagrangian Strain Tensor (LLST) representing the deformation of the unit cell. The eigenvalues of the LLST indicate fractional deformation of the crystal along three principal (orthogonal) axes (i.e., the direction experiencing maximum magnitude of deformation, an orthogonal vector with the next highest deformation magnitude, and the third orthogonal vector). The tables report the three linear deformation eigenvalues e_1, e_2 and e_3 as percent deformation (i.e., the fractional deformation values calculated by STRAIN multiplied by 100). Positive and negative strain values indicate expansion and contraction, respectively, with gas loading. ΔVol indicates overall percentage of positive or negative volumetric deformation due to gas loading.

The final column categorizes the type of evidence for crystal inflation/deflation provided in each report (see note below Supplementary Table 5), with A-SCD being the most compelling. The remaining categories refer to various combinations of unknown gas pressure or inconsistent data collection

temperatures. In such cases dimensional changes may be due to gas pressure or thermal expansion effects. We have also included results obtained using X-ray powder diffraction; although several of these examples also involve gas-induced changes in unit cell dimensions, they do not represent reversible inflatability of single crystals. The survey was carried out using the Conquest program of the CSD software suite (March 2024 update)[66]. A query was generated by drawing the guest molecule of interest (gases such as CO_2, CH_4, N_2, etc.), results were inspected individually, and then the relevant article was consulted for further experimental details. Note: our survey does not include instances where the structure in the CSD does not specify gas/guest inclusion.

Supplementary Table 4. Reported examples of dynamic gas inclusion.

Reference	REFCODE	Host	Guest	Phase change	Pressure (bar)	Temp. (K)	Space group	e_1 / %	e_2 / %	e_3 / %	ΔVol / %	Category
67	BOGXIF02	MOF	—	—	?	195	*R*-3	—	—	—	—	B-SCD
	SUHPIV	MOF	CO_2	N	0.05–08	195	*R*-3	-0.127	-0.127	0.028	-0.222	B-SCD
	SUHPUH	MOF	CO_2	N	0.05–08	195	*R*-3	0.248	0.248	0.035	-0.456	B-SCD
	SUHQES	MOF	CO_2	N	0.05–08	195	*R*-3	-0.339	-0.339	0.085	-0.595	B-SCD
	SUHQUI	MOF	CO_2	N	0.05–08	195	*R*-3	-0.354	-0.354	0.085	-0.622	B-SCD
	SUHMIS	MOF	C_2H_2	N	0.05–08	195	*R*-3	-0.349	-0.349	0.155	-0.543	B-SCD
	SUHNAL	MOF	C_2H_2	N	0.05–08	195	*R*-3	0.603	0.603	0.465	-0.742	B-SCD
	SUHNIT	MOF	C_2H_2	N	0.05–08	195	*R*-3	-0.739	-0.739	0.606	-0.879	B-SCD
	SUHPAN	MOF	C_2H_2	N	0.05–08	195	*R*-3	-0.785	-0.785	0.648	-0.927	B-SCD
	BOGXIF01	MOF	—	—	?	123	*R*-3	—	—	—	—	B-SCD
	AJABOD	MOF	CO_2	N	10–20	123	*R*-3	-0.341	-0.341	0.098	-0.585	B-SCD
	AJABIX	MOF	C_2H_2	N	10–20	123	*R*-3	-0.813	-0.813	0.675	-0.953	B-SCD
	BOBTUI	MOF	N_2	N	?	93	*R*-3	-0.427	-0.427	0.316	-0.537	D-SCD
68	HAGSOY02	CP	—	—	?	93	*C*2/*c*	—	—	—	—	
	BELKEJ		CO_2	Y	1	93	*P*-1	n/a	n/a	n/a	5.561	B-SCD
69	XUVMOQ10	CP	—	—	?	90	*C*2/*c*	—	—	—	—	
	IKURIP01	CP	CO_2	Y	1	90	*P*-1	n/a	n/a	n/a	5.148	B-SCD
	HAGSOY07	CP	—	—	?	90	*C*2/*c*	—	—	—	—	
	BELKEJ01	CP	CO_2	Y	1	90	*P*-1	n/a	n/a	n/a	7.300	B-SCD
	DOQPAB02	CP	—	—	?	90	*P*-1	—	—	—	—	
	LUSVAX	CP	CO_2	N	1	90	*P*-1	n/a	n/a	n/a	9.867	B-SCD

	LUSVEB01	CP	—	—	?	90	*P*-1	—	—	—	—	
	LUSVOL	CP	CO_2	N	1	90	*P*-1	n/a	n/a	n/a	10.725	B-SCD
	LUSVUR01	CP	—	—	?	90	*P*-1	—	—	—	—	
	LUSWEC	CP	CO_2	N	1	90	*P*-1	0.743	0.045	-0.647	0.131	B-SCD
	LUSWIG	CP	CO_2	N*	17	90	*P*-1	n/a	n/a	n/a	5.971	B-SCD
	LUSWOM01	CP	—	—	?	90	*P*-1	—	—	—	—	
	LUSXAZ	CP	CO_2	N	1	90	*P*-1	3.480	0.308	2.278	1.480	B-SCD
70	KIXGUX	MOF	—	—	0	298	*Pnn*2	—	—	—	—	
	KIXHIM	MOF	CO_2	N	1	298	*Pnn*2	-0.027	0.265	-0.429	-0.190	A-SCD
	KIXHAE	MOF	CO_2	N	2.5	298	*Pnn*2	0.131	0.047	-0.065	-0.056	A-SCD
	KIXGEH	MOF	CO_2	N	5	298	*Pnn*2	0.352	0.526	-0.829	0.043	A-SCD
	KIXGAD	MOF	CO_2	N	10	298	*Pnn*2	0.515	0.640	-0.926	0.224	A-SCD
	KIXGIL	MOF	CO_2	N	10	225	*Pnn*2	0.670	0.988	-1.071	0.576	C-SCD
	KIWSUI	MOF	CO_2	N	10	250	*Pnn*2	0.587	0.819	-1.108	0.288	C-SCD
	KIXGOR	MOF	CO_2	N	10	273	*Pnn*2	0.604	0.777	-1.080	0.294	C-SCD
71	TACQUL01	MOF	—	—	?	195	*P*-1	—	—	—	—	
	LIYNOA	MOF	CO_2	N	1	195	*P*-1	-0.813	0.034	1.870	1.086	B-SCD
72	TOBJAZ	MOF	—	—	?	193	*Pm*-3*n*	—	—	—	—	
	TOBJIH	MOF	CO_2	N	?	193	*Pm*-3*n*	-0.079	-0.079	-0.079	-0.240	B-SCD
	TOBJED	MOF	SO_2	N	?	193	*Pm*-3*n*	-0.068	-0.068	-0.068	-0.209	B-SCD
73	BIFGAC	MOF	—	—	?	173	*Cmmm*	—	—	—	—	

	BIFFIJ01	MOF	—		?	193	*Cmmm*	n/a	n/a	n/a	0.000	
	BIFFUV	MOF	CO_2	Y	1	193	*P*-3	n/a	n/a	n/a	-0.137	C-SCD
	BIFFOP	MOF	C_2H_2	N	0.1	173	*Cmmm*	n/a	n/a	n/a	-0.368	A-SCD
	BIFFEF	MOF	C_2H_2	N	1	300	*Cmmm*	n/a	n/a	n/a	0.806	C-SCD
74	CEDKUS	MOF	—	—	?	373	$Pca2_1$	—	—	—	—	
	CEDLED	MOF	CO_2	Y	?	193	*Pcan*	9.945	1.315	6.190	18.286	D-SCD
75	VUWNIM	POM	—	—	?	100	$P2_1/c$ [*1]	—	—	—	—	
	DABXEM	POM	CO_2	N	?	100	$P2_1/c$ [*1]	0.212	0.125	-0.153	0.178	B-SCD
76	DOYCIG02	HOF	—	—	0	298	*C*2/*m*	—	—	—	—	
	DOXYUN	HOF	CO_2	N	2	298	*C*2/*m*	0.109	-0.012	-0.119	-0.003	A-SCD
	DOYDON	HOF	CO_2	N	5	298	*C*2/*m*	0.231	0.048	-0.161	0.132	A-SCD
	DOXYOH	HOF	CO_2	N	10	298	*C*2/*m*	0.231	0.065	-0.195	0.105	A-SCD
	DOYBIF	HOF	CO_2	N	20	298	*C*2/*m*	0.312	0.210	-0.208	0.330	A-SCD
	DOXZUO	HOF	CO_2	N	25	298	*C*2/*m*	0.326	0.208	-0.249	0.290	A-SCD
	DOXYIB	HOF	CO_2	N	30	298	*C*2/*m*	0.353	0.198	-0.305	0.260	A-SCD
	DOYCIG03	HOF	—	—	0	298	*C*2/*m*	—	—	—	—	A-SCD
	DOYCOM	HOF	Xe	N	2	298	*C*2/*m*	0.394	0.329	-0.613	0.114	A-SCD
	DOYBUR	HOF	Xe	N	5	298	*C*2/*m*	0.830	0.837	-0.334	1.348	A-SCD
	DOYDUT	HOF	Xe	N	10	298	*C*2/*m*	0.734	0.887	-0.478	1.153	A-SCD
	DOYDIH	HOF	Xe	N	15	298	*C*2/*m*	1.238	1.345	-0.042	2.565	A-SCD
	DOYBOL	HOF	Xe	N	20	298	*C*2/*m*	0.585	0.743	-0.692	0.638	A-SCD
	DOYDAZ	HOF	Xe	N	25	298	*C*2/*m*	0.762	0.850	-0.547	1.077	A-SCD
	DOYBAX	HOF	Xe	N	30	298	*C*2/*m*	0.789	0.969	-0.599	1.166	A-SCD
77	ELOSIK	MOF	—	—	0	292	*I*2/*a*	—	—	—	—	

	ELOTEH	MOF	CO_2	N	1.2	292	*I*2/*a*	0.022	0.549	-0.215	0.348	A-SCD
78	ASONOM01	POM	—	—	?	133	*P*2$_1$/*n*	—	—	—	—	
	FARQUM	POM	CO_2	N	1	133	*P*2$_1$/*n*	0.390	0.069	0.767	1.231	B-SCD
79	GIDCOO01	MOF	—	—	?	270	*I*4/*mmm*	—	—	—	—	
	GICFIK	MOF	CO_2	N	1	270	*I*4/*mmm*	-0.359	-0.359	-0.188	-0.897	A-SCD
	GICTAQ	MOF	CO_2	N	1	270	*I*4/*mmm*	-0.244	-0.244	-0.084	-0.567	A-SCD
	GICTEU	MOF	CO_2	N	1	270	*I*4/*mmm*	-0.459	-0.459	-0.288	-1.201	A-SCD
80	GUBJEV	PCP	—	—	?	183	*C*2/*c*	—	—	—	—	
	GUBJIZ	PCP	CO_2	N	1	183	*C*2/*c*	-1.558	2.081	-0.163	0.335	B-SCD
81	IDUTIN	MOF	—	—	?	110	*P*2$_1$/*n*	—	—	—	—	
	IDUTEJ	MOF	CO_2	N	?	110	*P*2$_1$/*c*	1.339	-2.159	0.264	-0.583	B-SCD
	IDUSIM02	MOF	C_2H_2	N	?	110	*P*2$_1$/*n*	-2.251	3.141	0.531	1.366	B-SCD
	IDUTAF	MOF	CH_4	N	?	85	*P*2$_1$/*n*	-0.171	0.197	-0.091	-0.060	D-SCD
82	XUVMOQ01	PCP	—	—	?	293	*C*2/*m*	—	—	—	—	
	XUVMOQ03	PCP	CO_2	N	1	293	*C*2/*m*	-1.458	0.180	-2.216	-3.456	B-SCD
	XUVMOQ02	PCP	—	—	?	93	*C*2/*c*	—	—	—	—	
	IKURIP	PCP	CO_2	Y	1	93	*P*-1	n/a	n/a	n/a	7.379	B-SCD
83	IVOLUC	MOF	—	—	?	110	*C*2/*c*	—	—	—	—	
	IVOLEM	MOF	CO_2	N	?	110	*C*2/*c*	-0.144	0.265	-0.105	0.014	B-SCD

	IVOLIQ	MOF	CO_2	N	?	110	*C*2/*c*	0.005	0.075	0.079	0.159	B-SCD
84	JETVOW	MOF	—	—	?	298	*P*3_12_1	—	—	—	—	
	JETVUC	MOF	CO_2	N	1	298	*P*3_12_1	-0.078	-0.078	0.384	0.230	A-SCD
85	KELCUA	PCP	—	—	?	298	*P*-1	—	—	—	—	
	KELDAH	PCP	CO_2	N	64	298	*P*-1	0.561	-0.108	-0.039	0.473	B-SCD
	KELCUA01	PCP	—	—	?	90	*P*-1	—	—	—	—	
	KELDEL	PCP	CO_2	Y	32	90	*P*-1	n/a	n/a	n/a	-44.560	B-SCD
86	KOMMIL	MOF	—	—	0 *2	298	*Fddd*	—	—	—	—	
	KOMLIK	MOF	CO_2	N	3	298	*Fddd*	0.464	-0.232	0.852	1.088	A-SCD
	KOMLOQ	MOF	CO_2	Y	10	298	*C*2/*c*	n/a	n/a	n/a	4.350	A-SCD
	KOMLUW	MOF	CO_2	Y	14	298	*C*2/*c*	n/a	n/a	n/a	4.626	A-SCD
	KOMMAD	MOF	CO_2	Y	18	298	*C*2/*c*	n/a	n/a	n/a	4.968	A-SCD
	KOMLIK01	MOF	CO_2	Y	22	298	*C*2/*c*	n/a	n/a	n/a	5.191	A-SCD
	KOMMOR01	MOF	CO_2	Y	30	298	*C*2/*c*	n/a	n/a	n/a	5.405	A-SCD
	KOMMOR	MOF	CO_2	Y	35	298	*C*2/*c*	n/a	n/a	n/a	5.424	A-SCD
87	LEWVAL01	MOF	—	—	?	298	*C*2/*c*	—	—	—	—	
	LAXWUG01	MOF	CO_2	N	20	298	*C*2/*c*	0.510	-0.465	0.271	0.326	A-SCD
88	LEZLUB	MOF	—	—	0	298	*P*2_1/*n*	—	—	—	—	
	LEZMOW	MOF	CO_2	N	1	298	*P*2_1/*n*	4.701	3.539	-1.656	6.671	A-SCD
	LEZMUC	MOF	CO_2	N	56	298	*P*2_1/*n*	7.048	-1.980	6.376	11.775	A-SCD

89	LIRROX	HOF	—	—	?	150	*P*-1	—	—	—	—	
	LIRRUD	HOF	C_2H_2	N	1	150	*P*-1	-1.759	-2.869	3.673	-0.987	B-SCD
	LIRSAK	HOF	CO_2	N	1	150	*P*-1	-0.241	-0.148	0.191	-0.203	B-SCD
90	Not found [*3]	MOF	—	—	0	298	$P2_1/c$	—	—	—	—	
	GIQPEF	MOF	CH_4	N	20	298	$P2_1/c$	-0.496	-0.132	1.647	1.014	A-SCD
	SEYQIZ	MOF	C_2H_6	N	6.3	298	$P2_1/c$	-0.632	-0.111	2.341	1.593	A-SCD
	SEYQUL	MOF	C_2H_6	Y	10	298	*P*-1	n/a	n/a	n/a	53.343	A-SCD
91	IRIRAC01	CC	—	—	?	90	*P*-3*c*1	—	—	—	—	
	GIXRIP	CC	Ar	N	44	90	*P*-3*c*1	0.433	0.433	-0.167	0.699	A-SCD
	GIXROV	CC	Xe	N	41	90	*P*-3*c*1	3.414	3.414	-1.150	5.714	A-SCD
	GIXRUB	CC	O_2	N	58	90	*P*-3*c*1	0.367	0.367	-0.168	0.564	A-SCD
	GIXSAI	CC	CH_4	N	48	90	*P*-3*c*1	0.245	0.250	-0.119	0.370	A-SCD
92	SIKLIK01	XBF	—	—	0	298	$Pna2_1$	—	—	—	—	
	SIKLUW	XBF	CO_2	N	20	296	$Pna2_1$	0.247	-0.292	0.996	0.949	A-SCD
	SIKLAC	XBF	CH_4	N	20	296	$Pna2_1$	0.247	0.408	0.294	0.952	A-SCD
	SIKKUV	XBF	C_2H_6	N	20	296	$Pna2_1$	0.804	6.513	-0.646	6.676	A-SCD
	SIKLOQ	XBF	C_3H_8	N	7	296	$Pna2_1$	0.613	7.005	0.121	7.791	A-SCD
	SIKMAD	XBF	C_4H_{10}	N	2	296	$Pna2_1$	0.758	7.865	-0.360	8.267	A-SCD
93	OFERUN06	MOF	—	—	0	298	*I*-43*m*	—	—	—	—	
	XIZCOA	MOF	CH_4	N	50	298	*I*-43*m*	-0.188	-0.188	-0.188	-0.550	A-SCD
	XIZCUG	MOF	C_2H_6	N	35	298	*I*-43*m*	-0.276	-0.276	-0.276	-0.814	A-SCD
	XIZDAN	MOF	C_3H_8	N	2	298	*I*-43*m*	-0.270	-0.270	-0.270	-0.805	A-SCD
	XIZDER	MOF	C_3H_8	N	4	298	*I*-43*m*	-0.220	-0.220	-0.220	-0.648	A-SCD
	XIZDIV	MOF	C_3H_8	N	8.7	298	*I*-43*m*	-0.194	-0.194	-0.194	-0.571	

94	OHEQUP07	MOF	—	—	0	298	*P*-1	—	—	—	—	
	OHEQUP06	MOF	CH_4	N	20	298	*P*-1	-1.033	-0.529	1.627	0.061	A-SCD
	AXOWIW	MOF	C_2H_6	Y	20	298	*P*-1	n/a	n/a	n/a	1.020	A-SCD
	AXOWES	MOF	C_3H_8	N	8	298	*P*-1	-0.221	-5.338	9.299	3.520	A-SCD
	AXOWUI	MOF	C_4H_{10}	N	2	298	*P*-1	-0.402	-3.295	9.778	6.065	A-SCD
95	IVAPAZ	MOF	—	N	?	120	*Pnna*	—	—	—	—	
	IVAPED	MOF	C_2H_6	N	?	120	*Pnna*	-0.487	-0.785	0.046	0.000	
	IVAPON	MOF	C_2H_6	N	?	120	*Pnna*	-0.509	-0.596	0.761	0.000	B-SCD
	IVAPIH	MOF	C_2H_4	N	?	120	*Pnna*	-0.506	-0.878	0.481	0.000	B-SCD
96	RIDJAT	MOF	—	—	0	120	*I*2/*a*	—	—	—	—	
	RIDHEV	MOF	C_2H_6	N	?	120	*I*2/*a*	0.149	0.167	-0.464	-0.150	B-SCD
	RIDHIZ	MOF	C_2H_4	N	?	120	*I*2/*a*	0.488	0.216	-0.447	0.252	B-SCD
	RIDKEY	MOF	—	N	0	120	*R*-3*c*	—	—	—	—	
	RIDHOF	MOF	C_2H_6	N	?	120	*R*-3*c*	0.004	0.004	-0.038	-0.033	B-SCD
	RIDHUL	MOF	C_2H_4	N	?	120	*R*-3*c*	-0.130	-0.130	0.010	-0.250	B-SCD
97	MAFMOY01	MOF	—	—	0	298	$P2_1/c$	—	—	—	—	
	DAFSEL	MOF	CO_2	N	2	298	$P2_1/c$	-4.581	6.648	1.835	3.632	A-SCD
	DAFSOV	MOF	CO_2	N	20	298	$P2_1/c$	-3.653	9.415	3.448	9.077	A-SCD
	DAFTEM	MOF	CO_2	N	20	298	$P2_1/c$	-1.938	2.990	8.125	9.161	A-PCD
	DAFTIQ	MOF	CO_2	Y	50	298	*P*1	n/a	n/a	n/a	n/a	A-PCD
98	MEBSAR	MOF	—	—	0	173	*P*2/*n*	—	—	—	—	
	MEBSEV	MOF	CO_2	Y	0.4	173	Article: *C*2/*c*<br>CSD: *I*2/*a*	n/a	n/a	n/a	17.377	B-SCD

99	ADABAK	MOF	—	—	0	296	*C*2/*c*	—	—	—	—	
	ADABEO	MOF	CO_2	N	?	200	*C*2/*c*	1.500	0.070	1.776	-0.233	D-PCD
	ADABIS	MOF	CO_2	N	?	296	*C*2/*c*	0.972	-0.228	-1.532	-0.804	B-PCD
	ADABAK01 [*4]	MOF	N_2	N	?	296	*C*2/*c*	-0.245	0.021	-0.408	-0.633	B-PCD
100	SATNOR02	MOF	—	—	?	10	*R*-3	—	—	—	—	
	COKMIA	MOF	CO_2	N	CO_2 [*5]	10	*R*-3	-0.036	-0.036	-0.131	-0.198	B-PCD
	COKPOJ	MOF	CO_2	N	CO_2 [*6]	10	*R*-3	-0.127	-0.127	-0.093	-0.346	B-PCD
	WOBHIF03	MOF	—	—	?	10	*R*-3	—	—	—	—	
	COKNAT	MOF	CO_2	N	CO_2 [*7]	10	*R*-3	-0.058	-0.058	0.038	-0.083	B-PCD
	COKPUP	MOF	CO_2	N	CO_2 [*8]	10	*R*-3	-0.305	-0.305	0.165	-0.447	B-PCD
	COKQOK	MOF	CO_2	N	CO_2 [*8]	298	*R*-3	-0.162	-0.162	0.368	0.038	B-PCD
	COKNIB	MOF	—	—	?	10	*R*-3	—	—	—	—	
	COKMOG	MOF	CO_2	N	CO_2 [*9]	10	*R*-3	-0.057	-0.057	0.056	-0.056	B-PCD
	COKNOH	MOF	—	—	?	10	*R*-3	—	—	—	—	
	COKMEW	MOF	CO_2	N	CO_2 [*10]	10	*R*-3	-0.242	-0.242	-0.003	-0.492	B-PCD
	COKPID	MOF	CO_2	N	CO_2 [*11]	10	*R*-3	-0.372	-0.372	0.142	-0.606	B-PCD
	COKQIE	MOF	CO_2	N	CO_2 [*12]	298	*R*-3	-0.061	-0.061	0.398	0.267	B-PCD
	COKNUN	MOF	—	—	?	10	*R*-3	—	—	—	—	
	COKMUM	MOF	CO_2	N	CO_2 [*13]	10	*R*-3	-0.027	-0.027	-0.057	-0.105	B-PCD
	COKPEZ	MOF	CO_2	N	CO_2 [*14]	10	*R*-3	-1.748	-1.748	-2.526	-5.899	B-PCD
101	EMAGOR	MOF	—	—	?	273	$I4_122$	—	—	—	—	
	EMAGUX	MOF	CO_2	N	1	195	$I4_122$	0.047	0.047	-0.126	-0.018	C-PCD
	EMAHAE	MOF	SO_2	N	1	293	$I4_122$	-0.114	-0.114	-0.278	-0.497	C-PCD
	EMAJAG	MOF	C_2H_2	N	1	293	$I4_122$	-0.074	-0.074	0.008	-0.138	C-PCD

							$I4_122$					
	EMAHEI	MOF	—	—	?	293	$I4_122$	—	—	—	—	
	EMAHIM	MOF	CO_2	N	1	195	$I4_122$	0.033	0.033	-0.033	0.024	C-PCD
	EMAHOS	MOF	SO_2	N	1	293	$I4_122$	0.073	0.073	0.033	0.105	A-PCD
	EMAHUY	MOF	C_2H_2	N	1	293	$I4_122$	-0.007	-0.007	-0.083	-0.099	A-PCD
102	HAFGUT	MOF	—	—	0	7	$R3_2$	—	—	—	—	
	HAFDUQ	MOF	CO_2	N	CO_2 [*15]	7	$R3_2$	-0.149	-0.149	0.086	-0.204	B-PCD
	HAFFAY	MOF	CO_2	N	CO_2 [*16]	7	$R3_2$	-0.473	-0.473	0.162	-0.774	B-PCD
103	OKUSOE	MOF	—	—	0	111	*F*23	—	—	—	—	
	OKUTAR	MOF	CH_4	Y	?	111	*Pa*-3	n/a	n/a	n/a	-52.754	B-PCD
	OKUTEV	MOF	CH_4	Y	?	111	*Pa*-3	n/a	n/a	n/a	-0.617	B-PCD
	OKUSUK	MOF	CH_4	N	?	111	*F*23	-0.121	-0.121	-0.121	-0.361	B-PCD
104	FALTUK	MOF	—	—	?	7	*P*4/*mnc*	—	—	—	—	
	FAMQAO	MOF	CO_2	N	CO_2 [*17]	7	*P*4/*mnc*	-0.133	-0.133	0.184	-0.084	B-PCD
105	CIRKAT	PCP	—	—	0	383	*C*2/*c*	—	—	—	—	
	CIRDEQ	PCP	CO_2	N	1	298	*C*2/*c*	1.633	-3.523	-0.206	-2.139	C-SCD
	CIRGET	PCP	CO_2	N	1	273	*C*2/*c*	1.984	-4.431	0.027	-2.497	D-SCD
	CIRGAP	PCP	CO_2	N	1	195	*C*2/*c*	-6.803	9.668	1.211	3.571	D-SCD
	CIRFES	PCP	C_2H_6	N	1	298	*C*2/*c*	0.913	-2.251	0.221	-1.133	C-SCD
	CIRFAO	PCP	C_2H_6	N	1	273	*C*2/*c*	-13.619	16.913	3.054	4.089	D-SCD
	CIRDUG	PCP	C_2H_6	N	1	185	*C*2/*c*	-9.284	13.238	2.152	4.947	D-SCD
	CIRDOA	PCP	C_2H_4	N	1	298	*C*2/*c*	0.552	-1.715	-0.033	-1.202	C-SCD
	CIRFUI	PCP	C_2H_4	N	1	273	*C*2/*c*	-12.933	15.957	2.591	3.579	D-SCD
	CIRDIU	PCP	C_2H_4	N	1	170	*C*2/*c*	-9.814	13.784	2.011	4.687	D-SCD
	CIRDAM	PCP	C_2H_2	N	1	318	*C*2/*c*	-0.381	-0.268	-0.658	-1.298	C-SCD

	AFUFEQ	PCP	C_2H_2	N	1	273	*C*2/*c*	-8.160	11.769	1.091	3.795	D-SCD
	CIRCUF	PCP	C_2H_2	N	1	195	*C*2/*c*	-7.922	11.578	1.136	3.919	D-SCD
106	DAXNEY	MOF	—	—	?	398	*P*4/*mmm*	—	—	—	—	
	DAXNUO	MOF	CO_2	N	?	298	*P*4/*mmm*	-0.601	-0.601	-0.154	-1.351	D-SCD
107	INOSUC	PCP	—	—	?	173	*C*2/*c*	—	—	—	—	
	INOTAJ	PCP	CO_2	N	6	195	*C*2/*c*	1.515	-0.024	0.452	1.952	D-SCD
108	IVEMIH	MOF	—	—	?	100	$P2_1/n$	—	—	—	—	
	IVEMED	MOF	CO_2	Y	1	150	$P2_1/c$	n/a	n/a	n/a	9.470	C-SCD
	IVELUS	MOF	C_3H_8	Y	1	150	$P2_1/c$	n/a	n/a	n/a	9.108	C-SCD
109	HAGTIV	MOF	—	—	?	100	*R*-3	—	—	—	—	
	KOZSID	MOF	CO_2	N	?	170	*R*-3	0.227	0.227	0.681	1.145	D-SCD
110	KUZQOO	MOF	—	—	0	290	$P4_2/mnm$	—	—	—	—	
	KUZQUU	MOF	CO_2	N	1	270	$P4_2/mnm$	-0.226	-0.226	0.213	-0.240	D-SCD
	KUZRAB	MOF	SO_2	N	1	270	$P4_2/mnm$	-0.028	-0.028	0.071	0.014	D-SCD
	KUZREF	MOF	SO_2	N	1	270	$P4_2/mnm$	-0.155	-0.155	0.040	-0.251	D-SCD
111	MAKPOH	MOF	—	—	?	103	*P*-1	—	—	—	—	
	MAKPUN	MOF	CO_2	Y	0.03	195	*C*2/*c*	n/a	n/a	n/a	15.055	D-SCD
	MAKQAU	MOF	CO_2	Y	0.2 bar [*18]	195 [*18]	*P*-1	n/a	n/a	n/a	23.218	D-SCD

112	BEKRUH	MOF	—	—	?	393	*P4/ncc*	—	—	—	—	
	BEKSAO	MOF	CO_2	N	1	100	*P4/ncc*	0.206	0.206	0.458	0.871	D-SCD
113	ECIWUJ02	MOF	—	—	0	298	*P*-1	—	—	—	—	
	ECIWUJ01	MOF	CO_2	Y	10	298	*P*-1	n/a	n/a	n/a	14.683	A-SCD
	ETIPII	MOF	CO_2	Y	10	253	$P2_1/c$	n/a	n/a	n/a	20.167	C-SCD
	ECIWUJ04	MOF	CO_2	Y	20	298	$P2_1/c$	n/a	n/a	n/a	18.789	A-SCD
114	DEFGIH	MOF	—	—	?	112	*P*−1	—	—	—	—	
	DEFGUT	MOF	CO_2	N	1	195	*P*−1	-0.365	0.780	6.989	7.549	C-PCD
	DEFHEE	MOF	NO	N*	1	125	*P*−1	n/a	n/a	n/a	17.676	C-PCD
	DEFHII	MOF	O_2	N*	1	95	*P*−1	n/a	n/a	n/a	13.121	C-PCD
	DEFHAA	MOF	N_2	N*	1	80	*P*−1	n/a	n/a	n/a	13.789	C-PCD
115	DITJEY	MOF	—	—	?	RT	$I4_1/amd$	—	—	—	—	
	DITJOI	MOF	SO_2	N	1	RT	$I4_1/amd$	-0.135	-0.135	2.869	2.588	C-PCD
	DITHAS	MOF	CO_2	N	1	100	$I4_1/amd$	-0.084	-0.084	2.783	2.600	C-PCD
	DITHEW	MOF	CO_2	N	1	150	$I4_1/amd$	-0.233	-0.233	2.965	2.485	C-PCD
	DITHEW01	MOF	CO_2	N	1	198	$I4_1/amd$	-0.191	-0.191	3.137	2.744	C-PCD
	DITHOG	MOF	CO_2	N	1	230	$I4_1/amd$	-0.158	-0.158	2.620	2.290	C-PCD
	DITHUM	MOF	CO_2	N	1	270	$I4_1/amd$	-0.065	-0.065	1.501	1.368	C-PCD
	DITJAU	MOF	—	—	?	RT	$I4_1/amd$	—	—	—	—	C-PCD
	DITJIC	MOF	SO_2	N	1	RT	$I4_1/amd$	0.088	0.088	-0.431	-0.261	C-PCD
	DITROQ01	MOF	CO_2	N	1	7	$I4_1/amd$	0.000	0.000	-0.183	-0.183	C-PCD
	DITRIK	MOF	CO_2	N	1	117	$I4_1/amd$	-0.102	-0.102	-0.541	-0.751	C-PCD
	DITROQ	MOF	CO_2	N	1	150	$I4_1/amd$	-0.079	-0.079	-0.413	-0.567	C-PCD
	DITRUW	MOF	CO_2	N	1	198	$I4_1/amd$	-0.047	-0.047	-0.229	-0.318	C-PCD
	DITSAD	MOF	CO_2	N	1	230	$I4_1/amd$	-0.037	-0.037	-0.128	-0.207	C-PCD
	DITGUL	MOF	CO_2	N	1	273	$I4_1/amd$	-0.023	-0.023	0.000	-0.047	C-PCD

116	FAQLER	MOF	—	—	?	150	$I4_122$	—	—	—	—	
	FAQLUH	MOF	CO_2	N	1 CO_2 per V	7	$I4_122$	0.324	0.324	-0.215	0.425	D-PCD
	FAPXEC	MOF	CO_2	N	2 CO_2 per V	7	$I4_122$	0.291	0.291	0.298	0.278	D-PCD
	FAQLIV	MOF	—	—	?	150	$I4_122$	—	—	—	—	
	FAPXIG	MOF	CO_2	Y	1 CO_2 per V	7	$P4_122$	0.120	0.120	0.209	0.447	D-PCD
	FAPXOM	MOF	CO_2	Y	2 CO_2 per V	7	$P4_122$	0.100	0.100	0.193	0.394	D-PCD
117	FATMAR	CP	—	—	?	120	$P6_3/m$	—	—	—	—	
	FATMAR01	CP	—	—	0	240	$P6_3/m$	1.119	1.119	1.502	0.000	
	FATKIX	CP	CO_2	N	6	298	$P6_3/m$	1.948	1.948	2.256	2.404	C-PCD
	FATKOD	CP	C_2H_4	N	6	298	$P6_3/m$	1.967	1.967	2.339	2.515	C-PCD
	FATKUJ	CP	CH_4	N	6	298	$P6_3/m$	1.919	1.919	2.334	2.420	C-PCD
118	KOCFAM	MOF	—	—	?	296	*I4/mmm*	—	—	—	—	
	KOCDEO	MOF	CO_2	N	?	300	*I4/mmm*	-0.106	-0.106	2.080	1.863	D-PCD
119	LIGSAZ	MOF	—	—	0	298	*C2/c*	—	—	—	—	
	LIGSED	MOF	CO_2	N	1.2	313	*C2/c*	-0.113	6.694	4.646	1.932	C-PCD
120	DESTUU01	MOF	—	—	0	300	*Im*-3	—	—	—	—	
	DESVAC01	MOF	CO_2	N	0.5	320	*Im*-3	-0.009	-0.009	-0.009	-0.012	C-PCD
	DESVAC	MOF	CO_2	N	1	320	*Im*-3	-0.026	-0.026	-0.026	-0.055	C-PCD
	DESVAC02	MOF	CO_2	N	1.5	320	*Im*-3	-0.026	-0.026	-0.026	-0.067	C-PCD
121	LIWBIE	PCP	—	—	0	100	*P*-1	—	—	—	—	
	LIWBEA	PCP	CO_2	N	0.2	195	*P*-1	11.504	4.007	-6.367	9.077	D-PCD

	LIWBOK	PCP	NO	N	0.2	121	*P*-1	3.416	12.871	-6.968	9.172	C-PCD

Supplementary Table 5. Reported examples of static gas inclusion.

DOI	CCDC#	Host	Guest	Phase change	Pressure (bar)	Temp. (K)	Space group	e_1 / %	e_2 / %	e_3 / %	ΔVol / %	Category
122	RAVCAU01	OH	—	—	0	100	*R*-3	—	—	—	—	
	RAVCAU02	OH	—	N	0	293	*R*-3	0.612	0.612	1.435	2.681	
	QIRKUZ	OH	CO_2	N	0.2	100	*R*-3	-0.272	-0.272	-0.051	-0.599	A-SCD
	QIRLAG	OH	CO_2	N	2	100	*R*-3	2.844	2.844	0.137	-1.048	A-SCD
123	QIGBEN01	POM	—	—	?	295	$P2_1/n$	—	—	—	—	
	EJIMOZ	POM	C_2H_3Br	Y	Liquid	295	$P4/n$	0.994	1.531	0.074	2.607	E-SCD
	EJIMUF	POM	C_2H_3Br	Y	Liquid	295	$P4_2/n$	-0.150	0.381	-0.222	0.000	E-SCD
124	WUVKON	POM	—	—	?	298	$P6_3/m$	—	—	—	—	
	HUVBEF	POM	CH_2FCF_3	Y	Condensed	298	*P*-3	2.064	2.064	-2.185	1.897	E-SCD
	WUVKED	POM	$CBrF_3$	N	Condensed	173	$P6_3/m$	1.003	1.003	-2.147	-0.176	F-SCD
	WUVKUT	POM	CCl_3F	N	Condensed	295	$P6_3/m$	2.042	2.042	-2.049	1.992	F-SCD
	WUVLAA	POM	$CBrCl_2F$	N	Condensed	295	$P6_3/m$	2.086	2.086	-2.101	2.026	F-SCD
	WUVLEE	POM	C_2F_6	N	Condensed	295	$P6_3/m$	2.115	2.115	-2.315	1.861	F-SCD
	WUVLII	POM	CF_4	N	Condensed	295	$P6_3/m$	1.891	1.891	-1.838	1.911	F-SCD
	WUVLII01	POM	CF_4	N	Condensed	173	$P6_3/m$	1.944	1.944	-2.024	1.823	F-SCD
	WUVLOO	POM	CH_4	N	Condensed	295	$P6_3/m$	1.778	1.778	-1.846	1.677	F-SCD
125	ILUTIU	POM	—	—	?	296	*C*2/*c*	—	—	—	—	
	ILUGAZ	POM	CH_4	N	36	100	*C*2/*c*	-1.137	0.314	-0.981	-1.795	C-SCD
	ILUHII	POM	Kr	N	9.8	100	*C*2/*c*	-1.074	-0.267	-0.860	-2.186	C-SCD
	ILUHUU	POM	N_2	N	80	100	*C*2/*c*	-1.132	-0.180	-781.000	-2.081	C-SCD

ILUJIK	POM	N_2	N	80	100	*C*2/*c*	-0.870	0.068	-0.618	-1.416	C-SCD
ILUJUW	POM	N_2	N*	80	100	*C*2/*c*	-1.017	-0.100	-0.785	-1.892	C-SCD
ILUKAD	POM	CO_2	N	?	100	*C*2/*c*	-1.249	-0.019	-1.022	-2.276	D-SCD
ILUKIL	POM	CO_2	N	?	100	*C*2/*c*	-1.281	1.059	0.070	-2.258	D-SCD
ILUKOR	POM	Kr	N	9.8	100	*C*2/*c*	-1.037	0.059	-1.053	-2.020	C-SCD
ILUKUX	POM	C_2H_6	N	9.8	100	*C*2/*c*	-0.985	0.222	-0.822	-1.578	C-SCD
ILUKUX01	POM	C_2H_6	N	9.8	100	*C*2/*c*	-0.894	0.287	-0.815	-1.417	C-SCD
ILULAE	POM	Ar	N	80	100	*C*2/*c*	-1.056	-0.110	-1.040	-2.192	C-SCD
ILULEI	POM	C_2H_4	N	9.8	100	*C*2/*c*	-1.123	-1.307	0.534	-1.888	C-SCD
ILULIM	POM	C_2H_4	N	9.8	100	*C*2/*c*	-1.166	-1.333	0.497	-1.992	C-SCD
ILUPOW	POM	C_2H_4	N	9.8	100	*C*2/*c*	-0.934	0.329	-0.982	-1.580	C-SCD
ILUQAJ	POM	Xe	N	9.8	100	*C*2/*c*	-1.176	0.713	-1.343	-1.798	C-SCD
ILUQEN	POM	CH_4	N	36	100	*C*2/*c*	-1.159	0.361	-0.990	-1.780	C-SCD
ILUQIR	POM	Xe	N	9.8	100	*C*2/*c*	-1.084	0.763	-1.259	-1.574	C-SCD
ILUQIR01	POM	Xe	N	9.8	100	*C*2/*c*	-1.067	0.801	-1.279	-1.540	C-SCD

Descriptions of categories

A-SCD	Single-crystal X-ray diffraction data. Article includes apohost structure and structure(s) of crystals pressurized with a particular gas. The gas pressure of the crystal environment during data collection is known and the data collection temperatures for all crystals are constant.
B-SCD	Single-crystal X-ray diffraction data. Article includes apohost structure and structure(s) of crystals pressurized with a particular gas. The gas pressure of the crystal environment during data collection is unknown (either due to temperature changes or gas loading methodologies) and the data collection temperatures are constant.
C-SCD	Single-crystal X-ray diffraction data. Article includes apohost structure and structure(s) of crystals pressurized with a particular gas. The gas pressure of the crystal environment during data collection is known but the data collection temperatures for the apohost and pressurized crystal(s) are different.
D-SCD	Single-crystal X-ray diffraction data. Article includes apohost structure and structure(s) of crystals pressurized with a particular gas. The gas pressure of the crystal environment during data collection is unknown (either due to temperature changes or gas loading methodologies) and the data collection temperatures for the apohost and pressurized crystal(s) are different.
E-SCD	Single-crystal X-ray diffraction data. Article includes apohost structure and structure(s) of crystals that had been immersed in the liquified phase of a substance considered to be a gas at RT. The data collection temperatures are constant.

F-SCD	Single-crystal X-ray diffraction data. Article includes apohost structure and structure(s) of crystals that had been immersed in the liquid phase of a substance considered to be a gas at RT. The data collection temperature for the apohost differs from those for the guest-loaded crystals.

A-PCD	Powder X-ray diffraction data. Article includes apohost structure and structure(s) of crystals pressurized with a particular gas. The gas pressure of the crystal environment during data collection is known and the data collection temperatures are constant.
B-PCD	Powder X-ray diffraction data. Article includes apohost structure and structure(s) of crystals pressurized with a particular gas. The gas pressure of the crystal environment during data collection is unknown (either due to temperature changes or gas loading methodologies) and the data collection temperatures are constant.
C-PCD	Powder X-ray diffraction data. Article includes apohost structure and structure(s) of crystals pressurized with a particular gas. The gas pressure of the crystal environment during data collection is known but the data collection temperatures for the apohost and pressurized crystal(s) are different.
D-PCD	Powder X-ray diffraction data. Article includes apohost structure and structure(s) of crystals pressurized with a particular gas. The gas pressure of the crystal environment during data collection is unknown (either due to temperature changes or gas loading methodologies) and the data collection temperatures for the apohost and pressurized crystal(s) are different.

Host abbreviations

MOF	Metal-organic framework
POM	Porous organic material
HOF	Hydrogen-bonded organic framework
PCP	Porous coordination polymer
XBF	Halogen-bonded framework
CP	Coordination polymer
CC	Discrete coordination complex
OH	Organic host

Additional footnotes

* Change in unit cell choice relative to the reference structure (the strain eigenvalues cannot be determined directly)

*1 Conflicting information: CSD states space group $P2_1/n$

*2 Structure could not be fully activated (some DMF remained in the sample post activation)
*3 CCDC REFCODE not found for this structure; parameters taken directly from article
*4 CSD does not reports nitrogen included in the structure
*5 Dosed with 0.5 CO_2 per Co
*6 Dosed with 1.5 CO_2 per Co
*7 Dosed with 0.5 CO_2 per Zn
*8 Dosed with 1.5 CO_2 per Zn
*9 Dosed with 0.5 CO_2 per Cu
*10 Dosed with 0.35 CO_2 per Fe
*11 Dosed with 1.3 CO_2 per Fe
*12 Dosed with 1.5 CO_2 per Fe
*13 Dosed with 0.5 CO_2 per Mn
*14 Dosed with 0.75 CO_2 per Mg
*15 Dosed with 1.8 CO_2 per Cu
*16 Dosed with 2.3 CO_2 per Cu
*17 Dosed with 1.75 CO_2 per Cu
*18 Ambiguous information: pressure was either 0.2 or 1 bar, and the temperature of data collection was either 195 or 190 K
*19 Dosed with 1 CO_2 per V
*20 Dosed with 2 CO_2 per V

Supplementary Text 9. Visualizing molecular-level structural changes with gas pressure

In situ single-crystal X-ray diffraction analysis of the series **T1**$_{Cx}$ yielded models that enabled visualization of the subtle structural changes experienced by the **T1** crystal as a result of variable-pressure exposure to CO_2 gas. The programs X-Seed and Mercury were used to generate images that were used to construct video files to aid our understanding of pressure-dependent molecular rearrangements.

Video 1 shows gradual enlargement of the 0D voids in the series **T1**$_{Cx}$ with increasing CO_2 pressure. Videos 2 and 3 are best viewed using a suitable video player set to automatically repeat playback. Video 2 consists of only two frames, which alternate between the crystal structures (viewed along [001]) recorded for a crystal of **T1** exposed to 0 and 32 bar CO_2 pressure. Video 3 (also viewed along [001]) shows progression through all of the variable-pressure crystal structures. Video 4 (viewed along [010]) shows enlargement of the 1D channels of **T1**$_{Cx}$ with increasing pressure; as the host molecules move apart along [001] to accommodate gas molecules within the growing 0D voids, they allow space for concave indentations to form along the channels (indicated with a yellow ellipse). Video 5 comprises two frames and shows enlargement of the 0D voids due to CO_2 pressure in **T1**$_{C32}$ relative to those of **T1**$_{0}$ (*cf* Supplementary Fig. 4). Video 6 shows a simplified model of the trianglimine molecules (see Supplementary Fig. 9) of **T1**$_{Cx}$ viewed perpendicular to [001]; the triangles represent **T1** molecules and five successive hexameric assemblies of **T1** are shown stacked along [001]. Red and grey triangles are used to distinguish between the two crystallographically-independent molecules.

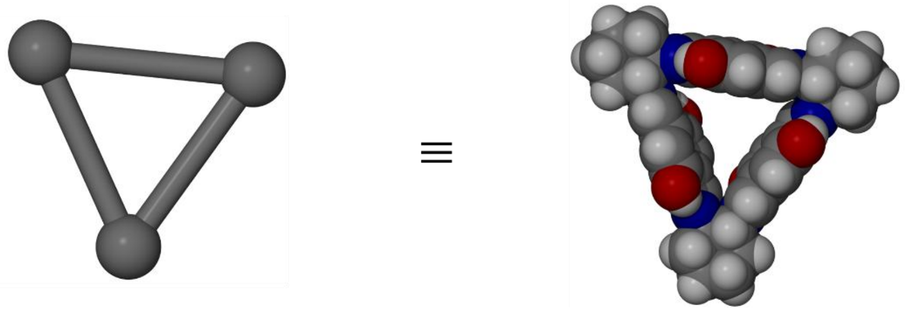

Supplementary Fig. 9. Simplified representation of a trianglimine molecule. X-Seed was used to generate a centroid for each cyclohexane moiety and then all the remaining atoms were deleted. The three centroids were then bonded to each other to create a triangle. The centroids were assigned radii of 2 Å to better visualize their roles in capping the intrinsic voids of neighboring **T1** molecules. This simplified model was used to generate Video 6.

Supplementary Text 10. Comment on electron counts based on difference electron density maps

The SQUEEZE[126] routine, as implemented by the Platon[127] software package, is primarily designed to analyze the residual electron density of a crystallographic model derived from single-crystal X-ray crystallographic data. SQUEEZE can locate and quantify probe-accessible regions in a crystal structure

and enumerate the apparent number of unmodelled electrons in these spaces. However, high-quality data are required for these electron counts to be credible. We acknowledge that the diffraction data obtained during the present study are not of sufficient quality due to several factors, which include (1) the presence of large spaces in which it is not possible to model guest atoms, (2) mechanical stress experienced by the crystal due to pressure-induced structural changes, (3) the necessity of recording room-temperature data, and (4) the use of a glass capillary to house the crystal.

The main evidence that we used to infer that CO_2 molecules are sorbed into the 0D voids of $\mathbf{T1}_{\mathbf{C}x}$ is that the cavities (of the time-averaged crystal structures) become sufficiently enlarged with increasing gas pressure. This enlargement of the cavities was not observed during our previous study[50], where we showed that water molecules are only accommodated in the 1D channels and do not access the 0D voids. However, using SQUEEZE we obtained increasing electron counts in the 0D cavities of $\mathbf{T1}_{\mathbf{C}x}$ with increasing pressure (i.e. 1 electron per cavity in $\mathbf{T1}_{\mathbf{0}}$ and 20 or 21 electrons per cavity in $\mathbf{T1}_{\mathbf{C32}}$; a molecule of CO_2 contains 22 electrons). Although we do not regard these calculations as conclusive evidence (for the reasons stated above), they serve to support our claim that gas molecules increasingly occupy the expanding 0D voids as the gas pressure increases.

Supplementary Text 11. Fitting the pressure-dependent change in *c* using sorption models

Three of the most commonly used empirical models for sorption isotherms include the Langmuir[128], Sips[61] and Toth[129] equations. They differ slightly in their mathematical forms and the types of sorption behavior they are best suited to describe. We can express the isotherm equations as functions of Δc *vs* P as follows:

**Model**	**Equation**
Langmuir	$\Delta c = \dfrac{\Delta c_{max} K P}{1 + KP}$
Sips	$\Delta c = \dfrac{\Delta c_{max} (KP)^{\frac{1}{m}}}{1 + (KP)^{\frac{1}{m}}}$
Toth	$\Delta c = \dfrac{\Delta c_{max}\, P}{(K + P^m)^{1/m}}$

where Δc_{max} represents the maximum percentage change in c at full loading of the **T1** cavities, and K and m are constants. The Sips and Toth equations are modifications of the Langmuir equation. The Sips equation is versatile and can describe both Langmuir and Freundlich behavior, depending on the value of the heterogeneity factor m. When $m = 1$, the Sips equation reduces to the Langmuir equation, and when $m \neq 1$ the equation accounts for deviations from ideal Langmuir behavior. The Toth equation is particularly useful for describing adsorption on heterogeneous surfaces, and systems where the

adsorption energy decreases as the surface coverage increases. It can model both Type I and Type II sorption isotherms, making it suitable for a wide range of sorption processes. However, the Sips equation is more generally suitable for describing a wide range of sorption behaviors, and capturing complex sorption phenomena. The choice between the equations depends on the specific characteristics of the system being studied and the type of sorption behavior observed.

We used the pressure-dependent percentage change in the length of the *c* axis as a proxy for **T1** cavity gas loading (i.e. up to 1 molecule of gas per cavity at full loading). Data were analyzed using Microsoft Excel and the parameters for the various isotherm models were determined using the Solver add-in. For each model, the procedure involved estimating initial values of Δc_{max}, K and $1/m$, and employing these using the relevant isotherm equation to calculate the values in the Fit column as a function of pressure. The differences between the fitted and experimental Δc values are computed in Column R, and squared in Column R^2. The use of the Solver add-in involved minimizing the sum of the residuals R^2 by optimizing Δc_{max}, K and $1/m$. Plots of the experimental and fitted data for $\mathbf{T1}_{\mathbf{C}x}$ are shown in Supplementary Fig. 10. The Sips equation provided the best fit to the $\mathbf{T1}_{\mathbf{C}x}$ data, and also yielded a reasonable estimate of Δc_{max} = 10.051 Å.

Fitting Δc data for $\mathbf{T1}_{\mathbf{C}x}$

Langmuir fit

P / bar	Δc / %	Fit_L	R	R^2
0	0.000	0.000	0.0000	0.0000
1	0.259	0.625	0.3664	0.1343
2	1.015	1.202	0.1869	0.0349
4	1.538	2.230	0.6917	0.4784
8	4.232	3.895	-0.3365	0.1133
16	6.663	6.217	-0.4461	0.1990
32	8.622	8.858	0.2358	0.0556

Residual and optimized Langmuir parameters

ΣR^2	Δc_{max}	K
1.0155	15.396	0.042

Sips fit

P / bar	Δc / %	Fit_S	R	R^2
0	0.000	0.000	0.0000	0.0000
1	0.259	0.253	-0.0058	0.0000
2	1.015	0.717	-0.2982	0.0889
4	1.538	1.867	0.3295	0.1086
8	4.232	4.062	-0.1700	0.0289
16	6.663	6.718	0.0544	0.0030
32	8.622	8.613	-0.0087	0.0001

Residual and optimized Sips parameters

ΣR^2	Δc_{max}	K	$1/m$
0.2295	10.051	0.098	1.571

Toth fit

P / bar	Δc / %	Fit_T	R	R^2
0	0.000	0.000	0.0000	0.0000
1	0.259	0.508	0.2489	0.0620
2	1.015	1.015	0.0000	0.0000
4	1.538	2.020	0.4822	0.2325
8	4.232	3.919	-0.3127	0.0978
16	6.663	6.704	0.0408	0.0017
32	8.622	8.649	0.0271	0.0007

Residual and optimized Toth parameters

ΣR^2	Δc_{max}	K	m
0.3946	9.290	2853.583	2.737

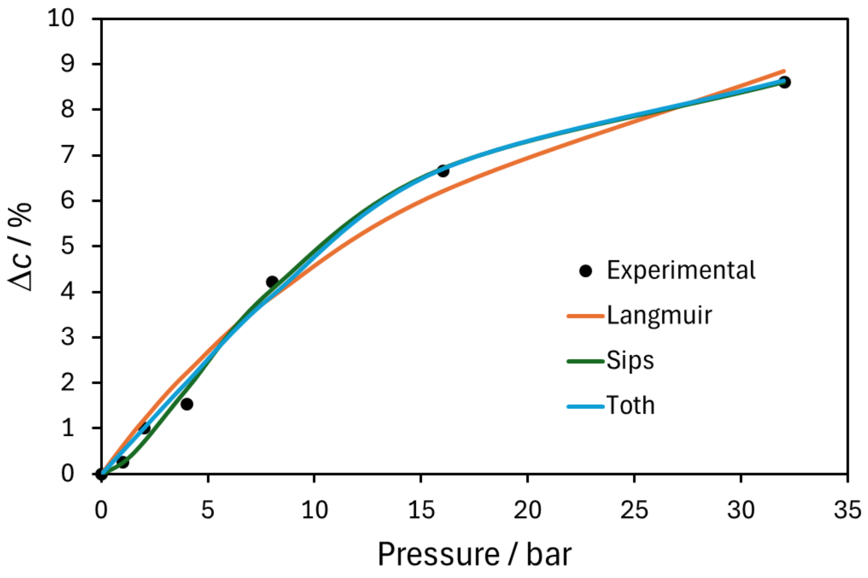

Supplementary Fig. 10. Plot of experimental and fitted data for $\mathbf{T1}_{Cx}$.

Supplementary Text 12. Variable-pressure *in situ* photomicroscopy

In situ microscopy experiments were carried out to record macroscopically visible changes in the dimensions of single crystals exposed to gas pressure at variable-pressure ramp rates. The apparatus required for these experiments (Supplementary Fig. 11) was developed in-house and consisted of a gas supply connected to a software-controlled electronic gas valve, which was in turn connected to a stainless-steel pressure chamber equipped with 6 mm thick quartz windows. The pressure chamber is attached to the sample stage of a microscope fitted with a USB camera. Experiments were controlled and monitored using bespoke software (Pressure_Valve, developed by L. J. Barbour). For the current study, photomicrographs were recorded at 30 second intervals while exposing crystals to gas at a ramp rate of 0.2 bar min^{-1}. In a typical experiment the pressure was increased from 0 bar to a selected maximum pressure, followed by a decrease in pressure back to 0 bar. In some cases, the pressure profile was cycled three times. Side-by-side combination of the time-lapse images of a crystal, together with projections of the corresponding crystal structures, facilitate a partial rationalization of how gas-induced changes at the molecular level affect the macro-scale dimensions of the crystal (e.g., Video 7).

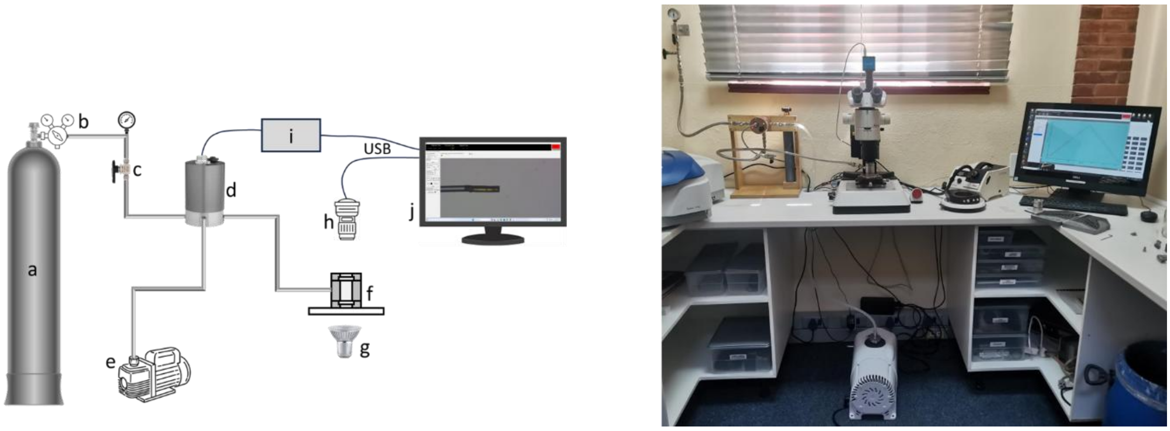

Supplementary Fig. 11. Apparatus used to record variable-pressure photomicrographs. The diagram on the left shows the test gas supply (a), pressure regulator (b), isolation ball valve (c), electronic pressure valve (d), scroll pump (e), sample pressure cell (f), transilluminator bulb (g), USB camera (h), USB interface with analogue-to-digital conversion and digital input/output capability (i) and a computer for control and monitoring experiments. A photograph of the apparatus is shown on the right.

<u>Supplementary Text 12.1. Sample chamber</u>

A cross section of the custom-constructed high-pressure sample chamber is shown in Supplementary Fig. 12. The stainless-steel cylinder is capped at each end by means of a lid fitted with a fused quartz window. Each lid is fastened to the cylinder by means of three steel Allen-cap screws and O-rings are used to provide a gas-tight seal between the pressure chamber and the windows. The chamber is secured to a rotation-translation stage and the configuration allows viewing the contents of the pressure chamber using illumination from below. Gas pressure within the sample chamber can be controlled *via* a high-pressure steel-braided hose.

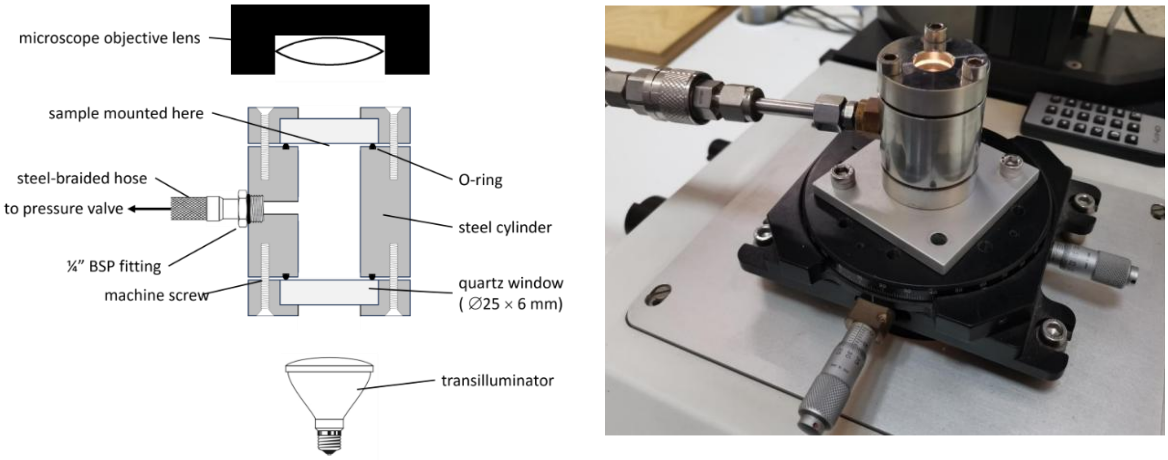

Supplementary Fig. 12. Cross-sectional diagram (left) and a photograph (right) of the sample pressure chamber.

Supplementary Text 12.2. Software control

The two primary capabilities of the software (Supplementary Figs 13 and 14) are to control pressure and to capture time-lapse photomicrographs. The status bar at the top of the window shows elapsed time, current pressure in bar, and the current pressure setpoint of the experiment. The pressure control and time-lapse photography functionalities are each accessed *via* a tabbed page. The time-lapse photography page (Supplementary Fig. 13) allows selection of a video capture source, setting the image aspect ratio, and selection of scale-bar settings. For each magnification detent setting the scale bar and aspect ratio can be calibrated against a 0.6 mm circle etched onto a calibration slide. Settings are also available to embed the elapsed time and current pressure in the photomicrographs. The "live" image streamed from the video source is also displayed on this tabbed page.

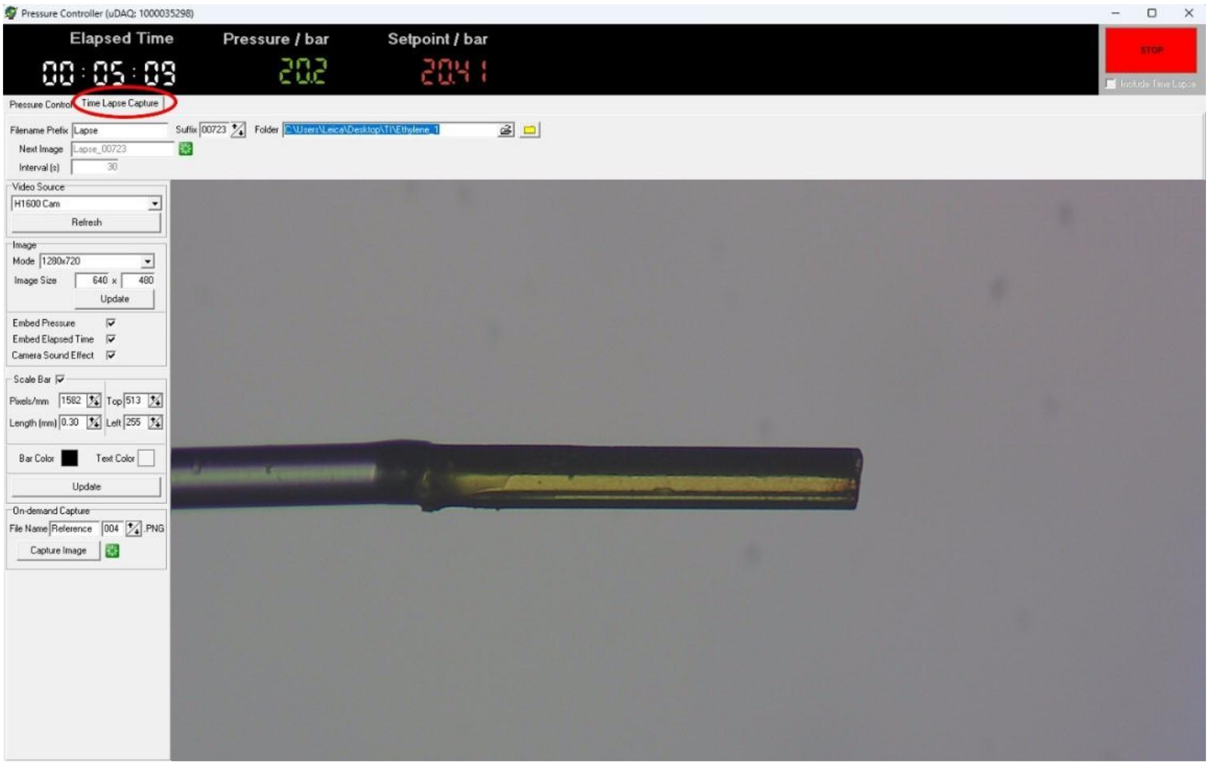

Supplementary Fig. 13. Screen capture of the "Time-Lapse Capture" tabbed page of the Pressure_Valve software.

The "Pressure Control" tab of the software (Supplementary Fig. 14) allows control of the pressure-time profile of the experiment. The output pressure of the electronic pressure valve is controlled by a sequential list of user-coded commands. Three separate commands are available:

`RELAY ON/OFF` controls power to the pressure valve. Once the experiment is completed it is useful to power the valve down to avoid excessive buildup of heat if the apparatus will be unattended for an extensive period of time (e.g. an overnight experiment). This command is typically inserted at the beginning and end of each experiment.

GOTOP *P R H* instructs the pressure valve to reach a final pressure of *P* bar at a rate of *R* bar min^{-1}, and to then pause (hold) at that pressure for *H* minutes before proceeding to the next step. If the pressure cannot be reached in the combined time allowed for the ramp and hold times, then the program proceeds to the next step.

PAUSE *H* instructs the pressure valve to pause (hold) at the current pressure setpoint for *H* minutes.

When the "START" button is pressed, the program first plots the pressure-time profile of the intended experiment. Blue segments indicate pressure-control processes and white segments indicate pauses. During the experiment the current pressure is plotted in red. Ideally, the red plot should follow the simulated profile.

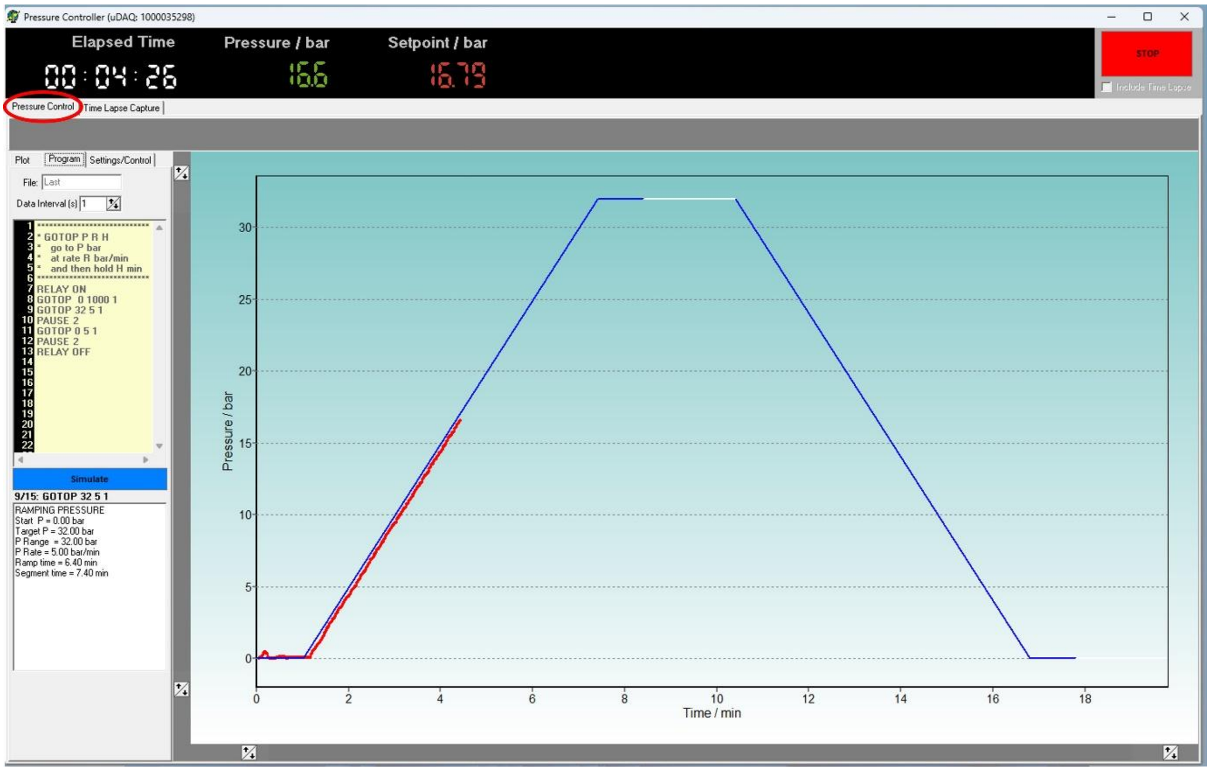

Supplementary Fig. 14. Screen capture of the "Pressure Control" tabbed page of the Pressure_Valve software.

Supplementary Text 12.3. Design considerations

The electronic pressure valve controls the output pressure at a given setpoint using two solenoid valves, one at the gas inlet port and the other at the exhaust port. The pressure valve maintains the setpoint by opening and closing these valves in response to comparing the output pressure to the setpoint – if the output pressure is too high the device opens the exhaust valve, and if the pressure is too low it opens the inlet valve. A vacuum scroll pump is attached to the exhaust port to allow the sample cell to reach sub-ambient pressures. If the volume of the sample chamber is small, then pulsing the solenoid valves can easily cause over- and undershoot of the pressure, resulting in unstable pressure control. Our design

compensated for this effect by employing inline flow-control valves at the inlet and exhaust ports of the pressure valve. A repurposed lecture bottle was also attached to the exhaust as a reservoir to buffer sudden changes in pressure. A photograph of the pressure-valve apparatus is shown in Supplementary Fig. 15.

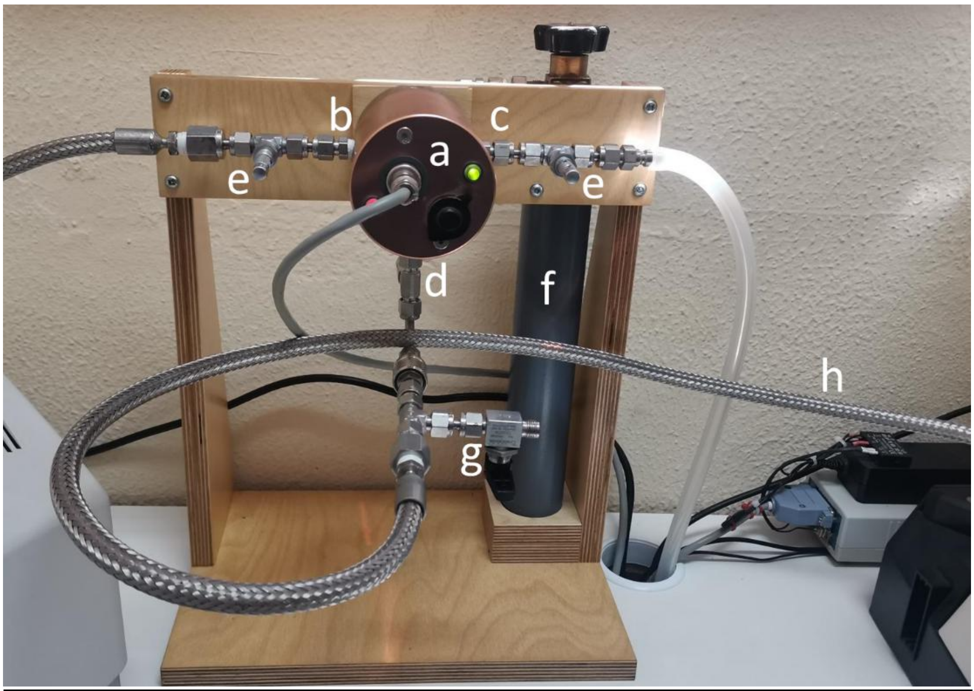

Supplementary Fig. 15. Photograph of the pressure-valve assembly. The apparatus consists of a ProportionAir pressure valve (a) with gas inlet (b), exhaust (c) and outlet (d) ports. Flow control valves (e) and a buffer reservoir (f) facilitate smoothing of the pressure control. The outlet pressure hose is fitted with a ball valve (g) to vent the pressure in the sample chamber, when required. The braided hose (h) connected to the outlet is used to deliver the gas to the sample chamber.

Supplementary Text 12.4. Experimental procedure

A suitable single crystal was selected for its elongated, broad and unfractured appearance. The crystal was attached to the tip of a thin glass fiber using epoxy resin, and the glass fiber was attached to one of the fused quartz windows of the sample cell. To avoid difficulties in maintaining focus on the entire crystal due to a shallow depth of view at high magnification, it was necessary to carefully align the crystal as parallel as possible to the window. The quartz window with the attached crystal was placed upside down at the upper end of the sample chamber so that the crystal would remain in focus throughout the experiment. This ensured that the focal path of the camera did not include the volume of the sample chamber where the density of the gas would be changing throughout the experiment. When the crystal had been attached to the window at the lower end of the chamber, changes in the density of the gas in the sample compartment altered the focus of the image. Attaching the crystal to the upper window minimized this effect.

Supplementary Text 12.5. Sources of commercially available components

Although parts of the pressure cell apparatus were constructed in-house, other components were obtained from commercial sources, as follows:

- Modular gas reticulation components: (Swagelok, USA).
- Digital USB camera: 16 MP 1080p Digital Industry Microscope Camera (Eakins, China).
- Microscope: Leica M165 high-performance stereo microscope with a magnification range of 7.3 – 120 and a KL 1500_{LCD} light source (Leica Microsystems).
- Electronic pressure valve: Model GP10A1000EEB2AI (ProportionAir, USA).
- Fused quartz windows: Model FQW-256 (UQG Optics, Cambridge, UK).
- Rotation stage: Model XYR1 Translation Stages with Rotating Platform (Thorlabs, USA).
- Calibration slide: standard calibration slide for USB microscope cameras, (Motic Scientific).
- Data acquisition module: Model USB-26D USB DAQ Unit 16/32 Channel 1MHz 16-bit A/D, 24 Digital I/O (Eagle Technology, South Africa).

Supplementary Text 13. Expansion and contraction of crystals due to changes in gas pressure

Six crystals (Supplementary Fig. 16) were subjected to variable-pressure photomicroscopy experiments. The laboratory temperature was controlled in the range 26 to 27 °C and Crystals 1 to 5 were exposed to CO_2 gas according to the details given below. Photomicrographs were recorded every 30 s and used to construct Videos 8 to 12 for Crystals 1 to 5, respectively, using the VideoPad Video Editor software package (https://www.nchsoftware.com/videopad/index.html). The images were also used to measure the extension of the crystal needle axis as a function of gas pressure. Horizontal measurements of crystal expansion and contraction were made in pixels using the program JRuler (https://jruler.software.informer.com/3.1/). In all cases the long (horizontal) axis of the crystal corresponds to the crystallographic *c* axis. In each case the percentage expansion of the crystal was plotted against pressure, and these data were modelled using the Sips equation (details given below).

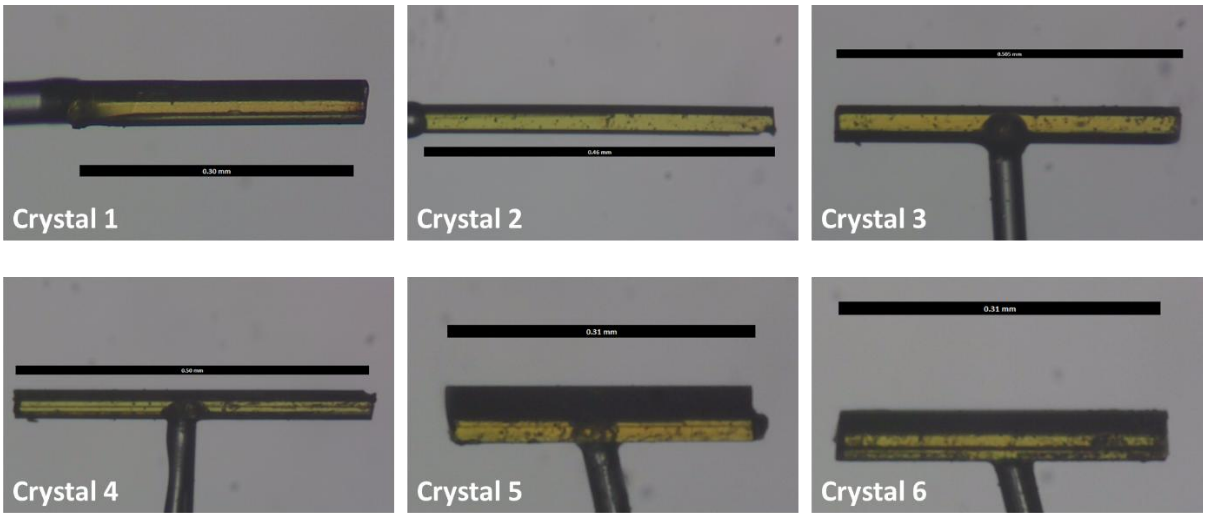

Supplementary Fig. 16. Photomicrographs of six different crystals of **T1** mounted in the variable-pressure cell shown in Supplementary Fig. 12. Scale bar lengths: 0.30, 0.46, 0.505, 0.50, 0.31 and 0.31 mm for Crystals 1 to 6, respectively. The crystals are triangular in cross-section, and they were illuminated from below. Moreover, it was difficult to control the orientations of the faces of the crystals during glue-up to minimize the appearance of darkened regions.

Supplementary Text 13.1. Crystal 1

Program: 0 $\rightarrow$ 32 $\rightarrow$ 0 bar at 0.2 bar min^{-1}.

Video file: Video 8

Comments: One end of the crystal was attached to a glass fiber by means of epoxy resin. Measurements of crystal extension with pressure for all four cycles are shown in Supplementary Fig. 17.

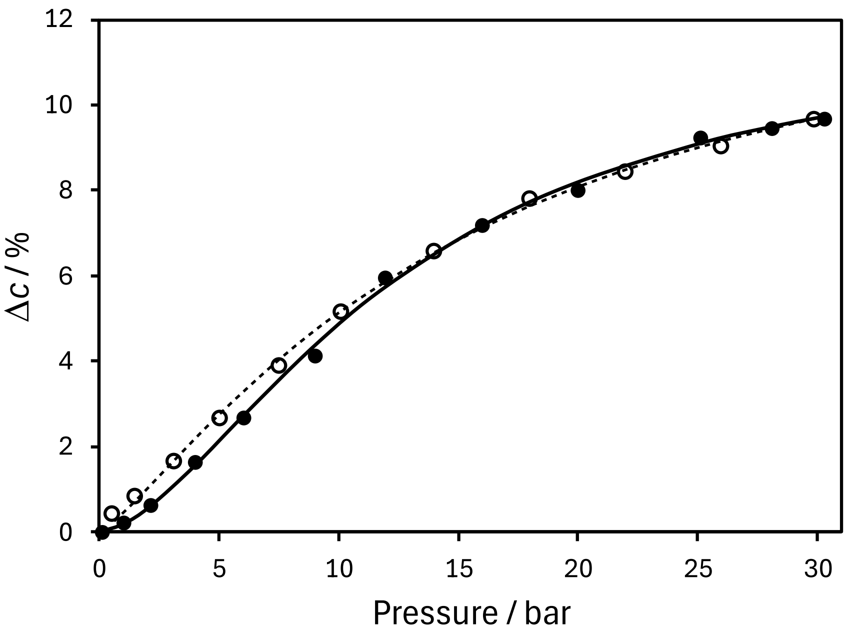

Supplementary Fig. 17. Plot of percentage expansion of the needle axis *c* of Crystal 1 with CO_2 pressure. Solid and open circles represent data recorded during increasing and decreasing pressure, respectively. The best-fit Sips models are shown as solid (increasing pressure) and dashed (decreasing pressure) lines.

Supplementary Text 13.2. Crystal 2

Program: Four cycles of 0 → 30 → 0 bar at 0.2 bar min^{-1}.

Video file: Video 9 (first cycle only)

Comments: One end of the crystal was attached to a glass fiber by means of epoxy resin. The crystal bent slightly during adsorption and desorption. Measurements of crystal extension for all four cycles are shown in Supplementary Fig. 18. The sample chamber had been opened between the first and second cycles, which admitted some atmospheric water. During the second cycle the crystal initially changed color from yellow to red (indicating water uptake) and experienced severe bending, starting at 2 bar. However, it recovered its straight shape and yellow color by 10 bar. The presence of water likely accounts for the inflection in the expansion of the crystal in the range 2 to 10 bar. We observe that there is almost no hysteresis by the fourth cycle, possibly indicating that conditioning by repeated cycling results in a more consistent response of the crystal shape to pressure during both adsorption and desorption. The best Sips models for all four cycles during adsorption are shown in Supplementary Fig. 19.

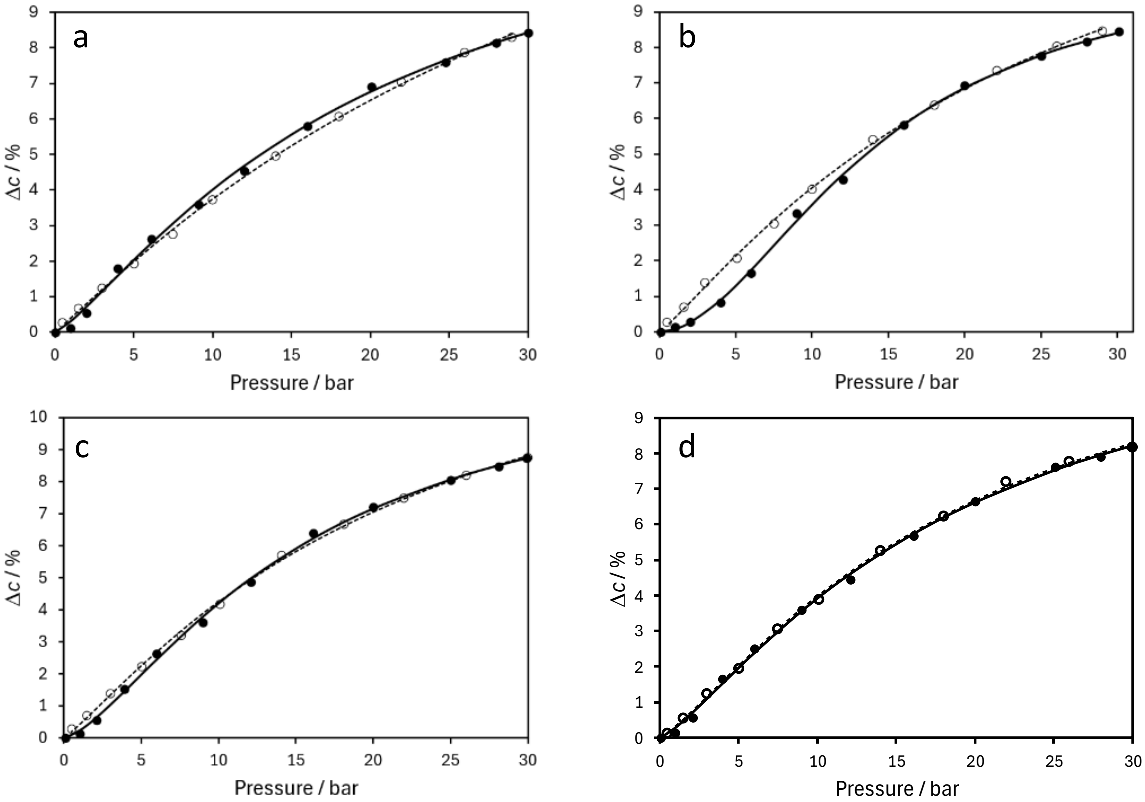

Supplementary Fig. 18. (a) – (d) Plots of percentage expansion of the needle axis *c* of Crystal 2 with CO_2 pressure during cycles 1-4, respectively. Solid and open circles represent data recorded during increasing and decreasing pressure, respectively. The best-fit Sips models are shown as solid (increasing pressure) and dashed (decreasing pressure) lines.

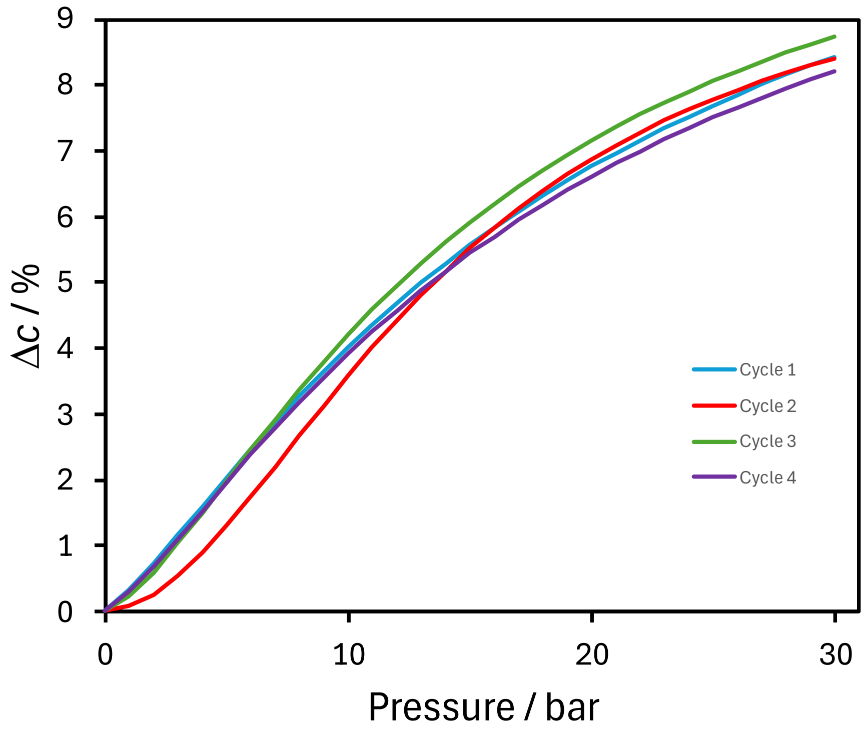

Supplementary Fig. 19. Sips models for the percentage expansion of the needle axis of Crystal 2 with increasing CO_2 pressure for all four cycles.

<u>Supplementary Text 13.3. Crystal 3</u>

Program: Five cycles of 0 → 30 → 0 bar at 0.2 bar min^{-1}.

Video file: Video 10 (first three cycles only)

Comments: The crystal was attached to a glass fiber by means of epoxy resin at approximately the midpoint along its needle axis, initially leaving both ends free of epoxy. This experiment was carried out to investigate whether the results would be different if one end of the crystal is not covered by epoxy resin (as for Crystals 1 and 2). Three cycles were recorded with both ends of the crystal free from epoxy (Supplementary Fig. 20). This was followed by one cycle with only one end of the crystal coated with epoxy (Supplementary Fig. 21a), followed by another cycle with both ends coated (Supplementary Fig. 21b). Best Sips fit models for increasing pressure for all five cycles are plotted in Supplementary Fig. 22, which shows little variation between the cycles, even when one or both ends of the crystal are coated in epoxy resin.

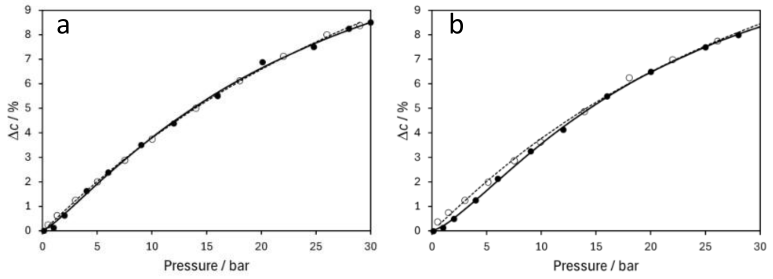

Supplementary Fig. 20. (a)–(c) Plots of percentage expansion of the needle axis *c* of Crystal 3 with CO_2 pressure during cycles 1 to 3, respectively. Solid and open circles represent data recorded during increasing and decreasing pressure, respectively. The best-fit Sips models are shown as solid (increasing pressure) and dashed (decreasing pressure) lines.

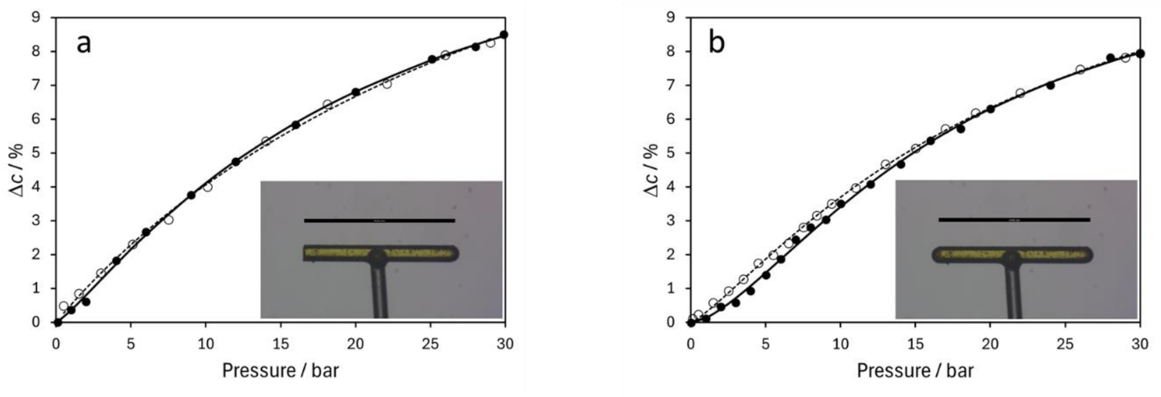

Supplementary Fig. 21. (a) and (b) Plots of percentage expansion of the needle axis *c* of Crystal 3 with CO_2 pressure during cycles 4 and 5, respectively. One end of the crystal was coated in epoxy resin before cycle 4, and then the other end was coated before cycle 5. Insets show photographs of the crystal with epoxied ends. Solid and open circles represent data recorded during increasing and decreasing pressure, respectively. The best-fit Sips models are shown as solid (increasing pressure) and dashed (decreasing pressure) lines.

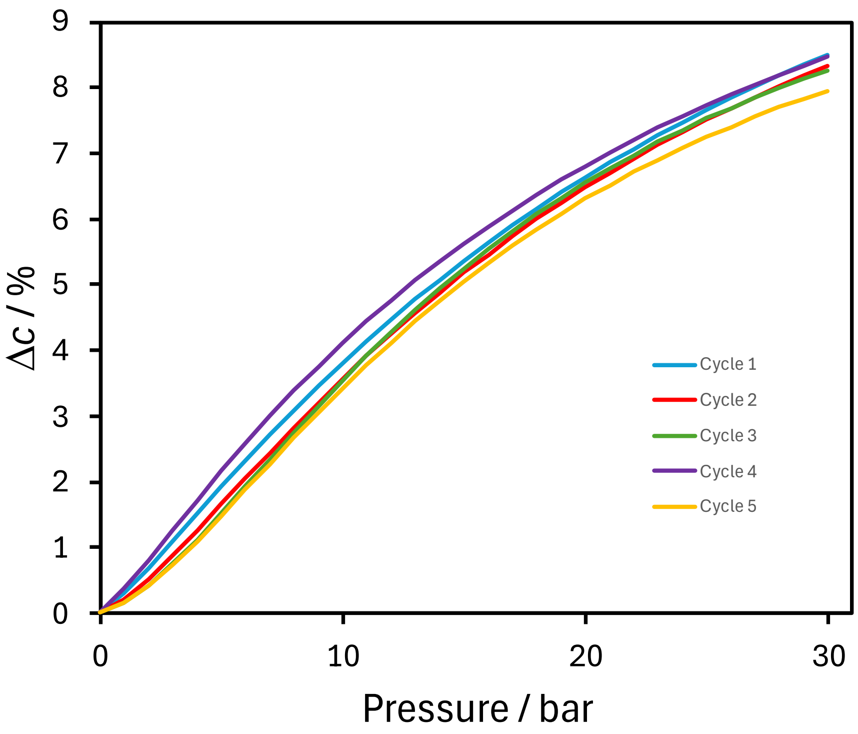

Supplementary Fig. 22. Sips models for the percentage expansion of the needle axis of Crystal 2 with increasing CO_2 pressure for all five cycles.

<u>Supplementary Text 13.4. Crystal 4</u>

Program: One cycle of 0 → 50 → 0 bar at 0.2 bar min^{-1}.

Video file: Video 11

Comments: The crystal was attached to a glass fiber by means of epoxy resin at approximately the midpoint along its needle axis, with both ends free of epoxy. This experiment was carried out to test whether the crystal maintains its structural integrity above 30 bar, and only one cycle was recorded (Supplementary Fig. 23). Deterioration of the transparency of part of the crystal became apparent at

approximately 40 bar. The crystal continued to disintegrate thereafter, and even during desorption. Data for the expansion of the needle axis were recorded from 0 to 50 bar, and then from 50 bar to 22 bar. Thereafter, severe deformation of the crystal was observed and no further expansion data were measured.

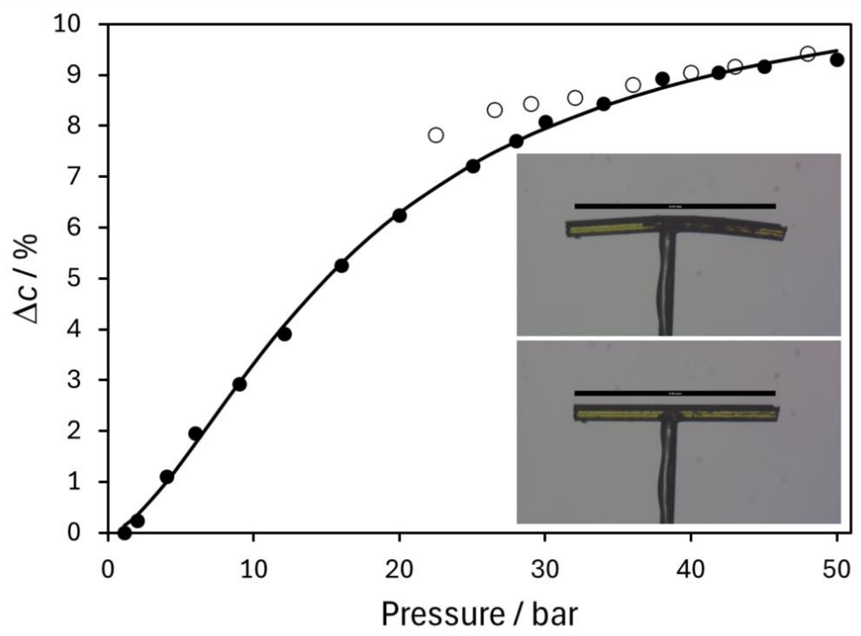

Supplementary Fig. 23. Plot of percentage expansion of the needle axis *c* of Crystal 4 with CO_2 pressure in the range 0 to 50 bar. Solid and open circles represent data recorded during increasing and decreasing pressure, respectively. The best-fit Sips model for adsorption is shown as a solid line. Inset bottom: photomicrograph of Crystal 4 at 0 bar; Inset top: photomicrograph of Crystal 4 at 22 bar, showing bending due to propagation of friability.

<u>**Supplementary Text 13.5. Crystal 5**</u>

Program: One cycle of 0 → 40 → 0 bar at 0.2 bar min^{-1}.

Video file: Video 12

Comments: The crystal was attached to a glass fiber by means of epoxy resin at approximately the midpoint along its needle axis, with both ends free of epoxy. This experiment was carried out in the widest pressure range during which the crystal remains undamaged by CO_2 pressure (as inferred from the experiments carried out with Crystal 4). The data presented in Supplementary Fig. 24 show excellent agreement between expansion and contraction of the needle axis during adsorption and desorption, respectively.

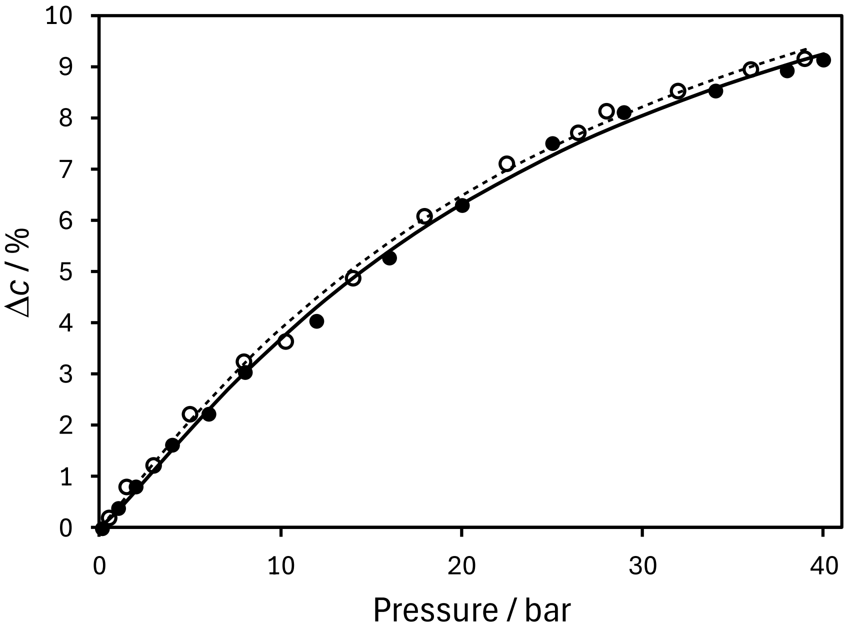

Supplementary Fig. 24. Plot of percentage expansion and contraction of the needle axis *c* of Crystal 5 with CO_2 pressure in the range 0 to 40 bar. Solid and open circles represent data recorded during increasing and decreasing pressure, respectively. The best-fit Sips model for adsorption is shown as a solid line.

Supplementary Text 13.6. Comparison between different crystals

The best Sips models for the percentage extension of the needle axes of all five crystals during increasing CO_2 pressure are shown in Supplementary Fig. 25, together with the corresponding data for the crystallographic *c* axis measured by X-ray diffraction analysis.

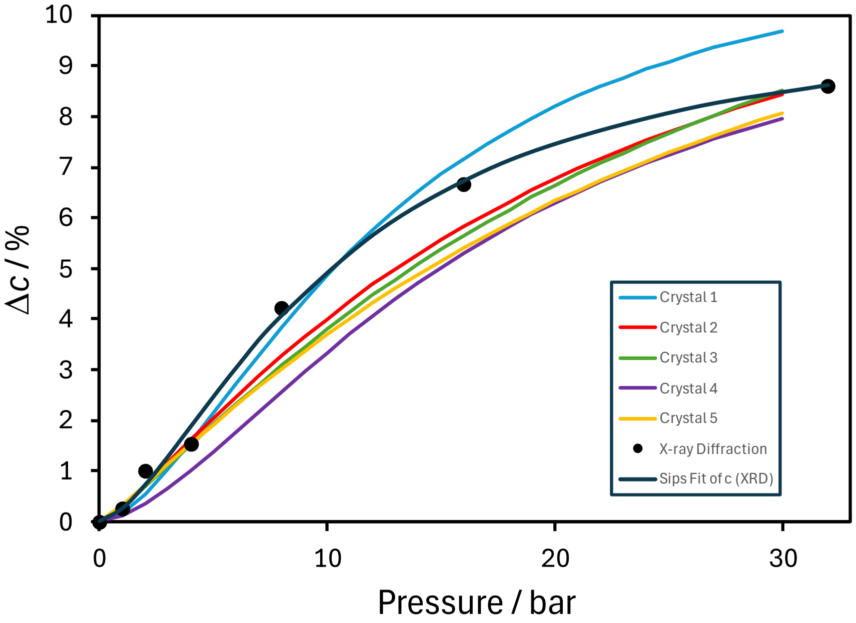

Supplementary Fig. 25. Best-fit Sips plots of percentage expansion and contraction of the needle axis *c* of Crystals 1 to 5 with increasing CO_2 pressure in the range 0 to 30 bar. Corresponding experimental data and the Sips model for elongation of the crystallographic *c* axis are shown as filled black circles a solid black line, respectively.

Supplementary Text 14. Methane

Supplementary Text 14.1. Gas sorption analysis

Methane (99.95%) gas cylinders were purchased from Afrox (LTD). Supplementary Fig. 26 shows a comparison between the uptake/release of CO_2 and CH_4 by crystals of **T1**. The experimental and fitted data for adsorption of CH_4 are shown in Supplementary Table 6.

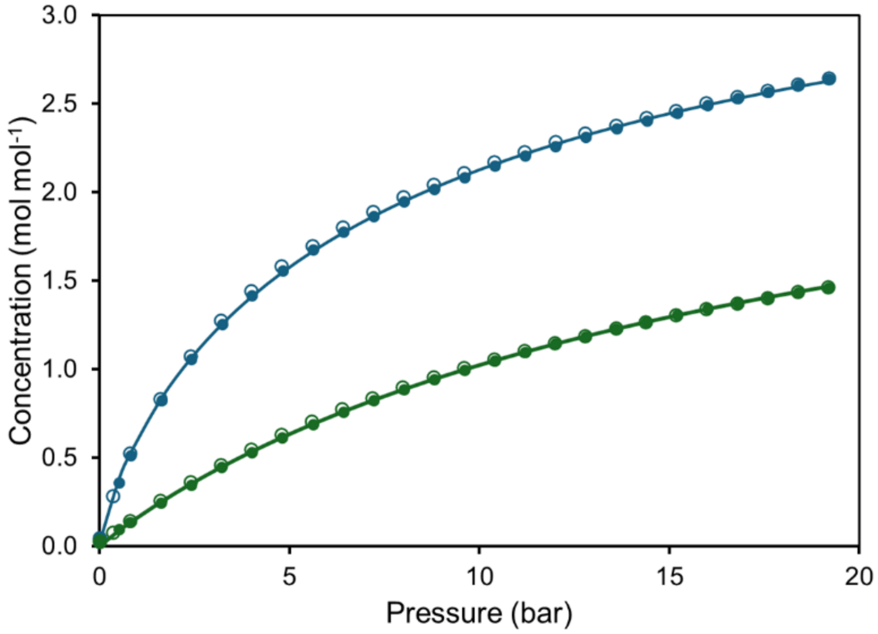

Supplementary Fig. 26. Absolute gas sorption isotherms for uptake and release of CO_2 (blue) and CH_4 (green) by crystals of **T1**. Filled and open circles represent sorption and desorption, respectively. Solid lines represent the best-fit Sips model for sorption in each case.

Supplementary Table 6. Experimental and Sips-fitted data for adsorption of CH_4 by crystals of **T1**.

P_{eq} / bar	n_{ads} / mol mol^{-1}	n_{fit} / mol mol^{-1}	R^2
0.0005	0.0226	0.0001	5.06E-04
0.0119	0.0252	0.0024	5.18E-04
0.4990	0.0936	0.0852	7.06E-05
0.7989	0.1364	0.1318	2.11E-05
1.6093	0.2449	0.2480	9.53E-06
2.4093	0.3480	0.3515	1.28E-05
3.2070	0.4472	0.4460	1.43E-06
4.0095	0.5315	0.5334	3.49E-06
4.8083	0.6119	0.6139	3.81E-06
5.6082	0.6873	0.6888	2.05E-06
6.4088	0.7575	0.7586	1.25E-06
7.2065	0.8225	0.8237	1.41E-06
8.0065	0.8849	0.8849	8.58E-11
8.8051	0.9427	0.9423	1.66E-07
9.6055	0.9968	0.9965	1.03E-07
10.4043	1.0484	1.0475	6.81E-07
11.2015	1.0959	1.0957	3.14E-08
12.0034	1.1432	1.1416	2.71E-06
12.7999	1.1857	1.1848	7.97E-07
13.5990	1.2274	1.2261	1.95E-06
14.3977	1.2670	1.2652	2.95E-06
15.1976	1.3074	1.3026	2.24E-05
16.0022	1.3438	1.3385	2.79E-05
16.8071	1.3713	1.3728	2.07E-06
17.5998	1.4022	1.4050	8.30E-06
18.3965	1.4323	1.4361	1.42E-05
19.1949	1.4623	1.4659	1.30E-05

R^2 is the square of the residual difference between n_{fit} and n_{ads}

ΣR^2	n_m	K	m
0.00125	2.91415	0.05276	0.96301

Supplementary Text 14.2. Van der Waals radius of CH_4.

An idealized CH_4 molecule was constructed *in silico* using the bond lengths and angles shown in Supplementary Fig. 27. Van der Waals radii for the elements (C, 1.70 Å; H, 1.20 Å) were taken from Bondi[55] and bond lengths were taken from https://cccbdb.nist.gov/exp2x.asp?casno=74828&charge=0. The van der Waals volume of the molecule (i.e., the volume of the contact surface mapped by MSRoll[52] using a virtual probe of radius 0 Å) was determined be 28.3 Å^3.

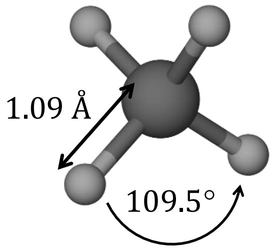

Supplementary Fig. 27. CH_4 model used to determine its van der Waals volume, with salient geometrical parameters shown.

Supplementary Text 14.3. Variable-pressure *in situ* X-ray crystallography

Variable-pressure single-crystal X-ray diffraction intensity data were recorded for samples exposed to CH_4 in the pressure range 0 to 60 bar at a temperature of 300 K. Selected crystallographic data for the VP-SCD structures are provided in Supplementary Table 7 and salient structural parameters are summarized in Supplementary Table 8 and displayed graphically in Supplementary Fig. 28.

For the crystallographic model at 60 bar the volumes of the two unique 0D voids are 69 and 88 A^3, with residual electron counts of 10 and 15, respectively. We have already addressed the reliability of electron counts using weak diffraction data, and these should thus be regarded as indicative rather than conclusive. The two void volumes are both sufficient to accommodate the inclusion of one molecule of CH_4 each.

Photomicrographs were recorded at 30 s intervals for a single crystal in the range 0–40–0 bar CH_4 and used to construct Supplementary Video 13.

Supplementary Table 7. Crystal data and structure refinement parameters for structural analyses of **T1** crystals under vacuum and CH_4 pressure.

Identification code	**T1$_0$**	**T1$_{M10}$**	**T1$_{M20}$**	**T1$_{M30}$**
Gas pressure (bar)	0	10	20	30
Empirical formula	$C_{42}H_{48}N_6O_6$	$C_{42}H_{48}N_6O_6\cdot CH_4$	$C_{42}H_{48}N_6O_6\cdot 1.4(CH_4)$	$C_{42}H_{48}N_6O_6\cdot 1.7(CH_4)$
Formula weight	732.86	748.90	755.32	760.13
Temperature (K)	300(2)	300(2)	300(2)	300(2)
Wavelength (Å)	0.71073	0.71073	0.71073	0.71073
Crystal system	trigonal	trigonal	trigonal	trigonal
Space group	*R*3:H	*R*3:H	*R*3:H	*R*3:H
Unit cell dimensions (Å)	$a = b = 50.7988(11)$	$a = b = 50.8720(13)$	$a = b = 50.7480(13)$	$a = b = 50.7195(11)$
	$c = 9.6206(4)$	$c = 9.7345(4)$	$c = 9.8079(4)$	$c = 9.8635(4)$
Volume (Å^3)	21500.1(13)	21817.3(14)	21874.8(14)	21974.1(13)
Z	18	18	18	18
Calculated density (g cm^{-3})	1.019	1.026	1.032	1.034
Absorption coefficient (mm^{-1})	0.069	0.069	0.070	0.070
F_{000}	7020	7200	7272	7326
Crystal size (mm^3)	0.391 × 0.090 × 0.085	0.391 × 0.090 × 0.085	0.391 × 0.090 × 0.085	0.391 × 0.090 × 0.085
θ range for data collection (°)	2.121 to 22.007	2.118 to 22.002	2.124 to 22.000	2.116 to 21.977
Miller index ranges	$-53 \le h \le 53$, $-53 \le k \le 53$, $-10 \le l \le 10$	$-53 \le h \le 53$, $-52 \le k \le 53$, $-10 \le l \le 10$	$-53 \le h \le 52$, $-51 \le k \le 53$, $-10 \le l \le 10$	$-53 \le h \le 53$, $-53 \le k \le 53$, $-10 \le l \le 10$
Reflections collected	75511	77403	70753	74557
Independent reflections	11708 [Rint = 0.1425]	11869 [Rint = 0.1684]	11929 [Rint = 0.1559]	11963 [Rint = 0.1409]
Completeness to θ_{max} (%)	0.999	0.998	0.999	0.999
Max. and min. transmission	0.945 and 1.000	0.940 and 1.000	0.932 and 1.000	0.939 and 1.000
Refinement method	Full-matrix least-squares on F^2	Full-matrix least-squares on F^2	Full-matrix least-squares on F^2	Full-matrix least-squares on F^2
Data / restraints / parameters	11708 / 1597 / 985	11869 / 1597 / 985	11929 / 1597 / 985	11963 / 1597 / 985
Goodness-of-fit on F^2	1.035	1.003	1.017	1.033
Final *R* indices [$I > 2\sigma(I)$]	$R1 = 0.0792$, $wR2 = 0.1695$	$R1 = 0.0938$, $wR2 = 0.2178$	$R1 = 0.0981$, $wR2 = 0.2281$	$R1 = 0.0992$, $wR2 = 0.2330$
R indices (all data)	$R1 = 0.2083$, $wR2 = 0.2341$	$R1 = 0.2428$, $wR2 = 0.3096$	$R1 = 0.2454$, $wR2 = 0.3209$	$R1 = 0.2296$, $wR2 = 0.3180$
Largest diff. peak and hole (e Å^{-3})	0.294 and -0.228	0.630 and -0.227	0.759 and -0.213	0.711 and -0.265
Absolute structure parameter	0.3(9)	0.6(10)	0.1(9)	0.4(8)

Supplementary Table 7. (continued)

Identification code	**T1$_{M40}$**	**T1$_{M50}$**	**T1$_{M60}$**
Gas pressure (bar)	40	50	60
Empirical formula	$C_{42}H_{48}N_6O_6$·1.95(CH_4)	$C_{42}H_{48}N_6O_6$·2(CH_4)	$C_{42}H_{48}N_6O_6$·2.2(CH_4)
Formula weight	764.14	764.94	768.15
Temperature (K)	300(2)	300(2)	300(2)
Wavelength (Å)	0.71073	0.71073	0.71073
Crystal system	trigonal	trigonal	trigonal
Space group	*R*3:H	*R*3:H	*R*3:H
Unit cell dimensions (Å)	$a = b$ = 50.6671(11)	$a = b$ = 50.5703(12)	$a = b$ = 50.4664(11)
	c = 9.9130(4)	c = 9.9592(4)	c = 10.0272(4)
Volume (Å^3)	22038.8(13)	22057.0(14)	22116.4(13)
Z	18	18	18
Calculated density (g cm^{-3})	1.036	1.037	1.038
Absorption coefficient (mm^{-1})	0.070	0.070	0.070
F_{000}	7371	7380	7416
Crystal size (mm^3)	0.391 × 0.090 × 0.085	0.391 × 0.090 × 0.085	0.391 × 0.090 × 0.085
θ range for data collection (°)	2.106 to 22.004	2.097 to 21.977	2.084 to 21.986
Miller index ranges	$-53 \le h \le 53$, $-53 \le k \le 53$, $-10 \le l \le 10$	$-53 \le h \le 53$, $-53 \le k \le 53$, $-10 \le l \le 10$	$-53 \le h \le 52$, $-48 \le k \le 53$, $-10 \le l \le 10$
Reflections collected	73851	76318	75829
Independent reflections	12014 [*R*int = 0.1508]	12005 [*R*int = 0.1571]	12032 [*R*int = 0.1655]
Completeness to θ_{max} (%)	0.998	0.999	0.999
Max. and min. transmission	0.927 and 1.000	0.858 and 1.000	0.912 and 1.000
Refinement method	Full-matrix least-squares on F^2	Full-matrix least-squares on F^2	Full-matrix least-squares on F^2
Data / restraints / parameters	12014 / 1597 / 985	12005 / 1597 / 985	12032 / 1597 / 985
Goodness-of-fit on F^2	1.009	1.000	0.993
Final *R* indices [$I > 2\sigma(I)$]	$R1$ = 0.1056, $wR2$ = 0.2504	$R1$ = 0.1044, $wR2$ = 0.2479	$R1$ = 0.1057, $wR2$ = 0.2491
R indices (all data)	$R1$ = 0.2461, $wR2$ = 0.3459	$R1$ = 0.2484, $wR2$ = 0.3452	$R1$ = 0.2564, $wR2$ = 0.3495
Largest diff. peak and hole (e Å^{-3})	0.820 and -0.207	0.791 and -0.235	0.868 and -0.197
Absolute structure parameter	0.8(9)	0.3(9)	0.2(10)

Supplementary Table 8. Selected structural parameters for **T1**$_{\mathbf{M}x}$.

Pressure / bar	a / Å	c / Å	V_{cell} / Å^3	$V_{channel}$ / Å^3	%Δa	%Δc	%ΔV_{cell}	%Δ$V_{channel}$
0	50.799	9.621	21,500	974	0.00	0.00	0.00	0.00
10	50.872	9.735	21,817	986	0.14	1.18	1.47	1.23
20	50.748	9.808	21,875	990	-0.10	1.95	1.74	1.64
30	50.720	9.864	21,974	1,006	-0.16	2.52	2.20	3.29
40	50.667	9.913	22,039	1,016	-0.26	3.04	2.51	4.31
50	50.570	9.959	22,057	1,024	-0.45	3.52	2.59	5.13
60	50.466	10.027	22,116	1,052	-0.65	4.23	2.87	8.01

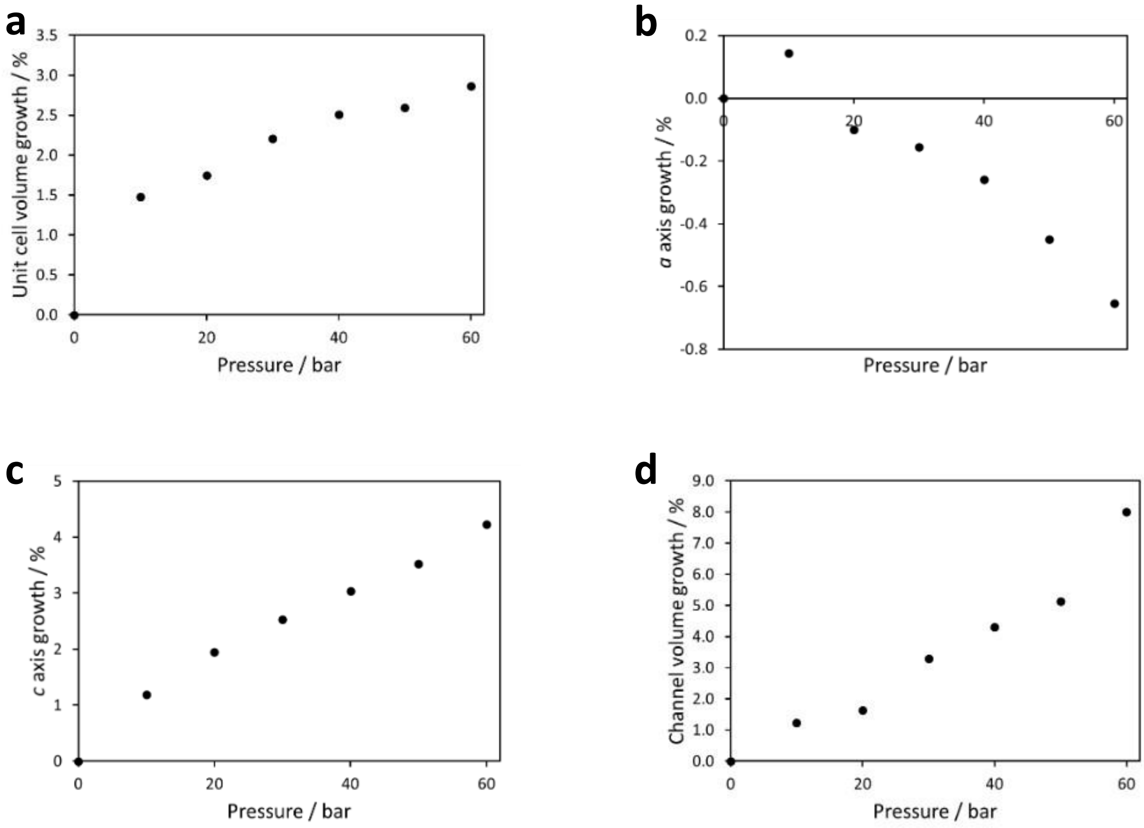

Supplementary Fig. 28. Plots of changes in selected lengths and volumes for the structures **T1**$_{\mathbf{M}x}$ with pressure. (a) Unit cell volume, (b) unit cell axis a, (c) unit cell axis c and (d) the volume of the channel that propagates along [001].

Supplementary Video Captions

Supplementary Video 1. Projections of an entire unit cell of the structure series **T1**$_{Cx}$ along [001]. Probe-accessible space is shown as yellow surfaces (probe radius 1.5 Å, grid spacing 0.2 Å). The sequence shows enlargement of the 0D voids of the time-averaged crystal structures with increasing CO_2 pressure. Images were created using Mercury.

Supplementary Video 2. Alternating spacefilling diagrams of **T1**$_0$ and **T1**$_{C32}$ projected along [001]. Six molecules of **T1** are shown, forming a ring that stacks along [001] to create 1 nm wide 1D channels. The video is best played with the repeat setting turned on and contrasts the orientations of the molecules at the two pressure extremes for which structural data are available. Images were created using X-Seed and POV-Ray.

Supplementary Video 3. Spacefilling diagrams of **T1**$_{Cx}$ projected along [001]. Six molecules of **T1** are shown, forming a ring that stacks along [001] to create 1 nm wide 1D channels. The video shows the progression of the orientations of the molecules as the gas pressure is increased from 0 to 32 bar. Images were created using X-Seed and POV-Ray.

Supplementary Video 4. Projections of six successive unit cells of the structure series **T1**$_{Cx}$ along [010]. Probe-accessible space is shown as yellow surfaces (probe radius 1.5 Å, grid spacing 0.2 Å). The sequence shows enlargement of the 1D channels in the time-averaged crystal structures as the **T1** molecules move apart to create space for the gas molecules within the 0D voids (voids omitted for clarity). Images were created using Mercury.

Supplementary Video 5. Alternating capped-stick diagrams showing the formation of two symmetry-independent 0D voids in **T1**$_0$ and **T1**$_{C32}$. Each void primarily consists of the intrinsic cavity of a molecule of **T1** and is capped by cyclohexane moieties of neighboring host molecules. Crystallographically independent molecules are colored blue and green. Probe-accessible space (blue surfaces) was calculated using MSRoll *via* the X-Seed interface (probe radius 1.5 Å). The alternating images show overall enlargement of the two distinct cavities between 0 and 32 bar of CO_2 pressure. Images were created using X-Seed and the video is best played with the repeat setting turned on.

Supplementary Video 6. Packing of a simplified model (see Supplementary Fig. 9) of **T1** in the structures **T1**$_{Cx}$, viewed perpendicular to [001]. The triangles represent hexameric assemblies of **T1** molecules and five successive assemblies are shown stacked along [001]. Red and grey triangles distinguish between crystallographically-independent molecules. The sequence shows how the molecules tilt slightly relative to [001] with increasing pressure, thus causing elongation of the periodicity along the crystallographic *c* axis. Images were created using X-Seed.

Supplementary Video 7. Video sequence showing structural adjustment of **T1**$_{Cx}$ to increasing CO_2 pressure (left) and corresponding photomicrographs (right) of the crystal at the same pressures.

Supplementary Video 8. Time-lapse sequence showing Crystal 1 responding to variable CO_2 gas pressure. The video shows one cycle of ramping the pressure at a rate of 0.2 bar min^{-1} from 0 to 32 bar, and then back to 0 bar. The dashed vertical line on the left indicates where the crystal is attached to a glass fiber and the dashed vertical line on the right facilitates observation, relative to **T1**$_0$, of gas-induced elongation along [001].

Supplementary Video 9. Time-lapse sequence showing Crystal 2 responding to variable CO_2 gas pressure. The video shows one cycle of ramping the pressure at a rate of 0.2 bar min^{-1} from 0 to 30 bar, and then back to 0 bar.

Supplementary Video 10. Time-lapse sequence showing Crystal 3 responding to variable CO_2 gas pressure. The video shows three cycles of ramping the pressure at a rate of 0.2 bar min^{-1} from 0 to 30 bar, and then back to 0 bar.

Supplementary Video 11. Time-lapse sequence showing Crystal 4 responding to variable CO_2 gas pressure. The video shows one cycle of ramping the pressure at a rate of 0.2 bar min^{-1} from 0 to 50 bar, and then back to 0 bar.

Supplementary Video 12. Time-lapse sequence showing Crystal 5 responding to variable CO_2 gas pressure. The video shows one cycle of ramping the pressure at a rate of 0.2 bar min^{-1} from 0 to 40 bar, and then back to 0 bar.

Supplementary Video 13. Time-lapse sequence showing Crystal 6 responding to variable CH_4 gas pressure. The video shows one cycle of ramping the pressure at a rate of 0.2 bar min^{-1} from 0 to 40 bar, and then back to 0 bar.

References

47. Okada, Y., Sugai, M. & Chiba, K. Hydrogen-Bonding-Induced Fluorescence: Water-Soluble and Polarity-Independent Solvatochromic Fluorophores. *J. Org. Chem.* **81**, 10922–10929 (2016).

48. Szymkowiak, J. & Kwit, M. Electronic and vibrational exciton coupling in oxidized trianglimines. *Chirality* **30**, 117–130 (2018).

49. Kuhnert, N., Rossignolo, G. M. & Lopez-Periago, A. The synthesis of trianglimines: on the scope and limitations of the [3 + 3] cyclocondensation reaction between (1R,2R)-diaminocyclohexane and aromatic dicarboxaldehydes. *Org. Biomol. Chem.* **1**, 1157–1170 (2003).

50. Eaby, A. C. *et al.* Dehydration of a crystal hydrate at subglacial temperatures. *Nature* **616**, 288–292 (2023).

51. MacRae, C. F. *et al.* Mercury 4.0: From visualization to analysis, design and prediction. *J. Appl. Crystallogr.* **53**, 226–235 (2020).

52. Connolly, M. L. Solvent-Accessible Surfaces of Proteins and Nucleic Acids. *Science* **221**, 709–713 (1983).

53. Barbour, L. J. X-Seed — A Software Tool for Supramolecular Crystallography. *J. Supramol. Chem.* **1**, 189–191 (2001).

54. Barbour, L. J. X-Seed 4: Updates to a program for small-molecule supramolecular crystallography. *J. Appl. Cryst.* **53**, 1141–1146 (2020).

55. Bondi, A. van der Waals Volumes and Radii. *J. Phys. Chem.* **68**, 441–451 (1964).

56. Benham, M. J. & Ross, D. K. Experimental Determination of Absorption-Desorption Isotherms by Computer-Controlled Gravimetric Analysis. *Z. Phys. Chem.* **163**, 25–32 (1989).

57. Fletcher, A. J. & Thomas, K. M. Adsorption and Desorption Kinetics of n-Octane and n-Nonane Vapors on Activated Carbon. *Langmuir* **15**, 6908–6914 (1999).

58. Reid, C. R. & Thomas, K. M. Adsorption of gases on a carbon molecular sieve used for air separation: Linear adsorptives as probes for kinetic selectivity. *Langmuir* **15**, 3206–3218 (1999).

59. Fletcher, A. J. & Thomas, K. M. Compensation effect for the kinetics of adsorption/desorption of gases/vapors on microporous carbon materials. *Langmuir* **16**, 6253–6266 (2000).

60. O'koye, I. P., Benham, M. & Thomas, K. M. Adsorption of gases and vapors on carbon molecular sieves. *Langmuir* **13**, 4054–4059 (1997).

61. Sips, R. On the structure of a catalyst surface. *J. Chem. Phys.* **16**, 490–495 (1948).

62. SAINT Data Reduction Software. *Version 6.45, Bruker AXS Incl* (2003).

63. Bruker, SADABS. *Bruker AXS Incl* (2001).

64. Sheldrick, G. M. Crystal structure refinement with SHELXL. *Acta. Cryst. C* **71**, 3–8 (2015).

65. Atwood, J. L. & Barbour, L. J. Molecular Graphics: From Science to Art. *Cryst. Growth Des.* **3**, 3–8 (2003).

66. Groom, C. R., Bruno, I. J., Lightfoot, M. P. & Ward, S. C. The Cambridge structural database. *Acta Crystallogr. B Struct. Sci. Cryst. Eng. Mater.* **72**, 171–179 (2016).

67. Zhang, J. P. & Chen, X. M. Optimized acetylene/carbon dioxide sorption in a dynamic porous crystal. *J. Am. Chem. Soc.* **131**, 5516–5521 (2009).

68. Takamizawa, S., Nakata, E. I. & Saito, T. Structural determination of copper(II) benzoate-pyrazine containing carbon dioxide molecules. *Inorg. Chem. Commun.* **7**, 1–3 (2004).

69. Takamizawa, S. *et al.* Crystal transformation and host molecular motions in CO2 adsorption process of a metal benzoate pyrazine (MII= Rh, Cu). *J. Am. Chem. Soc.* **132**, 3783–3792 (2010).

70. Pajuelo-Corral, O. *et al.* A metal-organic framework based on Co(II) and 3-aminoisonicotinate showing specific and reversible colourimetric response to solvent exchange with variable magnet behaviour. *Mater. Today Chem.* **24**, (2022).

71. Kosaka, W. *et al.* Densely Packed CO2 Aids Charge, Spin, and Lattice Ordering Partially Fluctuated in a Porous Metal–Organic Framework Magnet. *Angew. Chem., Int. Ed.* **62**, 1–7 (2023).

72. Xu, W. *et al.* Fabrication of Pillar-Cage Fluorinated Anion Pillared Metal–Organic Frameworks via a Pillar Embedding Strategy and Efficient Separation of SO2 through Multi-Site Trapping. *Angew. Chem., Int. Ed.* **62**, 2–9 (2023).

73. Zhu, X. *et al.* Vertex Strategy in Layered 2D MOFs: Simultaneous Improvement of Thermodynamics and Kinetics for Record C2H2/CO2 Separation Performance. *J. Am. Chem. Soc.* **145**, 9254–9263 (2023).

74. Maji, T. K., Mostafa, G., Matsuda, R. & Kitagawa, S. Guest-induced asymmetry in a metal-organic porous solid with reversible single-crystal-to-single-crystal structural transformation. *J. Am. Chem. Soc.* **127**, 17152–17153 (2005).

75. Kane, C. M., Ugono, O., Barbour, L. J. & Holman, K. T. Many Simple Molecular Cavitands Are Intrinsically Porous (Zero-Dimensional Pore) Materials. *Chem. Mater.* **27**, 7337–7354 (2015).

76. Brekalo, I. *et al.* Microporosity of a Guanidinium Organodisulfonate Hydrogen-Bonded Framework. *Angew. Chem., Int. Ed.* **59**, 1997–2002 (2020).

77. Qazvini, O. T., Babarao, R. & Telfer, S. G. Selective capture of carbon dioxide from hydrocarbons using a metal-organic framework. *Nat. Commun.* **12**, 1–8 (2021).

78. Tsue, H. *et al.* Crystallographic analysis of CO2 sorption state in seemingly nonporous molecular crystal of azacalix[4]arene tetramethyl ether exhibiting highly selective CO2 uptake. *CrystEngComm* **14**, 1021–1026 (2012).

79. Bolotov, V. A. *et al.* Enhancement of CO2 Uptake and Selectivity in a Metal-Organic Framework by the Incorporation of Thiophene Functionality. *Inorg. Chem.* **57**, 5074–5082 (2018).

80. Wu, P. *et al.* Carbon dioxide capture and efficient fixation in a dynamic porous coordination polymer. *Nat. Commun.* **10**, 1–8 (2019).

81. Chen, M. *et al.* Analyzing Gas Adsorption in an Amide-Functionalized Metal Organic Framework: Are the Carbonyl or Amine Groups Responsible? *Chem. Mater.* **30**, 3613–3617 (2018).

82. Takamizawa, S., Nakata, E. I., Yokoyama, H., Mochizuki, K. & Mori, W. Carbon dioxide inclusion phases of a transformable 1D coordination polymer host [Rh2(O2CPh)4(pyz)]n. *Angew. Chem., Int. Ed.* **42**, 4331–4334 (2003).

83. Chen, S., Lucier, B. E. G., Boyle, P. D. & Huang, Y. Understanding the Fascinating Origins of CO2 Adsorption and Dynamics in MOFs. *Chem. Mater.* **28**, 5829–5846 (2016).

84. Forse, A. C. *et al.* Unexpected Diffusion Anisotropy of Carbon Dioxide in the Metal-Organic Framework Zn2(dobpdc). *J. Am. Chem. Soc.* **140**, 1663–1673 (2018).

85. Takamizawa, S., Kojima, K. & Akatsuka, T. Channel-switching crystal with guest stress drive. *Inorg. Chem.* **45**, 4580–4582 (2006).

86. Sikiti, P., Bezuidenhout, C. X., Van Heerden, D. P. & Barbour, L. J. Direct in Situ Crystallographic Visualization of a Dual Mechanism for the Uptake of CO2 Gas by a Flexible Metal-Organic Framework. *Inorg. Chem.* **58**, 8257–8262 (2019).

87. Lama, P., Rawat, A., Sikiti, P. & Pal, T. K. Significance of an Environmental Gas Cell to Obtain a Fully Dehydrated Form and CO2-Pressurized Structure of a Metal-Organic Framework Using in Situ Single-Crystal X-ray Diffraction at 298 K. *Inorg. Chem.* **61**, 939–943 (2022).

88. Nikolayenko, V. I. *et al.* Reversible transformations between the non-porous phases of a flexible coordination network enabled by transient porosity. *Nat. Chem.* **15**, 542–549 (2023).

89. Yuan, Z. *et al.* Sticked-Layer Strategy to a Flexible-Robust Hydrogen-Bonded Organic Framework for Efficient C2H2/CO2 Separation. *CCS Chem.* **6**, 663–671 (2024).

90. Lama, P. & Barbour, L. J. Distinctive Three-Step Hysteretic Sorption of Ethane with in Situ Crystallographic Visualization of the Pore Forms in a Soft Porous Crystal. *J. Am. Chem. Soc.* **140**, 2145–2150 (2018).

91. Takamizawa, S., Akatsuka, T. & Ueda, T. Gas-conforming transformability of an ionic single-crystal host consisting of discrete charged components. *Angew. Chem., Int. Ed.* **47**, 1689–1692 (2008).

92. Nikolayenko, V. I., Castell, D. C., van Heerden, D. P. & Barbour, L. J. Guest-Induced Structural Transformations in a Porous Halogen-Bonded Framework. *Angew. Chem., Int. Ed.* **57**, 12086–12091 (2018).

93. Shekhah, O. *et al.* The liquid phase epitaxy approach for the successful construction of ultra-thin and defect-free ZIF-8 membranes: Pure and mixed gas transport study. *Chem, Commun,* **50**, 2089–2092 (2014).

94. van Heerden, D. P., Smith, V. J., Aggarwal, H. & Barbour, L. J. High Pressure In Situ Single-Crystal X-Ray Diffraction Reveals Turnstile Linker Rotation Upon Room-Temperature Stepped Uptake of Alkanes. *Angew. Chem., Int. Ed.* **60**, 13430–13435 (2021).

95. Geng, S. *et al.* Scalable Room-Temperature Synthesis of Highly Robust Ethane-Selective Metal-Organic Frameworks for Efficient Ethylene Purification. *J. Am. Chem. Soc.* **143**, 8654–8660 (2021).

96. Liu, W. *et al.* Highly Robust Microporous Metal-Organic Frameworks for Efficient Ethylene Purification under Dry and Humid Conditions. *Angew. Chem., Int. Ed.* **62**, (2023).

97. Lama, P., Aggarwal, H., Bezuidenhout, C. X. & Barbour, L. J. Giant Hysteretic Sorption of CO2: In Situ Crystallographic Visualization of Guest Binding within a Breathing Framework at 298 K. *Angew. Chem., Int. Ed.* **55**, 13271–13275 (2016).

98. Zheng, X. *et al.* Understanding the interactions between the bis(trifluoromethylsulfonyl)imide anion and absorbed CO2 using X-ray diffraction analysis of a soft crystal surrogate. *Commun. Chem.* **3**, 1–7 (2020).

99. Wriedt, M. *et al.* Low-energy selective capture of carbon dioxide by a pre-designed elastic single-molecule trap. *Angew. Chem., Int. Ed.* **51**, 9804–9808 (2012).

100. Queen, W. L. *et al.* Comprehensive study of carbon dioxide adsorption in the metal-organic frameworks M2(dobdc) (M = Mg, Mn, Fe, Co, Ni, Cu, Zn). *Chem. Sci.* **5**, 4569–4581 (2014).

101. Briggs, L. *et al.* Binding and separation of CO2, SO2 and C2H2 in homo- and hetero-metallic metal-organic framework materials. *J. Mater. Chem. A Mater.* **9**, 7190–7197 (2021).

102. Benson, O. *et al.* Amides Do Not Always Work: Observation of Guest Binding in an Amide-Functionalized Porous Metal-Organic Framework. *J. Am. Chem. Soc.* **138**, 14828–14831 (2016).

103. Krause, S. *et al.* A pressure-amplifying framework material with negative gas adsorption transitions. *Nature* **532**, 348–352 (2016).

104. Moreau, F. *et al.* Unravelling exceptional acetylene and carbon dioxide adsorption within a tetra-amide functionalized metal-organic framework. *Nat. Commun.* **8**, 1–9 (2017).

105. Shivanna, M. *et al.* Crossover Sorption of C2H2/CO2 and C2H6/C2H4 in Soft Porous Coordination Networks. *Angew. Chem., Int. Ed.* **62**, (2023).

106. Cadiau, A. *et al.* Molecular sorption: Hydrolytically stable fluorinated metal-organic frameworks for energy-efficient dehydration. *Science* **356**, 731–735 (2017).

107. Noro, S. I. *et al.* A Temporarily Pore-Openable Porous Coordination Polymer for Guest Adsorption/Desorption. *Inorg. Chem.* **60**, 4531–4538 (2021).

108. Banerjee, D. *et al.* Direct Structural Identification of Gas Induced Gate-Opening Coupled with Commensurate Adsorption in a Microporous Metal–Organic Framework. *Chem. Eur. J.* **22**, 11816–11825 (2016).

109. Chae, S. H. *et al.* Thermally robust 3-D Co-DpyDtolP-MOF with hexagonally oriented micropores: Formation of polyiodine chains in a MOF single crystal. *Cryst. Growth Des.* **15**, 268–277 (2015).

110. Li, J. *et al.* Guest-controlled incommensurate modulation in a meta-rigid metal-organic framework material. *J. Am. Chem. Soc.* **142**, 19189–19197 (2020).

111. Zhang, J., Kosaka, W., Kitagawa, Y. & Miyasaka, H. A metal–organic framework that exhibits CO2-induced transitions between paramagnetism and ferrimagnetism. *Nat. Chem.* **13**, 191–199 (2021).

112. Yao, Z. *et al.* Direct Evidence of CO2 Capture under Low Partial Pressure on a Pillared Metal–Organic Framework with Improved Stabilization through Intramolecular Hydrogen Bonding. *Chempluschem* **81**, 850–856 (2016).

113. Bhatt, P. M., Batisai, E., Smith, V. J. & Barbour, L. J. Creation of new guest accessible space under gas pressure in a flexible MOF: Multidimensional insight through combination of: In situ techniques. *Chem. Commun.* **52**, 11374–11377 (2016).

114. Dou, C., Kosaka, W. & Miyasaka, H. Gate-open-type sorption in a zigzag paddlewheel Ru dimer chain compound with a phenylenediamine linker instructed by a preliminary structural change of desolvation. *Chem. Lett.* **46**, 1288–1291 (2017).

115. Li, L. *et al.* Post-synthetic modulation of the charge distribution in a metal-organic framework for optimal binding of carbon dioxide and sulfur dioxide. *Chem. Sci.* **10**, 1472–1482 (2019).

116. Lu, Z. *et al.* Modulating supramolecular binding of carbon dioxide in a redox-active porous metal-organic framework. *Nat. Commun.* **8**, (2017).

117. Giménez-Marqués, M. *et al.* Gas confinement in compartmentalized coordination polymers for highly selective sorption. *Chem. Sci.* **8**, 3109–3120 (2017).

118. Wen, H. M. *et al.* A metal-organic framework with suitable pore size and dual functionalities for highly efficient post-combustion CO2 capture. *J. Mater. Chem. A Mater.* **7**, 3128–3134 (2019).

119. Cavallo, M. *et al.* Cooperative CO2 adsorption mechanism in a perfluorinated CeIV-based metal organic framework. *J. Mater. Chem. A Mater.* **11**, 5568–5583 (2023).

120. Evans, H. A. *et al.* Aluminum formate, Al(HCOO)3: An earth-abundant, scalable, and highly selective material for CO2 capture. *Sci. Adv.* **8**, (2022).

121. Kosaka, W. *et al.* Selective NO trapping in the pores of chain-type complex assemblies based on electronically activated paddlewheel-type [Ru2II,II]/ [Rh2II,II] dimers. *J. Am. Chem. Soc.* **135**, 18469–18480 (2013).

122. Jacobs, T., Smith, V. J., Thomas, L. H. & Barbour, L. J. Carbon dioxide entrapment in an organic molecular host. *Chem. Commun.* **50**, 85–87 (2014).

123. Atwood, J. L., Barbour, L. J., Jerga, A. & Schottel, B. L. Guest transport in a nonporous organic solid via dynamic van der Waals cooperativity. *Science* **298**, 1000–1002 (2002).

124. Atwood, J. L., Barbour, L. J. & Jerga, A. Storage of methane and freon by interstitial van der Waals confinement. *Science* **296**, 2367–2369 (2002).

125. Kane, C. M., Banisafar, A., Dougherty, T. P., Barbour, L. J. & Holman, K. T. Enclathration and Confinement of Small Gases by the Intrinsically 0D Porous Molecular Solid, Me,H,SiMe2. *J. Am. Chem. Soc.* **138**, 4377–4392 (2016).

126. Spek, A. L. PLATON SQUEEZE: a tool for the calculation of the disordered solvent contribution to the calculated structure factors. *Acta Crystallogr. C Struct. Chem.* **71**, 9–18 (2015).

127. Spek, A. L. Single-crystal structure validation with the program PLATON. *J. Appl. Cryst.* **36**, 7–13 (2003).

128. Langmuir, I. The Adsorption of gases on plane surfaces of glass, mica and platinum. *J. Am. Chem. Soc.* **40**, 1361–1403 (1918).

129. Tóth, J. Uniform interpretation of gas/solid adsorption. *Adv. Colloid. Interface Sci.* **55**, 1–239 (1995).
